# Enhancing phase I dose-finding trials design through dynamic borrowing information and handling late-onset toxicity

**DOI:** 10.3389/fphar.2023.1266322

**Published:** 2023-11-22

**Authors:** Wenyun Yang, Ruyue He, Yuehan Sun, Fangrong Yan, Fei Wang

**Affiliations:** ^1^ Research Center of Biostatistics and Computational Pharmacy, China Pharmaceutical University, Nanjing, China; ^2^ College of Management and Economics, Tianjin University, Tianjin, China

**Keywords:** dose-finding, supplemental data, late-onset toxicity, multisource exchangeability models, model-assisted designs

## Abstract

**Introduction:** In recent years, there has been a growing trend among regulatory agencies to consider the use of historical controls in clinical trials as a means of improving the efficiency of trial design. In this paper, to enhance the statistical operating characteristic of Phase I dose-finding trials, we propose a novel model-assisted design method named “MEM-Keyboard”.

**Methods:** The proposed design is based on the multisource exchangeability models (MEMs) that allows for dynamic borrowing of information from multiple supplemental data sources, including historical trial data, to inform the dose-escalation process. Furthermore, with the frequent occurrence of delayed toxicity in novel anti-cancer drugs, we extended our proposed method to handle late-onset toxicity by incorporating historical data. This extended method is referred to as “MEM-TITE-Keyboard” and aims to improve the efficiency of early clinical trials.

**Results:** Simulation studies have indicated that the proposed methods can improve the probability of correctly selecting the maximum tolerated dose (MTD) with an acceptable level of risk, compared to designs that do not account for information borrowing and late-onset toxicity.

**Discussion:** The MEM-Keyboard and MEM-TITE-Keyboard, easy to implement in practice, provide a useful tool for identifying MTD and accelerating drug development.

## 1 Introduction

In the realm of clinical trial design, early-phase trials play a pivotal role in the development of novel therapeutics, exerting considerable influence on the subsequent stages of the trial. A mass of novel Phase I trial designs have been proposed to identify the maximum tolerated dose (MTD) of a new drug more efficiently, defined as the dose with a dose-limiting toxicity (DLT) probability closest to a prespecified target probability. These proposed novel designs can be categorized as algorithm-based designs, model-based designs and model-assisted designs (Zhou et al., 2018). While maintaining the simplicity of trial design, the model-assisted designs, including Bayesian optimal interval and Keyboard designs, are superior to algorithm-based (e.g., 3 + 3 design (Storer, 1989)) and model-based designs (e.g., continual reassessment method (Oquigley et al., 1990)).

Aimed at speeding up the drug development and reducing the trial cost (van Rosmalen et al., 2018), pharmaceutical enterprises and regulatory agencies are willing to adopt innovative trial designs to leverage data from available supplemental information, for example, the historical data. Besides, the United States Food and Drug Administration (FDA) published guidance principles entitled “Interacting with the FDA on Complex Innovative Clinical Trial Designs for Drugs and Biological Products” in December 2020. This guidance document outlines the application cases and essential considerations pertaining to the utilization of information borrowing. The regulatory agency exhibits inclination towards embracing the incorporation of historical or supplemental data in clinical trials. Recently, numerous approaches have emerged to incorporate supplemental data, which involve power prior (Ibrahim and Chen, 2000), commensurate prior (Hobbs et al., 2012), meta-analytic predictive (MAP) prior (Schmidli et al., 2014), and multi-source exchangeability models (MEMs) (Kaizer et al., 2018). Power prior proposed by Ibrahim and Chen (Ibrahim and Chen, 2000) is constructed by discounting supplemental sources relative to the primary data (e.g., data derived from the current trial). Hobbs et al. introduced the commensurate prior (Hobbs et al., 2012) that utilizes precision parameters to gauge the similarity between the current and the historical trials. However, approaches mentioned above are often applicable in the cases where information is borrowed solely from one historical trial. The MAP and robust meta-analytic predictive (rMAP) method offers a viable solution for simultaneously borrowing information from multiple historical datasets (Schmidli et al., 2014).

In addition to constructing prior, multi-source exchangeability models (MEMs) have been extensively explored, since Kaizer et al. first illustrated that MEMs can be utilized to integrate multiple, potentially non-exchangeable, supplemental data sources into the analysis of primary data source while facilitate dynamic borrowing (Kaizer et al., 2018). Besides, the modeling framework of MEMs minimizes bias introduced from population drift that can occur across different historical trials through accounting for between-study heterogeneity. In this manuscript, we will introduce how the MEMs can be extended into the framework of Phase I clinical trials to identify the MTD of drug while further enhance the efficiency of early-phase clinical trials.

In recent years, late-onset toxicity has been widely reported (Alalawi et al., 2022) which will reduce the efficiency of trial since the patients’ toxicity endpoint cannot be quickly ascertained and outcome cannot be timely evaluated, specially for targeted therapy and immunotherapy. In the interest of providing timely treatment to patients enrolled next and reducing the risk of overdose, plenty of strategies have been proposed to handle the late-onset toxicity. The DA-CRM design (Liu et al., 2013) and TITE-BOIN design (Yuan et al., 2018) handle unobserved outcomes as missing data and utilize Bayesian Data Augmentation (BDA) techniques to predict and impute these missing values. Furthermore, Yuan et al. consider toxicity and efficacy as time-to-event variables and develop survival analysis models by treating unobserved outcomes as censoring events (Yuan and Yin, 2009). However, it is crucial to strike a balance between simplicity of trial and preference of statistical operating characteristics. TITE-CRM design (Cheung and Chappell, 2000), TITE-Keyboard design (Lin and Yuan, 2020) and CWL-U-BOIN design (Zhang and Zang, 2021) accommodate the challenge through the weighted likelihood approach, i.e., incorporating the observed total follow-up time for pending patients relative to the length of assessment window as weights in statistical models into approximation of weighted likelihood functions. The weighted likelihood approach stands out as a promising strategy offering Phase I clinical trials both notable statistical advantages and simplicity. More importantly, an increasing number of studies have shown that under limited toxicity information, borrowing historical information can further improve the statistical performance of dose exploration.

In this article, our approach is built upon the Keyboard design, which is one of the model-assisted designs. We apply MEMs for dynamic borrowing information from multiple supplemental data sources, including historical data, to inform the dose-escalation process. Furthermore, we extended our proposed method to handle late-onset toxicity using likelihood-based approach.

The remainder of this article proceeds as follows. We first introduce MEMs along with likelihood-based approach briefly, and further develop novel model-assisted designs in Section 2. We then examine the performance of the new design based on simulation studies and make extensive comparisons with existing methods in Section 3. We finally conclude with some remarks in Section 4.

## 2 Materials and methods

### 2.1 Keyboard design

Yan et al. (2017) proposed an intuitive and easily implementable model-assisted design for Phase I clinical trials known as the Keyboard design (Yan et al., 2017). This design utilizes the posterior distribution of toxicity probabilities to guide dose escalation and de-escalation. The Keyboard design starts by specifying a target toxicity rate and defines an appropriate dose interval $I^{*}=\left(\phi -\delta_{1},\phi +\delta_{2}\right)$ around this target rate ϕ , referred to as the “target key.” Subsequently, the interval [0,1] is divided into equally spaced “keys” on both sides of the target key, denoted as $I_{1},\ldots ,I_{M}$ . The ends of the [0,1] interval may not be covered by a key because their length is insufficient to form a complete key. The Keyboard design employs these keys to indicate the potential locations of the true toxicity rate for each dose level and guides the process of dose escalation and de-escalation.

Toxicity rate for each dose level is considered separately. Suppose in the current trial *C*, the toxicity rate at the *j*-th dose level is denoted as $p_{jc}$ , with a total of $n_{jc}$ patients enrolled at this dose level, *j = 1, … ,J*. At the decision time, only $Y_{jc}$ patients are observed to have experienced dose-limiting toxicity (DLT) events, while $Z_{jc}$ patients have not encountered such events, where $Z_{jc}=n_{jc}-Y_{jc}$ . We define $D_{jc}$ as the observed number of DLT events among the enrolled patients, i.e., $D_{jc}=\left(n_{jc},Y_{jc}\right)$ . In the Keyboard design, it is assumed that the number of DLT events, $Y_{jc}$ , follows a Beta-Binomial model, and a non-informative prior distribution $Beta\left(1,1\right)$ is assigned to the toxicity rate $p_{jc}$ for all dose levels. Consequently, the posterior distribution of $p_{jc}$ can be directly derived from the observed data.

$$p_{jc}|D_{jc}∼Beta\left(Y_{jc}+1,Z_{jc}+1\right)$$ (2.1)

And “the strongest key” is defined as the key with the maximum area under the posterior distribution curve of the DLT rate for the current dose

$$I_{\max}={\mathrm{argmax}}_{I_{1},\ldots ,I_{M}}\left\{\mathrm{Pr}\left(p_{jc}\in I_{m}|D_{jc}\right);m=1,\ldots ,M\right\}$$ (2.2)

The strongest key represents the most likely position of the true DLT rate for the current dose level. If the strongest key is positioned to the left (or right) of the target key, it signifies that the observed data suggests that patients may receive subtherapeutic doses (or over-therapeutic doses) at current dose level, thus necessitating a dose escalation (or de-escalation). Conversely, if the strongest key aligns with the target key, it indicates that the observed data supports the current dose level falling within an appropriate therapeutic range, thereby warranting its retention for the treatment of subsequent patients. By adhering to the aforementioned dose-escalation rules, the recruitment of subjects continues until the predefined maximum sample size *N* is exhausted. Ultimately, the dose level corresponding to the toxicity rate which is estimated closest to the desired target toxicity rate ϕ is identified as the maximum tolerated dose (MTD).

### 2.2 MEM-Keyboard design

When historical trial data, in which the same dose as in the current trial has been investigated, are available, the integration of the current trial *C* with a total of *H* independent historical trial datasets can be considered feasible for decision-making for corresponding dose levels. The MEMs framework, introduced by Kaizer et al., allows for dynamic borrowing of Supplemental Information (Kaizer et al., 2018). Herein we propose the MEM-Keyboard design, which offers the flexibility to incorporate one or multiple historical datasets for each dose when the doses have been investigated by one or multiple historical trials.

For each dose level, we employ independent modeling, following a methodology similar to that defined in Section 2.1. It is imperative to exercise caution when incorporating toxicity data from historical trials into the toxicity data of current dose, as a thorough evaluation of the similarity on toxicity rates between historical trials and current trial is required to ascertain the feasibility of information borrowing. More detailed discussion on this topic is provided in the discussion section (see Section 4). Specifically, we assume that the toxicity rate at the *j-*th dose level in each of the *H* independent historical trials is denoted as $p_{jh},h=1,\ldots ,H$ . In each historical trial, a total of $n_{jh}$ patients were enrolled at the *j-*th dose level, and the observed data reveal that $Y_{jh}$ patients experienced DLT events, while $Z_{jh}$ patients did not. The cumulative number of observed DLT events and the total number of enrolled patients from both the current and historical trials are denoted as $D_{j}$ .

For a total of *H* independent historical trials at the *j*-th dose level, there exist $K=2^{H}$ possible exchangeability models denoted as $\Omega_{jk},k=1,\ldots ,K$ . Each MEM considers one possible configuration of assumptions regarding exchangeability between the current trial *C* and a total of *H* independent historical trial. MEMs synthesize all possible exchangeability relationships between toxicity rates for each dose in current and historical trials, thereby inducing robustness to heterogeneity. To provide further clarification, if the equality $p_{jc}$ = $p_{jh}$ holds, it is considered that the toxicity rates of the drug at *j*-th dose level in trial *C* and the *h*-th historical trial are exchangeable for the *k*-th MEM (indicated by the function $s_{h,k}=1$ ). Conversely, if the equality does not hold, they are considered non-exchangeable ( $s_{h,k}=0$ ). Each MEM contains a set of indicators, ( ${S_{1}=s}_{1,k},\ldots ,{S_{H}=s}_{H,k}$ ).

Let *L* represent the likelihood function. For the *j*-th dose level, denote toxicity rates $p_{j}$ = $\left(p_{jc},p_{j1},...,p_{jH}\right)$ . Given the current exchangeability model $\Omega_{jk}$ , the integrated marginal likelihood for a specific MEM can be derivated as follows

$$p\left(D_{j}|\Omega_{jk}\right)=\int L\left(D_{j}|p_{j},\Omega_{jk}\right)\pi \left(p_{j}|\Omega_{jk}\right)dp_{j}=\frac{B\left(Y_{jc}+\alpha +\sum_{h=1}^{H}s_{h,k}Y_{jh},Z_{jc}+\beta +\sum_{r=1}^{H}s_{r,k}Z_{jr}\right)}{B\left(\alpha ,\beta \right)}\times \prod_{v=1}^{H}{\left(\frac{B\left(Y_{jv}+\alpha ,Z_{jv}+\beta \right)}{B\left(\alpha ,\beta \right)}\right)}^{{1-s}_{v,k}}$$ (2.3)

Where $L\left(D_{j}|p_{j},\Omega_{jk}\right)\propto {p_{j}}^{Y_{j}}{\left(1-p_{j}\right)}^{Z_{j}}$ , $Y_{j}$ = $\left(Y_{jc},Y_{j1},...,Y_{jH}\right)$ , $Z_{j}$ = $\left(Z_{jc},Z_{j1},...,Z_{jH}\right)$ , $B\left(c,d\right)=\frac{\Gamma \left(c\right)\Gamma \left(d\right)}{\Gamma \left(c+d\right)}$ . For different historical trial data, different $B\left(c,d\right)$ can be specified. In this study, the non-informative prior $Beta\left(1,1\right)$ is adopted.

The posterior model weights for each MEM are given by

$$\omega_{k}=p\left(\Omega_{jk}|D_{j}\right)=\frac{p\left(D_{j}|\Omega_{jk}\right)\pi \left(\Omega_{jk}\right)}{\sum_{l=1}^{K}p\left(D_{j}|\Omega_{jl}\right)\pi \left(\Omega_{jl}\right)}$$ (2.4)

where $\pi \left(\Omega_{jk}\right)$ is the prior probability that $\Omega_{jk}$ is the true model, $\sum_{k=1}^{K}\omega_{k}=1$ . To reduce the dimension of the prior space, MEMs specify the $\pi \left(\Omega_{jk}\right)$ on the independent supplemental sources rather than models, $\pi \left(\Omega_{jk}\right)=\pi \left(S_{1}=s_{1,k},\ldots ,S_{H}=s_{H,k}\right)=\pi \left(S_{1}=s_{1,k}\right)\times \ldots \times \pi \left(S_{H}=s_{H,k}\right)$ . This results in drastic dimension reduction in that it necessitates the specification of only *H* source-specific prior inclusion probabilities in place of $2^{H}$ prior model probabilities comprising the entire model space. Posterior model weights depend on the specification of priors in the model (Zabor et al., 2022) and we will discuss this issue in Section 3.3.

Therefore, the marginal posterior distribution of $p_{jc}$ is the weighted average of the posterior models from the *K* MEM and can be expressed as follows

$$p\left(p_{jc}|D_{j}\right)=\sum_{k=1}^{K}\omega_{k}Beta\left(Y_{jc}+\alpha +\sum_{h=1}^{H}s_{h,k}Y_{jh},Z_{jc}+\beta +\sum_{r=1}^{H}s_{r,k}Z_{jr}\right)$$ (2.5)

The strongest key is defined as

$$I_{\max}={\mathrm{argmax}}_{I_{1},\ldots ,I_{M}}\left\{\sum_{k=1}^{K}\omega_{k}\mathrm{Pr}\left(p_{jc}\in I_{m}|{\Omega_{jk},D}_{j}\right);m=1,\ldots ,M\right\}$$ (2.6)

Subsequently, dose escalation and de-escalation will be conducted according to the rules of the Keyboard design described in Section 2.1 to identify the maximum tolerated dose (MTD). Besides, our method can simply be extended to drug-drug combination trials when historical trials involve the same combinations of drugs by utilizing the dose escalation and de-escalation rules for Keyboard combination design (Pan et al., 2020). Similar to the Keyboard design, the introduced MEM-Keyboard design incorporates the available historical trial information and employs an exhaustive examination of all possibilities that could arise and calculating the posterior distributions of $p_{jc}$ for each of these potential cases at the current dose level. A decision table (see Table 1, Supplementary Tables S1, S2) can be tabulated before the onset of a trial. Based on different historical trial information at the current dose level, different dose escalation and de-escalation rules are applied for different dose levels. When there is no available historical information [e.g., when the observed ratio of patients who experienced DLT to the total number of treated patients is (0/0)], the MEM-Keyboard design reverts to the Keyboard design. Physicians or sponsors can make dose escalation or de-escalation decisions based on the decision table.

**TABLE 1:** Dose-escalation and de-escalation boundaries for MEM-Keyboard with a target DLT rate of 0.28 and cohort size of 3 up to 12 patients (historical DLT rate for current dose level is 1/7, 1/5, and 1/6).

Number of patients treated	Escalate if number of DLT <=	De-escalate if number of DLT >=	Eliminate if number of DLT >=
1	0	1	NA
2	0	2	2
3	0	2	3
4	1	2	3
5	1	3	4
6	1	3	4
7	1	3	4
8	2	4	5
9	2	4	5
10	2	4	6
11	2	5	6
12	2	5	6

When the historical data is given as follows: 1/7, 1/5, and 1/6 (let y/n represents the observed ratio of patients who experienced DLT to the total number of treated patients, namely the DLT rate). Assuming a target DLT rate of 0.28 and cohort size of 3 up to 12 patients, Table 1 shows the decision tables for MEM-Keyboard. It can be concluded that when the historical data indicates a lower toxicity level for current dose, MEM-Keyboard is more inclined to make decisions for dose escalation or dose maintenance.

### 2.3 MEM-TITE-Keyboard design

#### 2.3.1 Likelihood with pending DLT data

Many early-phase clinical trials typically require the timely observation of toxicity outcomes among enrolled participants. However, late-onset toxicity commonly exists in various clinical scenarios, particularly in the era of novel treatment strategies such as targeted therapies and immunotherapies. Under late-onset toxicity scenarios, as the dose-toxicity information available at decision time is limited, we further consider borrowing historical information via the MEM approach to improve the efficiency of dose-finding trials with delayed toxicity issue. In this study, we further expand the MEM-Keyboard design to the MEM-TITE-Keyboard design. This extension is motivated by the desire to expedite drug development processes and enhance the overall efficiency of clinical trials. The present research incorporates a likelihood approximation method proposed by Lin and Yuan (Lin and Yuan, 2020) to address the challenge of handling pending toxicity data resulted from either rapid participant enrollment or late-onset toxicity manifestation.

Let *N* represent the total number of patients sequentially enrolled in the study, with each patient undergoing a fixed follow-up duration of *T*. Let $x_{i}$ denote a binary indicator that represents whether the *i*-th patient experienced a DLT event within the follow-up period (0, *T*). If patient’s DLT event occurred within the follow-up period, $x_{i}=1$ ; otherwise, $x_{i}=0$ . Moreover, $t_{i}$ denotes the time from drug administration to the onset of DLT for each patient, where $0\leq t_{i}\leq T$ . Additionally, $\delta_{i}$ signifies the availability of toxicity information at the decision time κ, with $\delta_{i}=1$ indicating a confirmed outcome and $\delta_{i}=0$ representing an outcome yet to be determined. In the latter case, the patient has been followed up for a duration of $u_{i}$ . If no DLT event is observed for a patient at the decision time κ, $X_{i}=0$ ; otherwise, $X_{i}=1$ . It is evident that $X_{i}=1$ implies $x_{i}=1$ .

Assuming there are *J* dose levels in the current trial, the occurrence of DLT events is observed for $Y_{jc}$ out of $n_{j}$ patients at the *j-*th dose level ( j=1,…,J ) at the decision time κ, where $Y_{jc}=\sum_{i=1}^{n_{j}}\delta_{i}X_{i}$ . Here, the toxicity outcome is known for *i*-th patient ( $X_{i}{=x}_{i}$ ) if $\delta_{i}=1$ . While for patients with $\delta_{i}=0$ , the toxicity outcome is uncertain at decision time κ, there are two possibilities: 1) the patient will not experience a DLT event, or 2) the patient will experience a DLT event, but it has not been observed yet. Assuming that the observed data at decision time κ for the *j*-th dose level is denoted as $D_{\mathrm{jc}}^{\prime }=\left(X_{1},...,X_{n_{j}},\delta_{1},...,\delta_{n_{j}}\right)$ , the joint likelihood can be derived through incorporating the observed total follow-up time into assessment as follows:

$$L\left(p_{j}|D_{jc}^{\prime }\right)\propto \prod_{i=1}^{n_{j}}{p_{j}}^{{\delta_{i}x}_{i}}{\left(1-p_{j}\right)}^{\delta_{i}\left(1-x_{i}\right)}{\left(1-p_{j}m_{i}\right)}^{1-\delta_{i}}={p_{j}}^{Y_{jc}}{\left(1-p_{j}\right)}^{z_{jc}}\prod_{i=1}^{n_{j}}{\left(1-p_{j}m_{i}\right)}^{1-\delta_{i}}$$ (2.7)

where $m_{i}=\mathrm{Pr}\left(t_{i}\leq u_{i}|x_{i}=1\right)$ denotes the weight that is assigned manually, and $z_{jc}=\sum_{i=1}^{n_{j}}\delta_{i}\left(1-X_{i}\right)$ represents the number of patients who have completed the assessment at decision time κ and have not experienced any DLT events. For detailed derivations, see Lin and Yuan (2020).

To ensure the simplicity of the Keyboard design and enable the dose escalation/de-escalation rules to be pre-specified before the trial initiation, Lin and Yuan approximated the last term in the equation above as follows

$${\left(1-p_{j}m_{i}\right)}^{1-\delta_{i}}\approx {\left(1-p_{j}\right)}^{m_{i}\left(1-\delta_{i}\right)}$$ (2.8)

As a result, the likelihood can be approximated as follows

$$L\left(p_{j}|D_{jc}^{\prime }\right)\propto {p_{j}}^{Y_{jc}}{\left(1-p_{j}\right)}^{Z_{je}}$$ (2.9)

where $Z_{je}=z_{jc}+\sum_{i=1}^{n_{j}}m_{i}\left(1-\delta_{i}\right)$ is the ‘effective’ number of patients who have not experienced DLT, with “e” for “effective” to distinguish the utilization between $Z_{je}$ and $Z_{jc}$ aforementioned. Lin and Yuan have demonstrated that this approximation is an accurate approximation of the original likelihood (Lin and Yuan, 2020). Though multiple weighting schemes can be employed, here we specify the weight $m_{i}$ in a uniform scheme (Cheung and Chappell, 2000) by assuming the time-to-toxicity outcome is uniformly distributed over the assessment period (0, *T*). Consequently, $m_{i}$ can be interpreted as the follow-up proportion that the *i*-th patient has finished.

Moreover, to further present the decision table in a straightforward and intuitive manner, a “effective” sample size (ESS) is adopted. At the *j*-th dose level, ESS _*j*_ = No. of non-pending patients at the *j*-th dose level + $\frac{\text{Total}\;\text{follow}-\text{up}\;\text{time}\;\text{for}\;\text{pending}\;\text{patients}\;\text{at}\;\text{the}\;\mathrm{j-th}\;\text{dose}\;\text{level}}{\text{Length}\;\text{of}\;\text{assessment}\;\text{window}}$ .

#### 2.3.2 MEM-TITE-Keyboard design

Of note, in the context of late-onset toxicity, the DLT assessment windows may vary across different Phase I trials. In this study, the following approach is adopted. When the follow-up time $\tau_{i},i=1,\ldots ,H$ , in the historical trials differs from the follow-up time *T* in the current trial, the following considerations are made. If $\tau_{i}<T$ , the outcomes of patients in the historical data $D_{jh}$ who have not experienced DLT are treated as pending toxicity outcomes, awaiting determination. Consequently, the toxicity outcomes of patients in the historical data can be categorized into two classes: 1) outcomes pending determination, where patients have $X_{i}=x_{i}=0,\delta_{i}=0$ ; 2) outcomes already determined, where patients have $X_{i}=x_{i}=1,\delta_{i}=1$ .

For the *h*-th historical trial ( h=1,…,H ), the likelihood is given by

$$L\left(p_{jh}|D_{jh}\right)\propto \prod_{i=1}^{n_{j}}{p_{jh}}^{{\delta_{i}x}_{i}}{\left(1-p_{jh}m_{h}\right)}^{1-\delta_{i}}={p_{jh}}^{Y_{jh}}{\left(1-p_{jh}m_{h}\right)}^{Z_{jh}}$$ (2.10)

where $m_{h}=\frac{\tau_{i}}{T}$ . If $\tau_{i}>T$ , then let $m_{h}=1$ to overcome the possible over-estimation of toxicity. Based on the observed data $D_{j}^{\prime }$ , for a specific MEM at the *j*-th dose level, the integrated marginal likelihood can be written as

$$\begin{array}{c} p\left(D_{j}^{\prime }|\Omega_{jk}\right)\propto \int {p_{jc}}^{Y_{jc}+\sum_{h=1}^{H}s_{h,k}Y_{jh}}{\left(1-p_{jc}\right)}^{z_{jc}}\prod_{i=1}^{n_{j}}{\left(1-p_{jc}m_{i}\right)}^{1-\delta_{i}}\times \prod_{r=1}^{H}{\left(1-p_{jc}m_{r}\right)}^{s_{r,k}Z_{jr}}\\ B\left(p_{jc}\left|\alpha \right.,\beta \right)dp_{jc}\times \int {\left\{{p_{jv}}^{Y_{jv}}{\left(1-p_{jv}m_{v}\right)}^{Z_{jv}}\right\}}^{{1-s}_{v,k}}B\left(p_{jv}|\alpha ,\beta \right)dp_{jv},v=1,\ldots ,H \end{array}$$ (2.11)

The marginal posterior distribution of $p_{jc}$ can be further expressed as a weighted average that includes all possible exchangeable relationships

$$p\left(p_{jc}|D_{j}\right)\propto \sum_{k=1}^{K}\omega_{k}\left\{{p_{jc}}^{Y_{jc}+\alpha +\sum_{h=1}^{H}s_{h,k}Y_{jh}}{\left(1-p_{jc}\right)}^{z_{jc}+\beta }\times \prod_{i=1}^{n_{j}}{\left(1-p_{jc}m_{i}\right)}^{1-\delta_{i}}\prod_{r=1}^{H}{\left(1-p_{jc}m_{r}\right)}^{s_{r,k}Z_{jr}}\right\}$$ (2.12)

We refer to the design proposed above as the MEM-TITE-Keyboard (E) design for it utilizes the exact likelihood function.

To ensure the simplicity of the design, a similar approximation (see 2.9) is employed to approximate the joint likelihood as follows

$$L\left(D_{j}|p_{jc},\Omega_{jk}\right)\propto {p_{jc}}^{Y_{jc}+\sum_{h=1}^{H}s_{h,k}Y_{jh}}{\left(1-p_{jc}\right)}^{Z_{je}+\sum_{r=1}^{H}s_{r,k}m_{r}Z_{jr}}\times \prod_{v=1}^{H}{\left\{{p_{jv}}^{Y_{jv}}{\left(1-p_{jv}\right)}^{m_{v}Z_{jv}}\right\}}^{{1-s}_{v,k}}$$ (2.13)

The integrated marginal likelihood, denoted as $p\left(D_{j}|\Omega_{jk}\right)$ , for a specific MEM at the *j*-th dose level can be further given by

$$p\left(D_{j}|\Omega_{jk}\right)\propto \frac{B\left(Y_{jc}+\alpha +\sum_{h=1}^{H}s_{h,k}Y_{jh},Z_{je}+\beta +\sum_{r=1}^{H}s_{r,k}m_{r}Z_{jr}\right)}{B\left(\alpha ,\beta \right)}\times \prod_{v=1}^{H}{\left(\frac{B\left(Y_{jv}+\alpha ,m_{v}Z_{jv}+\beta \right)}{B\left(\alpha ,\beta \right)}\right)}^{{1-s}_{v,k}}$$ (2.14)

Thus the marginal posterior distribution of $p_{jc}$ can be expressed as

$$p\left(p_{jc}|D_{j}\right)\propto \sum_{k=1}^{K}\omega_{k}Beta\left(Y_{jc}+\alpha +\sum_{h=1}^{H}s_{h,k}Y_{jh},Z_{je}+\beta +\sum_{r=1}^{H}s_{r,k}m_{r}Z_{jr}\right)$$ (2.15)

**FIGURE 1:**
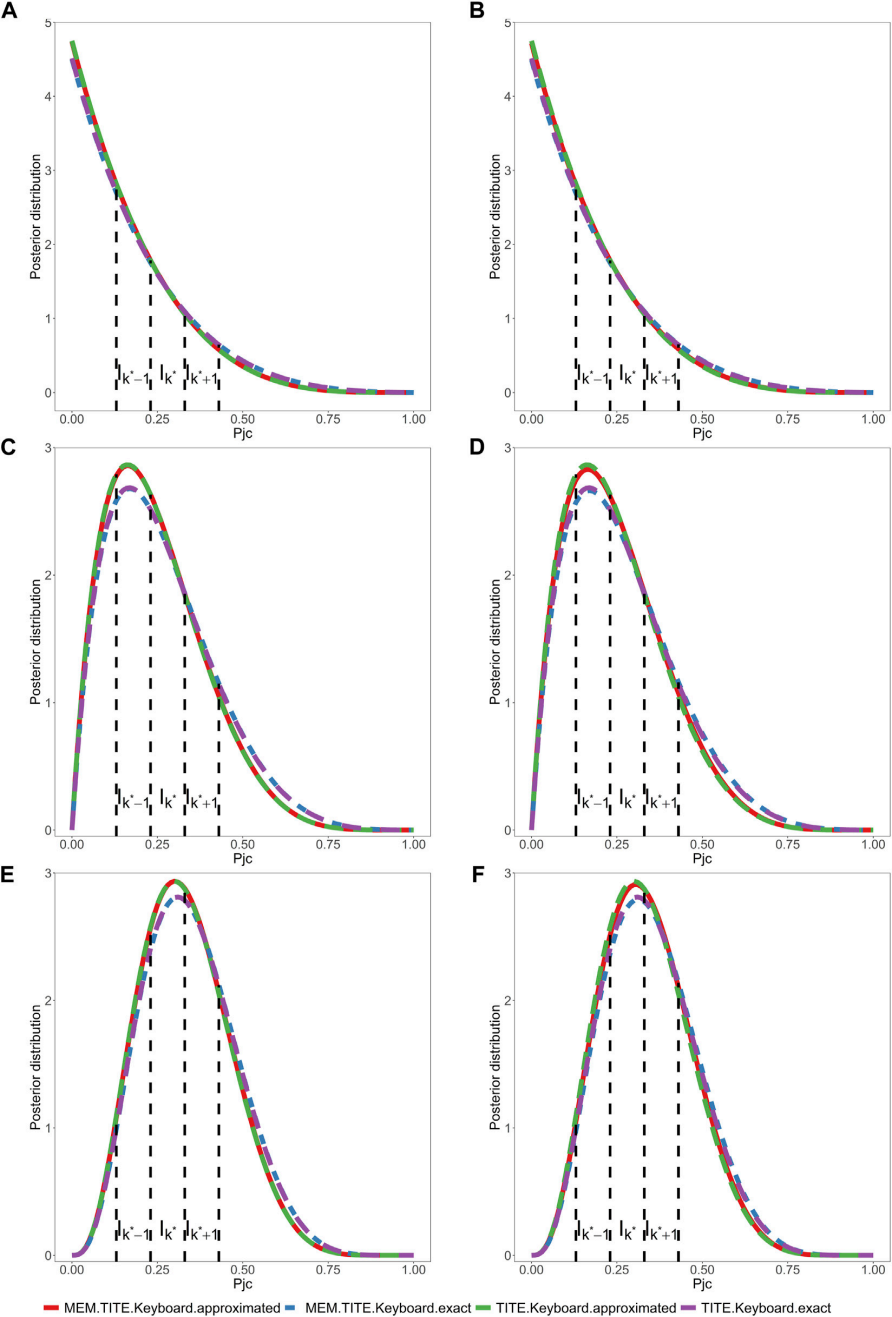
Assuming a toxicity rate of 0.3 at the current dose level, and considering two different settings for the six historical trials. One is that, in all three historical trials, 7 subjects were enrolled, and 3 subjects experienced DLT events. The other is that, in all three historical trials, 6 subjects were enrolled, and 2 subjects experienced DLT events. Under these two different historical trial settings, we will examine the following three scenarios. (1) **(A, B)** depicts the posterior distribution of DLT rate, where 6 subjects are enrolled, with 0 subjects experiencing DLT events. One subject’s outcome has been determined as no DLT event, and the remaining 5 subjects have weights $m_{i}$ of 0.25, 0.4, 0.55, 0.7, and 0.85, respectively. (2) **(C, D)** depicts the posterior distribution of DLT rate, 9 subjects are enrolled, with 1 subject experiencing a DLT event. Three subjects’ outcomes have been determined as no DLT events, and the remaining 6 subjects have weights $m_{i}$ of 0.15, 0.3, 0.45, 0.6, 0.75, and 0.9, respectively. (3) **(E, F)** depicts the posterior distribution of DLT rate, 12 subjects are enrolled, with 3 subjects experiencing DLT events. Eight subjects’ outcomes have been determined as no DLT events, and the remaining 4 subjects have weights $m_{i}$ of 0.2, 0.4, 0.6, and 0.8, respectively.

The design proposed above is referred to as MEM-TITE-Keyboard design for its employment of approximate likelihood. As shown by three data examples provided in Figure 1, this approximation still maintains a high level of accuracy.

Similar to the methodology described in Section 2.2, the MEM-TITE-Keyboard approach utilizes the strongest key to guide the process of dose escalation and de-escalation. And a decision table can be pre-tabulated (examples provided in Table 2, Supplementary Tables S3, S4) to simplify trial conducted in practice. When there is no pending DLT data, the MEM-TITE-Keyboard design reverts to the MEM-Keyboard design in a seamless way. Considering the safety for patients, we require that dose escalation is not allowed until at least two patients have completed the DLT assessment at the current dose level $j^{\mathrm{curr}}$ .

**TABLE 2:** Dose-escalation and de-escalation boundaries for MEM-TITE-Keyboard with a target DLT rate of 0.28 and cohort size of 3 up to 12 patients (historical DLT rate for current dose level is 1/7, 1/5, and 1/6).

Number of patients treated	Number of observed DLTs	Number of pending patients	Escalation	Stay	De-escalation
3	0	< 2	Yes		
3	0	> 1		Suspend	
3	1	0		Yes	
3	1	1 ∼ 2		ESS>= 2.47	ESS< 2.47
3	2	< 2			Yes
3	3	0			Yes & Eliminate
6	0	< 6	Yes		
6	0	6	ESS> 0.01	ESS<= 0.01	
6	1	< 2	Yes		
6	1	2 ∼ 3	ESS> 4.46	ESS<= 4.46	
6	1	4 ∼ 5	ESS> 4.46	ESS in [2.47,4.46]	ESS< 2.47
6	2	0		Yes	
6	2	1 ∼ 4		ESS>= 5.52	ESS< 5.52
6	3	< 4			Yes
6	> 3	< 3			Yes & Eliminate
9	0	< 9	Yes		
9	0	9	ESS> 0.01	ESS<= 0.01	
9	1	< 5	Yes		
9	1	5 ∼ 6	ESS> 4.46	ESS<= 4.46	
9	1	7 ∼ 8	ESS> 4.46	ESS in [2.47,4.46]	ESS< 2.47
9	2	0	Yes		
9	2	1 ∼ 3	ESS> 8.91	ESS<= 8.91	
9	2	4 ∼ 7	ESS> 8.91	ESS in [5.52,8.91]	ESS< 5.52
9	3	0		Yes	
9	3	1 ∼ 6		ESS>= 8.57	ESS< 8.57
9	4	< 6			Yes
9	> 4	< 5			Yes & Eliminate
12	0	< 12	Yes		
12	0	12	ESS> 0.01	ESS<= 0.01	
12	1	< 8	Yes		
12	1	8 ∼ 9	ESS> 4.46	ESS<= 4.46	
12	1	10 ∼ 11	ESS> 4.46	ESS in [2.47,4.46]	ESS< 2.47
12	2	< 4	Yes		
12	2	4 ∼ 6	ESS> 8.91	ESS<= 8.91	
12	2	7 ∼ 10	ESS> 8.91	ESS in [5.52,8.91]	ESS< 5.52
12	3	< 4		Yes	
12	3	4 ∼ 9		ESS>= 8.57	ESS< 8.57
12	4	0		Yes	
12	4	1 ∼ 8		ESS>= 11.63	ESS< 11.63
12	5	< 8			Yes
12	> 5	< 7			Yes & Eliminate

ESS _*j*_ = No. of non-pending patients at the *j*-th dose level + $\frac{\text{Total}\;\text{follow}-\text{up}\;\text{time}\;\text{for}\;\text{pending}\;\text{patients}\;\text{at}\;\text{the}\;\mathrm{j-th}\;\text{dose}\;\text{level}}{\text{Length}\;\text{of}\;\text{assessment}\;\text{window}}$ . From Table 2, the same conclusion as that of Table 1 can be drawn that MEM-TITE-Keyboard is more inclined to make decisions for dose escalation or dose maintenance when the historical data indicates a lower toxicity level for current dose.

### 2.4 Trial design

The dose-finding rules for MEM-Keyboard and MEM-TITE-Keyboard based on the probability model described in Section 2.2 and Section 2.3 are presented as follows:

1. The first cohort of patients are treated at the lowest dose level or another physician-specified dose level.
2. Count the total number of patients enrolled in the current trial and the number of patients who have experienced DLT. For MEM-TITE-Keyboard, an additional calculation is required for the ESS.
3. Update the posterior distribution of $p_{jc}$ (see 2.5 and 2.15) and identify the “strongest key”. Compare the “strongest key” with “target key”,
  a. If the strongest key is on the left of the target key, escalate the dose level to $j^{\mathrm{curr}}+1$ .
  b. If the strongest key is the target key, retain the dose level.
  c. If the strongest key is on the right of the target key, de-escalate the dose level to $j^{\mathrm{curr}}-1$ .
4. Repeat the steps 1-3 until the predefined maximum sample size *N* is exhausted.
5. Once the trial ends, the dose level with the smallest difference $\left|{\tilde{p}}_{jc}-\phi \right|$ is identified as MTD, where the ${\tilde{p}}_{jc}$ is the isotonically transformed posterior mean of $p_{jc}$ obtained by applying the same pooled adjacent violators algorithm as that in the Keyboard design (Yan et al., 2017).

Additionally, a dose elimination rule is adopted if any dose satisfies the conditional probability $\mathrm{Pr}\left(p_{jc}>\phi |D_{jc}\right)>\xi$ when $n_{jc}\geq 3$ then that dose and any higher doses shall be eliminated during each dose escalation decision, where *ξ* is the prespecified elimination cutoff, say 0.95. If the lowest dose level is eliminated, the trial should be early terminated.

## 3 Results

### 3.1 Simulation settings

This section presents an evaluation of the operating characteristics of MEM-Keyboard and MEM-TITE-Keyboard in scenarios where the occurrence of DLT exists heterogeneity in historical trials. The study assumes a total of four dose levels under investigation, with a predefined target DLT rate of 0.28. Each cohort comprises three patients, allowing for a maximum of eight cohorts and a total sample size of 24. In this design, the width of the keyboard is set to 0.1. As for the prior specification, a non-informative Beta (1,1) prior is employed. The trial is designed to terminate if the number of treated patients reaches nine at any dose level and a decision is made to maintain the dose.

To account for potential variations in evaluation window durations across different trials in the context of late-onset toxicity, the current trial has a follow-up duration of 3 months, while three historical trials are simulated with total follow-up durations of 1 month, 2 months, and 3 months, respectively. The average enrollment rate is set at 2 patients per month. Table 3 presents a comprehensive overview of 13 simulation scenarios. Scenarios 1-4 consider the situation where the MTD resides at different dose levels, while the DLT rate data in the historical trials aligns with the true DLT rate. Scenarios 5-7 introduce heterogeneity across trials, with varying numbers of exchangeable data sources at different dose levels (2/1/0). Scenarios 8-9 explore cases where the DLT rate in historical trials deviates from the true DLT rate. Lastly, scenarios 10–13 encompass situations where both inconsistencies in DLT rate from historical trials and inter-trial heterogeneity exist. Considering the small sample size of cohort and the potential heterogeneity among current and historical trials, we opted to set the prior exchangeability probability $\pi \left(S_{h}=s_{h,k}\right)$ to the probability of 0.1 recommended by Zabor et al. (2022).

**TABLE 3:** Simulation settings.

	dose level					dose level			
	1	2	3	4		1	2	3	4
Scenario 1					Scenario 4				
p.true	**0.28**	0.41	0.52	0.63	p.true	0.03	0.11	0.14	**0.28**
p.historical	**0.33**	0.43	0.50	0.66	p.historical	0.00	0.13	0.17	**0.33**
data.historical	2/6	3/7	2/4	0/0	data.historical	0/3	1/8	1/6	2/6
	2/6	3/7	2/4	0/0		0/3	1/8	1/6	2/6
	2/6	3/7	2/4	0/0		0/3	1/8	1/6	2/6
Scenario 2					Scenario 5				
p.true	0.14	**0.28**	0.41	0.52	p.true	0.14	**0.28**	0.41	0.52
p.historical	0.17	**0.33**	0.43	0.50	p.historical	0.16	**0.32**	0.43	0.50
data.historical	1/6	2/6	3/7	2/4	data.historical	1/7	2/7	3/8	1/2
	1/6	2/6	3/7	2/4		1/6	2/6	3/7	2/4
	1/6	2/6	3/7	2/4		1/6	2/6	3/7	2/4
Scenario 3					Scenario 6				
p.true	0.11	0.14	**0.28**	0.41	p.true	0.14	**0.28**	0.41	0.52
p.historical	0.13	0.17	**0.33**	0.43	p.historical	0.17	**0.33**	0.43	0.50
data.historical	1/8	1/6	2/6	3/7	data.historical	1/7	2/7	3/8	1/2
	1/8	1/6	2/6	3/7		1/5	2/5	3/5	0/0
	1/8	1/6	2/6	3/7		1/6	2/6	3/7	2/4
Scenario 7					Scenario 10				
p.true	0.14	**0.28**	0.41	0.52	p.true	0.11	0.14	**0.28**	0.41
p.historical	0.17	**0.33**	0.43	0.50	p.historical	0.16	**0.32**	0.43	0.55
data.historical	1/8	2/7	3/8	1/2	data.historical	1/7	2/7	3/8	1/2
	1/5	3/7	4/8	0/0		1/6	2/6	3/7	2/4
	1/5	2/7	2/5	1/2		1/6	2/6	3/7	2/4
Scenario 8					Scenario 11				
p.true	0.11	0.14	**0.28**	0.41	p.true	0.11	0.14	**0.28**	0.41
p.historical	0.17	**0.33**	0.43	0.50	p.historical	0.17	**0.33**	0.43	0.50
data.historical	1/6	2/6	3/7	2/4	data.historical	1/7	2/7	3/8	1/2
	1/6	2/6	3/7	2/4		1/5	2/5	3/5	0/0
	1/6	2/6	3/7	2/4		1/6	2/6	3/7	2/4
Scenario 9					Scenario 12				
p.true	0.11	0.14	**0.28**	0.41	p.true	0.11	0.14	**0.28**	0.41
p.historical	0.00	0.13	0.17	**0.33**	p.historical	0.00	0.13	0.16	**0.32**
data.historical	0/3	1/8	1/6	2/6	data.historical	0/4	1/7	1/7	2/7
	0/3	1/8	1/6	2/6		0/3	1/8	1/6	2/6
	0/3	1/8	1/6	2/6		0/3	1/8	1/6	2/6
Scenario 13									
p.true	0.11	0.14	**0.28**	0.41					
p.historical	0.00	0.13	0.17	**0.33**					
data.historical	0/4	1/6	1/7	2/7					
	0/4	1/9	1/5	2/5					
	0/4	1/8	1/6	2/6					

The MTD is in boldface. p.true represents the DLT rates for the current clinical trial. p.historical represents the average DLT rates obtained from historical trial data. data.historical are data available from historical trials (y/n represents the observed ratio of patients who experienced DLT to the total number of treated patients, namely the DLT rate).

The methods used for comparison in this study include the 3 + 3 design, Keyboard design, MEM-Keyboard design, TITE-Keyboard design, TITE-Keyboard (E) design, MEM-TITE-Keyboard design, and MEM-TITE-Keyboard (E) design. The TITE-Keyboard design and TITE-Keyboard (E) design utilize the approximated and exact likelihood function respectively as in Lin and Yuan (2020). In 3 + 3 design, Keyboard design and the MEM-Keyboard design, dose escalation decisions are made only after completion of DLT assessment for all enrolled patients.

To collectively provide a thorough assessment, multiple criteria are employed to conduct a comprehensive evaluation of the statistical performance, thereby facilitating our evaluation of the accuracy, safety, and reliability of the proposed design. The performance measures used for statistical evaluation encompass the following criteria: (1): Selection (%): the percentage of simulated trials that each dose level is identified as MTD. (2) pts at MTD: the average numbers of patients allocated to each dose level. (3) Stop (%): indicating early stop percentage. In the 3 + 3 design, stop is defined as the occurrence of 2 or more DLT events after enrolling the first 3 patients. In the Keyboard, TITE-Keyboard, MEM-Keyboard and MEM-TITE-Keyboard designs, stop is defined as the elimination of the first dose level based on the criterion $\mathrm{Pr}\left(p_{jc}>\phi |D_{jc}\right)>0.95$ . (4) Overdose (%): the risk of overdosing, which is the percentage of simulated trials that treat equal to or more than 60% of the patients at doses above the MTD. (5) Risk of poor allocation (%): the percentage of simulated trials allocating fewer than 6 patients to the MTD (maximum sample size divided by the number of dose levels). (6) Duration: the average duration required to complete the trial. (7) Sample size: the average quantity of the total number of patients required for each trial over simulated trials.

### 3.2 Simulation results

**FIGURE 2:**
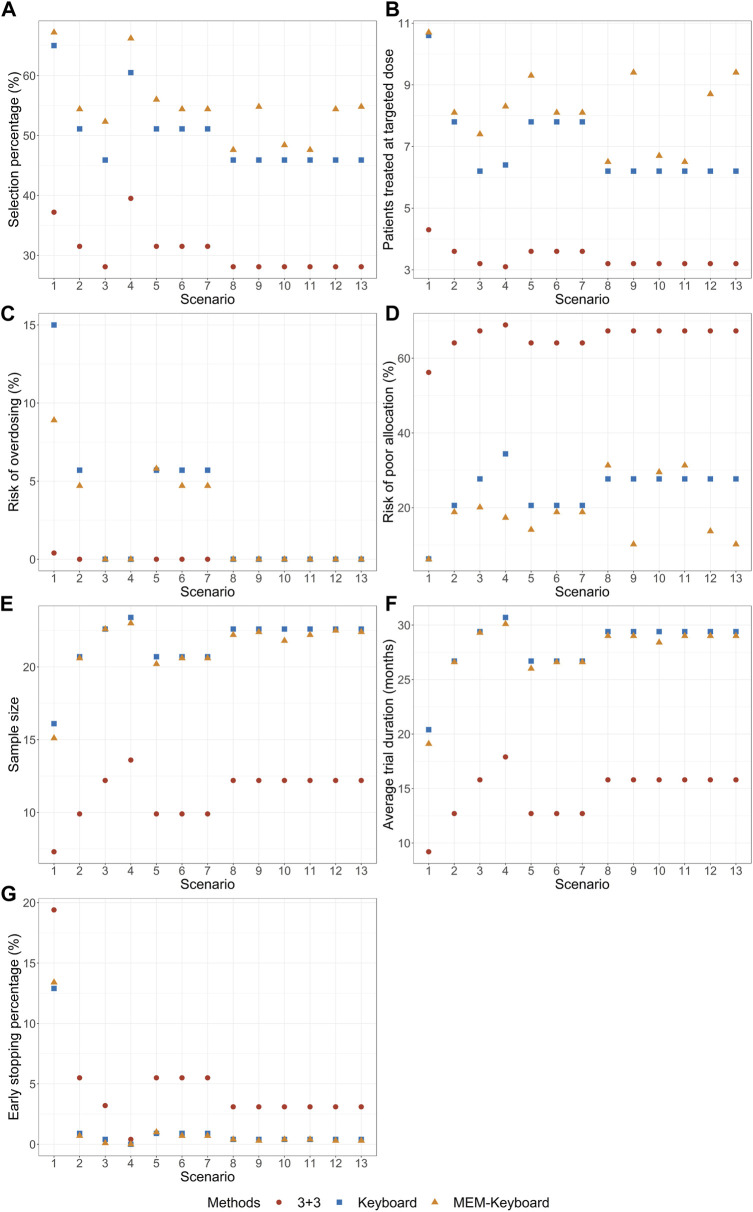
Simulation results with sample size of 24 and cohort size of 3 without considering late-onset toxicity. 3 + 3 is the conventional 3 + 3 design; Keyboard is the Keyboard Q19 design; MEM-Keyboard is the proposed design that incorporates the historical data. **(A)** depicts the percentage of simulated trials that MTD is correctly selected. **(B)** depicts the average numbers of patients allocated to MTD. **(C)** depicts the risk of overdosing. **(D)** depicts the risk of poor allocation. **(E)** depicts the average quantity of the total number of patients required for each trial over simulated trials. **(F)** depicts the average duration required to complete the trial. **(G)** depicts the early stop percentage.

**FIGURE 3:**
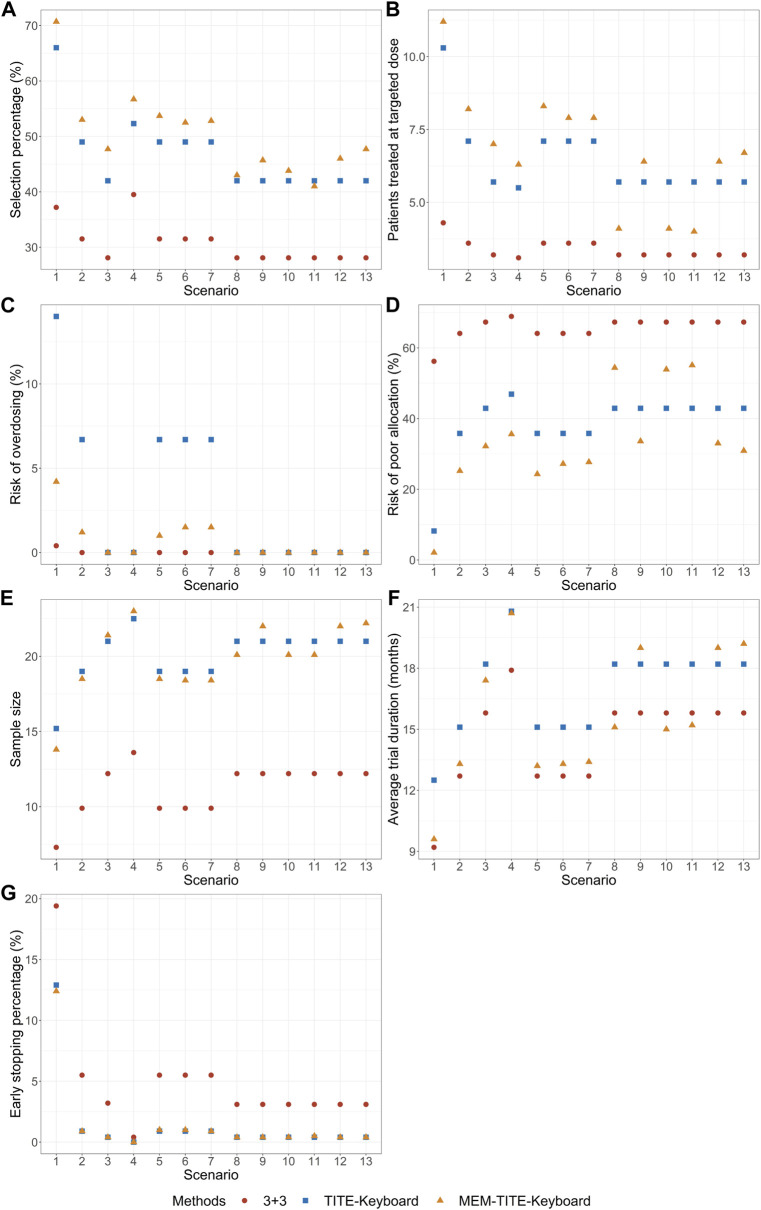
Simulation results with sample size of 24 and cohort size of 3 considering late-onset toxicity. TITE-Keyboard is the time-to-event keyboard designs that utilizes the approximated function; MEM-TITE-Keyboard is the proposed design that utilizes the approximated function. **(A)** depicts the percentage of simulated trials that MTD is correctly selected. **(B)** depicts the average numbers of patients allocated to MTD. **(C)** depicts the risk of overdosing. **(D)** depicts the risk of poor allocation. **(E)** depicts the average quantity of the total number of patients required for each trial over simulated trials. **(F)** depicts the average duration required to complete the trial. **(G)** depicts the early stop percentage.

The results of the simulation study are presented in Figures 2, 3 demonstrate that when the DLT rate data from historical trials are consistent with the true DLT rate, both the MEM-Keyboard design and the MEM-TITE-Keyboard design exhibit improved operating characteristics compared to their counterparts, the Keyboard design and the TITE-Keyboard design, respectively, across various scenarios (Scenario 1 to Scenario 4). In contrast, the conventional 3 + 3 design, due to its overly conservation, displays the lowest selection rate and the highest risk of poor allocation in all scenarios. Notably, when the MTD is not at the highest dose level, the MEM-TITE-Keyboard design offers additional benefits beyond an enhanced selection rate and reduced risks of overdosing and poor allocation (see Scenario 2). Specifically, by leveraging historical information, the MEM-TITE-Keyboard design facilitates a more efficient patient allocation process, enabling patients to be allocated more directly to the appropriate dose for treatment, rather than undergoing multiple sequential assignments to neighboring doses to observe DLT events (e.g., more patients are allocated to the MTD in Scenario 3). Consequently, this design significantly reduces the time required to monitor patients’ toxic outcomes and increases the likelihood of meeting the stopping criteria for terminating the trial, i.e., reaching a predefined number of patients (i.e., 9) receiving treatment at a specific dose while still making the decision to maintain the dose. Importantly, when the historical DLT rates align with the current rates and heterogeneity exists among historical trials, wherein the number of exchangeable data sources decreases at different dose levels (Scenario 5 to Scenario 7), the performance of the proposed designs remains robust and comparable to that observed in scenarios without heterogeneity among historical trials.

When there is a disparity between the historical DLT rates and the current DLT rates, both the MEM-Keyboard and MEM-TITE-Keyboard designs exhibit a sustained high selection rate. In situations where the historical data indicates intolerable toxicity for patients at a particular dose level, the MEM-Keyboard and MEM-TITE-Keyboard tend to allocate a larger number of subjects to lower doses, consequently elevating the risk of poor allocation (see Scenario 8). Conversely, when the historical data suggests a lower toxicity profile and supports dose escalation, both the MEM-Keyboard and MEM-TITE-Keyboard allocate a greater number of subjects to higher doses, without increasing the risk of overdosing. This feature allows for rapid identification of erroneous dose escalations (see Scenario 9). Notably, the discrepancy between historical and current DLT rates leads to an extended trial duration for the MEM-Keyboard and MEM-TITE-Keyboard designs compared to scenarios where the historical and current rates align. However, even with this prolongation, the trial duration of the MEM-Keyboard and MEM-TITE-Keyboard remains comparable to that of the Keyboard and TITE-Keyboard designs.

Across all scenarios, the performance of both the MEM-TITE-Keyboard and MEM-TITE-Keyboard (E) exhibits striking similarity, underscoring the precision of the approximation technique employed using Taylor expansion (see Supplementary Figure S1). These observations align with the conclusions illustrated by the example presented in Figure 1.

### 3.3 Sensitivity analysis

Sensitivity analysis in this Section is performed to investigate the robustness of MEM-Keyboard and MEM-TITE-Keyboard to specification of the prior $\pi \left(\Omega_{jk}\right)$ . Here, MEMs consider specific smoothing parameters that reflect the extent to which historical trials should be considered exchangeable $\pi \left(S_{h}=s_{h,k}\right)$ . To ensure an objective analysis, we limit consideration to the prior exchangeability probability that take only two values. Table 4 presents an example of how weights are assigned to MEMs. $\pi \left(S_{h}=s_{h,k}\right)$ can also be considered as a measure of tolerance for pooling in the presence of heterogeneity. In addition to the presented simulation results in Figure 2 ( $\pi \left(S_{h}=s_{h,k}\right)=$ 0.1), we compared different prior exchangeability probabilities ( $\pi \left(S_{h}=s_{h,k}\right)=$ 0.05/0.2/0.5) in our simulation with 10,000 simulated trials in each. All simulation settings are the same as those in Figure 2.

**TABLE 4:** Weights for each MEM with different prior exchangeability probabilities (historical DLT rate for current dose level is 3/8, 3/5, and 3/7).

MEM	*C*	S_1_	S_2_	S_3_	π = 0.05	π = 0.1	π = 0.2	π = 0.5
Ω_1_	1	0	0	0	0.8091	0.6472	0.3995	0.0647
Ω_2_	1	1	0	0	0.0650	0.1098	0.1525	0.0988
Ω_3_	1	0	1	0	0.0487	0.0822	0.1141	0.0740
Ω_4_	1	0	0	1	0.0619	0.1046	0.1453	0.0941
Ω_5_	1	1	1	0	0.0042	0.0149	0.0466	0.1208
Ω_6_	1	1	0	1	0.0063	0.0224	0.0699	0.1813
Ω_7_	1	0	1	1	0.0044	0.0156	0.0489	0.1267
Ω_8_	1	1	1	1	0.0004	0.0033	0.0231	0.2395

Assuming there are three independent historical trials at *j*-th dose level, there exist *K* = 2^*H*^ possible exchangeability models (Ω_*k*_). *S*_*h*_ indicates whether trial *C* and trial *H* are exchangeable (*S*_*h*_ = 1 for exchangeable and *S*_*h*_ = 0 for non-exchangeable). π denotes the different prior exchangeability probabilities, e.g., π(*S*_*h*_ = 1) = 0.1 and π(*S*_*h*_ = 0) = 0.9.

The impact of prior specification on the design is elaborately presented in Supplementary Tables S5, S6, S7. It can be observed that the proposed MEM-Keyboard and MEM-TITE-Keyboard designs are robust to the prior specification in the model when current trial is homogeneous with historical trials. In this case, borrowing information from homogeneous historical trials has little impact on the decision-making of the current trial. However, the proposed designs are sensitive to the prior specification in the model when heterogeneity exists between current trial and historical trials. This sensitivity arises due to the small sample size of only three patients at each cohort. When borrowing a substantial amount of historical information, i.e., setting a relatively large prior exchangeability probability, the historical trials may dominate the current trial and may lead to incorrect dose escalation or de-escalation decisions. Therefore, when employing the MEM-Keyboard and MEM-TITE-Keyboard designs, determination of the prior specification in the model should be fully considered. Effective supplementary sample size (ESSS) is recommended to specify the prior exchangeability probability, $\text{ESSS}\left(\Omega_{jk}\right)=\sum_{k=1}^{K}\omega_{k}\left[\alpha +\beta +\sum_{h=1}^{H}s_{h,k}n_{jh}\right]$ . When the ESSS is smaller than the number of patients enrolled at the current dose level, both MEM-Keyboard and MEM-TITE-Keyboard designs demonstrate ideal statistical performance.

### 3.4 Application

To elucidate how the proposed method leverages information in real-world scenarios, we have considered redesign of the trial pertaining to determine the MTD of sorafenib in Japanese patients with advanced refractory solid tumors by Minami et al. (2008). Sorafenib is an orally administered multi-kinase inhibitor that slows tumor growth by disrupting tumor microvasculature through antiproliferative, antiangiogenic, and/or proapoptotic effects.

A systematic review is initially conducted for previously completed trials that had been documented in the literature and historical trials that were deemed inappropriate for information borrowing were screened out. For instance, the study conducted by Clark et al. (2005) employed varying dosing regimens (1 week on, 1 week off) instead of the twice-daily (bid) administration, and was thus excluded. Ultimately, a total of three historical trials were included and patient inclusion criteria, dosages, and DLT definitions were similar and comparable to the redesigned trial. Table 5 summarizes the numbers of patients treated and numbers of observed DLTs at each dose level in these trials.

**TABLE 5:** Results of four phase I clinical trials on sorafenib monotherapy. The numbers of patients experiencing DLT events and the numbers of evaluable patients are given.

Study	Dose (mg)				
	100	200	400	600	800
Strumberg et al. (2005)	1/5	1/6	0/15	4/14	2/7
Moore et al. (2005)	0/3	1/6	0/8	3/7	
Furuse et al. (2008)		0/12	0/14		
Minami et al. (2008)	**0/3**	**1/12**	**0/6**	**1/6**	
Mean DLT rate	0.09	0.08	0.00	0.30	0.29
isotonic regression estimates of DLT rates	0.04	0.04	0.04	0.31	0.33

There are 4 candidate doses in current trial, namely 100, 200, 400, and 600 mg. Since the pooled toxicity probability violates the assumption that the dose–toxicity curves are monotone increasing, we obtained the isotonic regression estimates using the pool-adjacent-violators (PAVA) algorithm (Bril et al., 2018). And we assumed the true DLT rates of these four doses is 0.04, 0.04, 0.04 and 0.31. Using 0.31 as the target toxicity probability, 600 mg was declared as the MTD. A total of five design methods were compared here, including 3 + 3, Keyboard, MEM-Keyboard, TITE-Keyboard and MEM-TITE-Keyboard. A maximum of 24 patients would be treated in cohort sizes of 3, and trial will be stopped ahead of time when the number of patients treated at any dose reaches 6 and the next cohort is still allocated to this dose.

The statistical performance of all five designs under current setting is shown in Table 6. Compared to the Keyboard, the MEM-Keyboard design exhibits an improvement in the percentage of dose selection, increasing the likelihood of accurately identifying MTD by 5%. Furthermore, this enhancement is accompanied by a reduction in both trial duration and the average required sample size. Similar findings are observed when making a comparative analysis between the TITE-Keyboard design and the MEM-TITE-Keyboard design.

**TABLE 6:** Simulation results for each method for the redesigned dose-finding trial.

Methods		Dose (mg)				Duration	Stop (%)	Overdose (%)	Risk of poor allocation (%)	Sample size
		100	200	400	600					
3 + 3	selection (%)	1.6	1.7	49.2	**45.8**	18.2	0.5	0.0	58.9	13.9
	pts at MTD	3.3	3.3	3.2	**4.1**					
Keyboard	selection (%)	1.9	1.6	12.8	**83.6**	25.2	0.0	0.0	10.1	19.3
	pts at MTD	3.4	3.3	4.7	**7.8**					
MEM-Keyboard	selection (%)	0.1	0.4	11.4	**88.1**	24.8	0.0	0.0	0.1	19.0
	pts at MTD	3.0	3.0	4.5	**8.5**					
TITE-Keyboard	selection (%)	1.8	2.1	12.5	**83.5**	12.7	0.0	0.0	12.9	20.0
	pts at MTD	3.6	3.5	5.5	**7.3**					
MEM-TITE-Keyboard	selection (%)	0.1	0.5	13.2	**86.1**	12.3	0.0	0.0	2.7	18.8
	pts at MTD	3.0	3.0	4.2	**8.5**					

## 4 Discussion

This manuscript introduces two novel designs, MEM-Keyboard and MEM-TITE-Keyboard, aimed at expediting the drug development process and enhancing the efficiency of early-phase clinical trials. By leveraging historical trial data into current trial, these two designs enable more accurate selection of the MTD especially for handling the issue of late-onset toxicity, thereby effectively guiding dose-escalation decisions. Notably, MEM-TITE-Keyboard allows for dose escalation decisions without waiting for DLT evaluation of all previously enrolled patients, thereby significantly reducing the trial duration. Simulation studies show that both MEM-Keyboard and MEM-TITE-Keyboard designs achieve a higher percentage of correct selection, resulting in a possible reduction of required resources compared to conventional trials. Moreover, these designs offer greater flexibility by accommodating different DLT assessment windows between historical and current trials, surpassing the limitations of previous information borrowing approaches while maintaining statistical performance on par with Keyboard and TITE-Keyboard designs. The DLT assessment windows varied across different Phase I trials are addressed by utilizing approximated likelihood function, thus the proposed method remains the simplicity and flexibility of model-assisted designs.

However, these two designs are sensitive to the specification of model priors especially when heterogeneity exists between current trial and historical trials, as shown in Section 3.3. Hence, a meticulous evaluation of heterogeneity between historical and current trials becomes imperative to ensure the inclusion of primarily homogeneous historical trial data in the analysis when borrowing information. Diverse factors, including variations in dosing regimens, patient inclusion and exclusion criteria, as well as the definitions of DLT, may potentially introduce discrepancies to the trial outcomes. For instance, more frequent administration or the inclusion of patients with impaired liver or kidney function could lead to higher DLT rates. Therefore, a comprehensive consideration of these factors is crucial to appropriately exclude heterogeneous historical trial data and mitigate potential biases.

Another limitation of this design is that, the proposed design focuses solely on toxicity, overlooking the crucial aspect of efficacy. It is a common practice to extend the proposed design to Phase I-II studies by taking efficacy into consideration. However, as the field of precise medicine continues to evolve, personalized clinical trials may trigger the delayed effects of efficacy in the case of novel anti-tumor therapies. The current designs might not adequately address this issue, emphasizing the need for a novel approach that can handle both delayed efficacy and late-onset toxicity while considering impact of treatments and variability among individual subjects to ensure each participant receives an optimal treatment dose tailored to their specific characteristics, maximize therapeutic benefits while minimize the risk of adverse effects. Finally, drug-drug combination trials represent another prevalent clinical paradigm in practice of trial design. It is challenging to extend the proposed designs to situations when historical data may only be available for individual drugs rather than the combination itself. Issues mentioned above warrant in-depth exploration in future research.

## Data Availability

The original contributions presented in the study are included in the article/Supplementary Material, further inquiries can be directed to the corresponding authors.
