# Supplementary material for: Enhancing phase I dose-finding trials design through dynamic borrowing information and handling late-onset toxicity
Source: Front Pharmacol. 2023 Nov 22;14:1266322. doi: 10.3389/fphar.2023.1266322 (PMC10703472; doi:10.3389/fphar.2023.1266322)
Supplement: Supplementary file 1 [file DataSheet3.docx]

library(matrixStats)

library(MASS)

library(UBCRM)

library(stringr)

## cal the post

get.mem.post <- function(target = target,

half.width = 0.05,

num.h,

ndose,

y.h,

n.h,

y.c,

n.c,

a = 1,

b = 1,

type = 1,

exact = FALSE,

t.c = 3,

t.h,

maxt,

pend,

t.pts){

a.d <- matrix(rep(1,num.h + 1), nrow = num.h + 1, ncol = ndose, byrow = T)

b.d <- matrix(rep(1,num.h + 1), nrow = num.h + 1, ncol = ndose, byrow = T)

y.d <- rbind(y.c,y.h)

n.d <- rbind(n.c,n.h)

m.d <- c(t.c,t.h)/maxt

## weight for MEMs

calc.MEM.betabin <- function(y.d,

n.d,

a.d,

b.d,

num.h,

ndose,

exact,

m.d,

pend,

t.pts){

## number of models = 2^H, H = num.h

mod.mtx <- as.matrix(expand.grid(rep(list(c(0,1)), num.h)))

mod.mtx <- mod.mtx[order(rowSums(mod.mtx)),]

mod.mtx <- cbind(1, mod.mtx)

colnames(mod.mtx) <- c('c',paste0('h',seq(1,num.h)))

## prod.mtx = likelihood(pj|D,omega_k)

prod.mtx <- matrix(NA, nrow = length(m.d), ncol = ndose, byrow = T)

marg.mtx <- matrix(NA, nrow = 2^num.h, ncol = ndose, byrow = T)

if (exact==FALSE) {

#prod.mtx <- beta(y.d + a.d, n.d + b.d - y.d) / beta(a.d, b.d)

for (h in 1:length(m.d)) {

prod.mtx[h,] <- beta(y.d[h,] + a.d[h,], (n.d[h,] - y.d[h,])*m.d[h] + b.d[h,]) / beta(a.d[h,], b.d[h,])

}

## calc the weight at each dose

for (j in 1:ndose) {

## prior density of pj, p.vec = prob(pj|omega_k)

## 1 for exchangeable, 0 for non-exchangeable

p.vec <- apply( t(sapply(1:dim(mod.mtx)[1], function(x) prod.mtx[,j]^(1-mod.mtx[x,]))), 1, prod )

## given the omega_k, calc the integrated marginal likelihood

## exactly marg.vec = prob(D|omega_k)

marg.vec <- (beta(a.d[,j] + mod.mtx %*% c(y.d[,j]) , b.d[,j] + mod.mtx %*% c((n.d[,j] - y.d[,j])* m.d)) / beta(a.d[,j], b.d[,j]) ) * p.vec

## prior0/1 equals to prob(omega_k), e.g. 0.5

prior1 <- rep(0.5, num.h) # 0.1

prior0 <- rep(0.5, num.h) # 0.9

if(num.h == 1){

mps <- matrix( rbind(prior0,prior1)[ paste0('prior',(mod.mtx[,1:num.h + 1])), 1], ncol=1)

}

if(num.h > 1){

mps <- sapply(1:num.h , function(x) rbind(prior0,prior1)[ paste0('prior',(mod.mtx[,1:num.h + 1])[,x]), x ])

}

## calc the weight, q.vec = wk = prob(omega_k|D)

q.vec <- marg.vec * ( rowProds(mps)/sum(rowProds(mps)) ) / sum(marg.vec * ( rowProds(mps)/sum(rowProds(mps)) ) )

marg.mtx[,j] <- q.vec

}

} else {

exactlikelihood.c <- function(p){

a1 <- y.c

b1 <- n.c - pend - y.c

f <- str_c("(p^",a1,")*((1-p)^",b1,")")

form <- c(" ")

if(length(t.pts)>0){

for (m in 1:length(t.pts)) {

form <- str_c(form, "*(1-",t.pts[m]/maxt,"*p)")

}

}

form <- str_trim(form, side = "left")

formu <- str_c(f, form)

el <- eval(parse(text = formu))

}

prlc <- integrate(exactlikelihood.c, lower = 0, upper = 1)$value

prod.mtx[1,] <- prlc

## although here is an error, it has no influence to results

for (h in 1:num.h){

for (dd in 1:ndose){

exactlikelihood.p <- function(p){

an <- y.h[h,dd]

bn <- n.h[h,dd] - y.h[h,dd]

f <- str_c("(p^",an,")*((1-",m.d[h+1],"*p)^",bn,")")

el <- eval(parse(text = f))

}

prod.mtx[h+1,dd] <- integrate(exactlikelihood.p, lower = 0, upper = 1)$value

}

}

## calc the weight at each dose

for (j in 1:ndose) {

## prior density of pj, p.vec = prob(pj|omega_k)

## 1 for exchangeable, 0 for non-exchangeable

p.vec <- apply( t(sapply(1:dim(mod.mtx)[1], function(x) prod.mtx[,j]^(1-mod.mtx[x,]))), 1, prod )

## given the omega_k, calc the integrated marginal likelihood

## exactly marg.vec = prob(D|omega_k)

## marg.vec <- (beta(a.d[,j] + mod.mtx %*% c(y.d[,j] * m.d) , b.d[,j] + mod.mtx %*% c((n.d[,j] - y.d[,j])* m.d)) / beta(a.d[,j], b.d[,j]) ) * p.vec

marg.vec <- matrix(NA, nrow = 2^num.h, ncol = 1, byrow = T)

marg.vec0 <- matrix(NA, nrow = 2^num.h, ncol = 1, byrow = T)

for (model in 1:2^num.h){

exactlikelihood.s <- function(p){

am <- y.c

bm <- n.c - pend - y.c

f <- str_c("(p^",am,")*((1-p)^",bm,")")

form <- c(" ")

if(length(t.pts)>0){

for (m in 1:length(t.pts)) {

form <- str_c(form, "*(1-",t.pts[m]/maxt,"*p)")

}

}

form <- str_trim(form, side = "left")

cform <- str_c(f, form)

hform <- c(" ")

for (hh in 1:num.h){

ah <- y.h[hh,j]

bh <- n.h[hh,j] - y.h[hh,j]

s <- mod.mtx[model,hh+1]

hform <- str_c(hform,"*(((p^",ah,")*((1-",m.d[hh+1],"*p)^",bh,"))^",s,")")

}

hform <- str_trim(hform, side = "left")

formu <- str_c(cform, hform)

el <- eval(parse(text = formu))

}

marg.vec0[model,] <- integrate(exactlikelihood.s, lower = 0, upper = 1)$value

}

marg.vec <- marg.vec0 * p.vec

## prior0/1 equals to prob(omega_k), e.g. 0.5

prior1 <- rep(0.5, num.h) # 0.1

prior0 <- rep(0.5, num.h) # 0.9

if(num.h == 1){

mps <- matrix( rbind(prior0,prior1)[ paste0('prior',(mod.mtx[,1:num.h + 1])), 1], ncol=1)

}

if(num.h > 1){

mps <- sapply(1:num.h , function(x) rbind(prior0,prior1)[ paste0('prior',(mod.mtx[,1:num.h + 1])[,x]), x ])

}

## calc the weight, q.vec = wk = prob(omega_k|D)

q.vec <- marg.vec * ( rowProds(mps)/sum(rowProds(mps)) ) / sum(marg.vec * ( rowProds(mps)/sum(rowProds(mps)) ) )

marg.mtx[,j] <- q.vec

}

}

ret <- list(q = marg.mtx, mod.mtx = mod.mtx )

return(ret)

}

## get the keys for Keyboard design

getkeys <- function(target, half.width) {

c1 = target - half.width

c2 = target + half.width

delta = c2 - c1

lkey = NULL

rkey = NULL

i = 1

cutoff = c1 - (i * delta)

while (cutoff > 0) {

lkey = c(cutoff, lkey)

i = i + 1

cutoff = c1 - (i * delta)

}

lkey[lkey < 0] = 0

i = 1

cutoff = c2 + (i * delta)

while(cutoff < 1) {

rkey = c(rkey, cutoff)

i = i + 1

cutoff = c2 + (i * delta)

}

rkey[rkey > 1] = 1

keys = c(lkey, c1, c2, rkey)

return(keys)

}

## calc the posterior

if (type == 1){

posterior.prob <- function(y.d,

n.d,

a.d,

b.d,

a = 1,

b = 1,

mod.mtx=NULL,

mod.weight=NULL){

if (exact==FALSE) {

mod.mtx <- calc.MEM.betabin(y.d = y.d,

n.d = n.d,

a.d = b.d,

b.d = b.d,

num.h = num.h,

ndose = ndose,

exact = FALSE,

m.d = m.d,

pend = pend,

t.pts = t.pts)$mod.mtx

mod.weight <- calc.MEM.betabin(y.d = y.d,

n.d = n.d,

a.d = b.d,

b.d = b.d,

num.h = num.h,

ndose = ndose,

exact = FALSE,

m.d = m.d,

pend = pend,

t.pts = t.pts)$q

z.adj <- y.adj <- matrix(NA,nrow = length(m.d), ncol = ndose, byrow = T)

for (hh in 1:length(m.d)) {

z.adj[hh,] <- (n.d[hh,] - y.d[hh,]) * m.d[hh]

y.adj[hh,] <- y.d[hh,]

}

z.borrow <- mod.mtx %*% z.adj

y.borrow <- mod.mtx %*% y.adj

alpha <- a + y.borrow

beta <- b + z.borrow

keys <- getkeys(target = target, half.width = half.width)

mem.post <- matrix(NA,nrow = ndose, ncol = length(keys)-1, byrow = T)

rownames(mem.post) <- c(paste0('dose',seq(1,ndose)))

colnames(mem.post) <- c(paste0('key',seq(1,length(keys)-1)))

## sum(wjk * prob(pjk belongs to Im|omega_jk,Dj))

## for each dose, sum(wk * prob(pk belongs to Im|omega_k,D))

for (j in 1:ndose){

key.post <- matrix(NA,nrow = 2^num.h, ncol = length(keys)-1, byrow = T)

rownames(key.post) <- c(paste0('model',seq(1,2^num.h)))

colnames(key.post) <- c(paste0('key',seq(1,length(keys)-1)))

a.j <- alpha[,j]

b.j <- beta[,j]

for(u in 1:dim(mod.mtx)[1]){

for (m in 1:(length(keys)-1)){

key.post[u,m] <- as.numeric(pbeta( keys[m+1],a.j[u],b.j[u] ) - pbeta( keys[m],a.j[u],b.j[u]) )

}

}

for (p in 1:(length(keys)-1)){

mem.post[j,p] <- key.post[,p] %*% mod.weight[,j]

}

}

} else {

mod.mtx <- calc.MEM.betabin(y.d = y.d,

n.d = n.d,

a.d = b.d,

b.d = b.d,

num.h = num.h,

ndose = ndose,

exact = TRUE,

m.d = m.d,

pend = pend,

t.pts = t.pts)$mod.mtx

mod.weight <- calc.MEM.betabin(y.d = y.d,

n.d = n.d,

a.d = b.d,

b.d = b.d,

num.h = num.h,

ndose = ndose,

exact = TRUE,

m.d = m.d,

pend = pend,

t.pts = t.pts)$q

keys <- getkeys(target = target, half.width = half.width)

mem.post <- matrix(NA,nrow = ndose, ncol = length(keys)-1, byrow = T)

rownames(mem.post) <- c(paste0('dose',seq(1,ndose)))

colnames(mem.post) <- c(paste0('key',seq(1,length(keys)-1)))

for (j in 1:ndose){

## although here is an error, it has no influence to results

key.post <- matrix(NA,nrow = 2^num.h, ncol = length(keys)-1, byrow = T)

rownames(key.post) <- c(paste0('model',seq(1,2^num.h)))

colnames(key.post) <- c(paste0('key',seq(1,length(keys)-1)))

exl <- matrix(NA, nrow = 2^num.h, ncol = 1, byrow = T)

for (model in 1:2^num.h){

exactlikelihood.s <- function(p){

am <- y.c

bm <- n.c - pend - y.c

f <- str_c("(p^",am,")*((1-p)^",bm,")")

form <- c(" ")

if(length(t.pts)>0){

for (m in 1:length(t.pts)) {

form <- str_c(form, "*(1-",t.pts[m]/maxt,"*p)")

}

}

form <- str_trim(form, side = "left")

cform <- str_c(f, form)

hform <- c(" ")

for (hh in 1:num.h){

ah <- y.h[hh,j]

bh <- n.h[hh,j] - y.h[hh,j]

s <- mod.mtx[model,hh+1]

hform <- str_c(hform,"*(((p^",ah,")*((1-",m.d[hh+1],"*p)^",bh,"))^",s,")")

}

hform <- str_trim(hform, side = "left")

formu <- str_c(cform, hform)

el <- eval(parse(text = formu))

}

exl[model,] <- integrate(exactlikelihood.s, lower = 0, upper = 1)$value

}

for(u in 1:dim(mod.mtx)[1]){

for (m in 1:(length(keys)-1)){

exactpost <- function(p){

am <- y.c

bm <- n.c - pend - y.c

f <- str_c("(p^",am,")*((1-p)^",bm,")")

form <- c(" ")

if(length(t.pts)>0){

for (m in 1:length(t.pts)) {

form <- str_c(form, "*(1-",t.pts[m]/maxt,"*p)")

}

}

form <- str_trim(form, side = "left")

cform <- str_c(f, form)

hform <- c(" ")

for (hh in 1:num.h){

ah <- y.h[hh,j]

bh <- n.h[hh,j] - y.h[hh,j]

s <- mod.mtx[u,hh+1]

hform <- str_c(hform,"*(((p^",ah,")*((1-",m.d[hh+1],"*p)^",bh,"))^",s,")")

}

hform <- str_trim(hform, side = "left")

formu <- str_c(cform, hform)

el <- eval(parse(text = formu))

}

key.post[u,m] <- (integrate(exactpost, lower = keys[m], upper = keys[m+1])$value)/(exl[u,1])

}

}

for (p in 1:(length(keys)-1)){

mem.post[j,p] <- key.post[,p] %*% mod.weight[,j]

}

}

}

return(mem.post)

}

}

if (type == 2){

posterior.prob <- function(y.d,

n.d,

a.d,

b.d,

a = 1,

b = 1,

mod.mtx=NULL,

mod.weight=NULL){

mod.mtx <- calc.MEM.betabin(y.d = y.d,

n.d = n.d,

a.d = b.d,

b.d = b.d,

num.h = num.h)$mod.mtx

mod.weight <- calc.MEM.betabin(y.d = y.d,

n.d = n.d,

a.d = b.d,

b.d = b.d,

num.h = num.h)$q

n.borrow <- mod.mtx %*% n.d

y.borrow <- mod.mtx %*% y.d

alpha <- a + y.borrow

beta <- b + n.borrow - y.borrow

keys <- getkeys(target = target, half.width = half.width)

mem.post <- matrix(NA,nrow = ndose, ncol = length(keys)-1, byrow = T)

rownames(mem.post) <- c(paste0('dose',seq(1,ndose)))

colnames(mem.post) <- c(paste0('key',seq(1,length(keys)-1)))

## sum(wjk * prob(pjk belongs to Im|omega_jk,Dj))

## for each dose, sum(wk * prob(pk belongs toIm|omega_k,D))

for (j in 1:ndose){

key.post <- matrix(NA,nrow = 2^num.h, ncol = length(keys)-1, byrow = T)

rownames(key.post) <- c(paste0('model',seq(1,2^num.h)))

colnames(key.post) <- c(paste0('key',seq(1,length(keys)-1)))

a.j <- alpha[,j]

b.j <- beta[,j]

## calc the empirical mixed distribution

x1 <- new_BETA(a.j[1],b.j[1])

x2 <- new_BETA(a.j[2],b.j[2])

x3 <- new_BETA(a.j[3],b.j[3])

x4 <- new_BETA(a.j[4],b.j[4])

x5 <- new_BETA(a.j[5],b.j[5])

x6 <- new_BETA(a.j[6],b.j[6])

x7 <- new_BETA(a.j[7],b.j[7])

x8 <- new_BETA(a.j[8],b.j[8])

xnew <- new_MIXTURE(list(x1,x2,x3,x4,x5,x6,x7,x8),c(mod.weight[,j]))

## empirical probability density function

xx <- rfunc(xnew, 10000)

xxdf <- tidyr::gather(data.frame(xx), dimension, value)

epdf.den <- density(xxdf$value)

epdf <- matrix(NA,nrow = length(epdf.den$y), ncol = 2, byrow = T)

epdf[,1] <- epdf.den$x

epdf[,2] <- epdf.den$y

## find the strongest key

# order.max <- which(m$y==max(m$y),arr.ind=TRUE)

# xmax <- m$x[order.max]

## empirical cumulative distribution function

x <- sort(xxdf$value)

n <- length(x)

xaxis <- unique(x)

yaxis <- cumsum(tabulate(match(x, xaxis)))/n

ecdf <- matrix(NA,nrow = length(xaxis), ncol = 2, byrow = T)

ecdf[,1] <- xaxis

ecdf[,2] <- yaxis

## calc the density

keys.cdf <- matrix(NA,nrow = length(keys), ncol = 1, byrow = T)

for (p in 1:length(keys)){

xp <- abs(ecdf[,1]-keys[p])

order.min <- which(xp ==min(xp),arr.ind=TRUE)

keys.cdf[p,1] <- ecdf[,2][order.min]

}

for (m in 1:(length(keys)-1)){

mem.post[j,m] <- as.numeric(keys.cdf[m+1,1] - keys.cdf[m,1])

}

}

return(mem.post)

}

}

return(posterior.prob(y.d,

n.d,

a.d,

b.d,

a = 1,

b = 1,

mod.mtx=NULL,

mod.weight=NULL))

}

## cal the oc of titememkb

get.oc.titemem <- function(target, p.true, ncohort, cohortsize, maxt=1, prior.p=NA, accrual=3, maxpen=0.5,

dist1=1, dist2=1,alpha=0.5,n.earlystop = 100, startdose = 1,

p.saf = target-0.05, p.tox = target+0.05, cutoff.eli = 0.95,

extrasafe = FALSE, offset = 0.05, ntrial = 1000, seed=NULL, design=1,

num.h, ndose, y.h, n.h, type = 1, exact = FALSE, t.c = 3, t.h)

{

select.mtd <- function(target, npts, ntox, cutoff.eli = 0.95, extrasafe = FALSE, offset = 0.05, verbose = TRUE) {

## isotonic transformation using the pool adjacent violator algorithm (PAVA)

pava <- function(x, wt = rep(1, length(x))) {

n <- length(x)

if (n <= 1)

return(x)

if (any(is.na(x)) || any(is.na(wt))) {

stop("Missing values in 'x' or 'wt' not allowed")

}

lvlsets <- (1:n)

repeat {

viol <- (as.vector(diff(x)) < 0)

if (!(any(viol)))

break

i <- min((1:(n - 1))[viol])

lvl1 <- lvlsets[i]

lvl2 <- lvlsets[i + 1]

ilvl <- (lvlsets == lvl1 | lvlsets == lvl2)

x[ilvl] <- sum(x[ilvl] * wt[ilvl])/sum(wt[ilvl])

lvlsets[ilvl] <- lvl1

}

x

}

## determine whether the dose has been eliminated during the trial

y = ntox

n = npts

ndose = length(n)

elimi = rep(0, ndose)

for (i in 1:ndose) {

if (n[i] >= 3) {

if (1 - pbeta(target, y[i] + 1, n[i] - y[i] + 1) > cutoff.eli) {

elimi[i:ndose] = 1

break

}

}

}

if (extrasafe) {

if (n[1] >= 3) {

if (1 - pbeta(target, y[1] + 1, n[1] - y[1] + 1) > cutoff.eli - offset) {

elimi[1:ndose] = 1

}

}

}

## no dose should be selected (i.e., selectdose=99) if the first dose is already very toxic or all uneliminated doses are never used to treat patients

if (elimi[1] == 1 || sum(n[elimi == 0]) == 0) {

selectdose = 99

} else {

adm.set = (n != 0) & (elimi == 0)

adm.index = which(adm.set == T)

y.adm = y[adm.set]

n.adm = n[adm.set]

## poster mean and variance of toxicity probabilities using beta(0.05, 0.05) as the prior

phat = (y.adm + 0.05)/(n.adm + 0.1)

phat.var = (y.adm + 0.05) * (n.adm - y.adm + 0.05)/((n.adm + 0.1)^2 * (n.adm + 0.1 + 1))

## perform the isotonic transformation using PAVA

phat = pava(phat, wt = 1/phat.var)

phat = phat + (1:length(phat)) * 1e-10 ## break ties by adding an increasingly small number

selectd = sort(abs(phat - target), index.return = T)$ix[1] ## select dose closest to the target as the MTD

selectdose = adm.index[selectd]

}

if (verbose == TRUE) {

if (selectdose == 99) {

out = list(target = target, MTD = selectdose,

p_est = data.frame(cbind('dose'=1:length(npts), 'phat'=rep("----",length(npts)),

'CI'=paste("(", rep("----",length(npts)),",",rep("----",length(npts)),")",sep="")))

)

} else {

trtd = (n != 0)

poverdose = pava(1 - pbeta(target, y[trtd] + 0.05, n[trtd] - y[trtd] + 0.05))

phat.all = pava((y[trtd] + 0.05)/(n[trtd] + 0.1), wt = 1/((y[trtd] + 0.05) * (n[trtd] - y[trtd] + 0.05)/((n[trtd] + 0.1)^2 * (n[trtd] + 0.1 + 1))))

A1 = A2 = NA

A3 = NA

A4 = A5 = NA

## output summary statistics

for (i in 1:ndose) {

if (n[i] > 0) {

A1 = append(A1, formatC(phat.all[i], digits = 2, format = "f"))

A2 = append(A2, formatC(qbeta(0.025, y[i] + 0.05, n[i] - y[i] + 0.05), digits = 2, format = "f"))

A3 = append(A3, formatC(qbeta(0.975, y[i] + 0.05, n[i] - y[i] + 0.05), digits = 2, format = "f"))

A4 = append(A4, formatC(poverdose[i], digits = 2, format = "f"))

} else {

# no estimate output for doses never used to treat patients

A1 = append(A1, "----")

A2 = append(A2, "----")

A3 = append(A3, "----")

A4 = append(A4, "----")

}

}

p_est = data.frame(cbind('dose'=1:length(npts), 'phat'=A1[-1], 'CI'=paste("(", A2[-1],",",A3[-1],")",sep="")))

out = list(target = target, MTD = selectdose, p_est=p_est, p_overdose = A4[-1])

}

} else {

out = list(target = target, MTD = selectdose)

}

return(out)

}

gen.tite<-function(dist=1, n, pi, alpha=0.5, Tobs=1)

{

############ subroutines ############

weib<-function(n, pi, pihalft)

{

## solve parameters for Weibull given pi=1-S(T) and phalft=1-S(T/2)

alpha = log(log(1-pi)/log(1-pihalft))/log(2);

lambda = -log(1-pi)/(Tobs^alpha);

t = (-log(runif(n))/lambda)^(1/alpha);

return(t);

}

llogit<-function(n, pi, pihalft)

{

## solve parameters for log-logistic given pi=1-S(T) and phalft=1-S(T/2)

alpha = log((1/(1-pi)-1)/(1/(1-pihalft)-1))/log(2);

lambda = (1/(1-pi)-1)/(Tobs^alpha);

t = ((1/runif(n)-1)/lambda)^(1/alpha);

return(t);

}

############ end of subroutines ############

tox = rep(0, n);

t.tox = rep(0, n);

#### uniform

if(dist==1) { # 50% event in (0, 1/2T)

tox = rbinom(n, 1, pi);

ntox.st = sum(tox);

t.tox[tox==0]=Tobs;

t.tox[tox==1]=runif(ntox.st, 0, Tobs);

}

#### Weibull

if(dist==2)

{

pihalft = alpha*pi; # alpha*100% event in (0, 1/2T)

t.tox = weib(n, pi, pihalft);

tox[t.tox<=Tobs]=1;

ntox.st = sum(tox);

t.tox[tox==0]=Tobs;

}

#### log-logistic

if(dist==3)

{

pihalft = alpha*pi; # alpha*100% event in (0, 1/2T)

t.tox = llogit(n, pi, pihalft);

tox[t.tox<=Tobs]=1;

ntox.st = sum(tox);

t.tox[tox==0]=Tobs;

}

return(list(tox=tox, t.tox=t.tox, ntox.st=ntox.st));

}

### simple error checking

if(target<0.05) {cat("Error: the target is too low! \n"); return();}

if(target>0.6) {cat("Error: the target is too high! \n"); return();}

if((target-p.saf)<(0.1*target)) {cat("Error: the probability deemed safe cannot be higher than or too close to the target! \n"); return();}

if((p.tox-target)<(0.1*target)) {cat("Error: the probability deemed toxic cannot be lower than or too close to the target! \n"); return();}

if(p.saf<0.05) {cat("Error: the lower interval boundary cannot be too close to 0! \n"); return();}

if(p.tox>0.95) {cat("Error: the upper interval boundary cannot be too close to 1! \n"); return();}

if(offset>=0.5) {cat("Error: the offset is too large! \n"); return();}

if(!is.na(prior.p[1])){if(length(prior.p)!=3){cat("Error: The length of the prior probabilities should be 3! \n"); return();}}

if(n.earlystop<=6) {cat("Warning: the value of n.earlystop is too low to ensure good operating characteristics. Recommend n.earlystop = 9 to 18 \n"); return();}

if(is.na(maxpen)){maxpen=0.5;}

if(maxpen<0 || maxpen>0.65) {cat("Error: the value of maxpen should lie within (0,0.65]! \n"); return();}

epi1<-target-p.saf;

epi2<-p.tox-target;

set.seed(seed);

if(is.na(prior.p[1])){prior.p = rep(1/3,3)}

prior.p = prior.p/sum(prior.p)

ndose = length(p.true);

Y = matrix(rep(0, ndose * ntrial), ncol = ndose);

N = matrix(rep(0, ndose * ntrial), ncol = ndose);

dselect = rep(0, ntrial);

durationV = rep(0, ntrial);

npendV = rep(0, ntrial);

npts = ncohort*cohortsize;

a<-b<-1

for(trial in 1:ntrial)

{

y=NULL; #toxicity indicator for each subject

dv=NULL; #dose for each subject

n.d = rep(0, ndose); # number of toxicity at each dose

y.d = rep(0, ndose); # number of patient at each dose

t.enter=NULL; # time enter the study

t.event=NULL; # time to event

t.decision = 0; # decision making time

d = startdose; # current dose level

earlystop = 0; #indicate if trial stops early

nearlystop = 0; #indicate if npts at dose level reach the limit

elimi = rep(0, ndose)

npend = 0;

for(i in 1:ncohort)

{

# generate data for the new patient

for(j in 1:cohortsize)

{

if(j==1) { t.enter = c(t.enter, t.decision); }

else {

if(dist2==1){ t.enter = c(t.enter, t.enter[length(t.enter)] + runif(1, 0, 2/accrual))}

if(dist2==2){ t.enter = c(t.enter, t.enter[length(t.enter)] + rexp(1, rate=accrual))}

}

}

obscohort = gen.tite(dist1, cohortsize, p.true[d], alpha=alpha,T=maxt);

t.event = c(t.event, obscohort$t.tox);

y = c(y, obscohort$tox);

dv = c(dv, rep(d, cohortsize));

t.decision = t.enter[length(t.enter)];

nobs=-1; pending=1;

d.curr=d;

npend = npend-1;

while(pending==1)

{

npend = npend+1;

pending = 0;

if(i==ncohort) { t.decision = t.decision + maxt; } else {

if(dist2==1){t.decision = t.decision + runif(1, 0, 2/accrual)}

if(dist2==2){t.decision = t.decision + rexp(1, rate=accrual)}

}

# determine which observation are observed

delta = ((t.enter+t.event)<=t.decision);

t = pmin(t.event, t.decision-t.enter, maxt); ## used for recording potential censoring time

cset = (dv==d);

delta.curr = delta[cset];

t.curr = t[cset];

ntox.curr = sum((y[cset])[delta.curr==1]);

#totalt = sum(t.curr[delta.curr==0])/maxt;

totalt = t.curr[delta.curr==0]

t.npts = t.curr[delta.curr==0]

totalt = 3*prior.p[1]*totalt*(totalt<=maxt/3)+

((prior.p[1]-prior.p[2])*maxt+3*prior.p[2]*totalt)*(maxt/3<totalt & totalt<=2*maxt/3)+

((prior.p[1]+prior.p[2]-2*prior.p[3])*maxt+3*prior.p[3]*totalt)*(2*maxt/3<totalt & totalt<=maxt)

totalt = sum(totalt)/maxt

n.curr = sum(cset);

n.pend = sum(delta[cset]==0);

nobs = sum(delta[cset]);

# determine which dose level should be eliminated

for(dd in 1:ndose){

cset1 = dv==dd;

delta.curr1 = delta[cset1];

ntox.curr1 = sum((y[cset1])[delta.curr1==1]);

n.curr1 = sum(cset1);

if (1-pbeta(target, ntox.curr1+1, n.curr1-ntox.curr1+1)>cutoff.eli && n.curr1>=3){

elimi[dd:ndose]=1;

break;

}

}

#check whether extra safety rule should be applied

if(extrasafe)

{

if(d==1){

if(1-pbeta(target, ntox.curr+1, n.curr-ntox.curr+1)>cutoff.eli-offset && n.curr>=3) {

earlystop = 1; break;}

}

}

# check whether the current dose level should be eliminated

if(elimi[d.curr]==1) {

d=which(elimi==1)[1]-1

if(d==0){earlystop = 1; break;}

next;

}

#check whether the trial should be early terminated

if(n.curr>=n.earlystop){nearlystop = 1;break;}

#check whether the current dose is toxic based on observed data

q0<-pbeta(target+epi2,ntox.curr+a,n.curr-ntox.curr+b)-pbeta(target-epi1,ntox.curr+a,n.curr-ntox.curr+b)

q1<-pbeta(target-epi1,ntox.curr+a,n.curr-ntox.curr+b)-pbeta(target-2*epi1-epi2,ntox.curr+a,n.curr-ntox.curr+b)

q2<-pbeta(target+epi1+2*epi2,ntox.curr+a,n.curr-ntox.curr+b)-pbeta(target+epi2,ntox.curr+a,n.curr-ntox.curr+b)

if(q2>q1 & q2>q0){

if(d==1){d=d; if(n.pend>0){pending=1};} else{d=d-1};

next;

}

#check whether the trial should be suspended

if(n.pend<0) {pending=1;}

else

{

y0<-ntox.curr

n0<-n.curr-n.pend+totalt

if(design==2){

q0<-pbeta(target+epi2,y0+1,n0-y0+1)-pbeta(target-epi1,y0+1,n0-y0+1)

q0<-q0/(epi2+epi1)

q1<-pbeta(target-epi1,y0+1,n0-y0+1)-pbeta(0,y0+1,n0-y0+1)

q1<-q1/(target-epi1)

q2<-pbeta(1,y0+1,n0-y0+1)-pbeta(target+epi2,y0+1,n0-y0+1)

q2<-q2/(1-target-epi2)

} else {

q0<-pbeta(target+epi2,y0+a,n0-y0+b)-pbeta(target-epi1,y0+a,n0-y0+b)

q1<-pbeta(target-epi1,y0+a,n0-y0+b)-pbeta(target-2*epi1-epi2,y0+a,n0-y0+b)

q2<-pbeta(target+epi1+2*epi2,y0+a,n0-y0+b)-pbeta(target+epi2,y0+a,n0-y0+b)

}

if (exact==FALSE){

y0<-ntox.curr

n0<-n.curr-n.pend+totalt

pj.post <- get.mem.post(target = target,

half.width = 0.05,

num.h,

ndose,

y.h,

n.h,

y.c = y0,

n.c = n0,

a = 1,

b = 1,

type = 1,

exact = FALSE,

t.c = 3,

t.h,

maxt,

pend = n.pend,

t.pts = t.npts)

} else {

pj.post <- get.mem.post(target = target,

half.width = 0.05,

num.h,

ndose,

y.h,

n.h,

y.c = ntox.curr,

n.c = n.curr,

a = 1,

b = 1,

type = 1,

exact = TRUE,

t.c = 3,

t.h,

maxt,

pend = n.pend,

t.pts = t.npts)

}

mq0<-pj.post[d,3]

mq1<-pj.post[d,2]

mq2<-pj.post[d,4]

if(mq1>mq0 & mq1>mq2){

if(nobs<=2){

if(n.curr==1){d=d;} else {if(n.pend==0){d=min(d+1,ndose)} else {pending=1;}}

} else {

#check whether the current dose is the highest

if(d==ndose){d=d;} else{

if(elimi[d+1]==1){d=d} else{ d=d+1}

}}} else if (mq2>mq0 & mq2>mq1){

if(d==1){d=d} else {d=d-1}

} else {d=d;

}

if(elimi[d]==1){d=which(elimi==1)[1]-1}

}

}

if(earlystop==1){break;}

if(nearlystop==1){break;}

}

for(k in 1:ndose){

y.d[k] = sum(y[dv==k]);

n.d[k] = sum(dv==k);

}

npendV[trial]= npend;

Y[trial, ] = y.d

N[trial, ] = n.d

durationV[trial] = t.decision

if (earlystop == 1) {

dselect[trial] = 99

}

else {dselect[trial] = select.mtd(target, n.d, y.d, cutoff.eli, extrasafe, offset, verbose=FALSE)$MTD}

}

selpercent = rep(0, ndose)

selpercent=rep(0, ndose);

nptsdose = apply(N,2,mean);

ntoxdose = apply(Y,2,mean);

for(i in 1:ndose) { selpercent[i]=sum(dselect==i)/ntrial*100; }

if(length(which(p.true==target))>0) # if MTD exists, calculate risk of overdosing

{

if (which(p.true==target) == ndose-1) {

overdosing60=mean(N[,p.true>target]>0.6*npts)*100;

overdosing80=mean(N[,p.true>target]>0.8*npts)*100;

} else {

overdosing60=mean(rowSums(N[,p.true>target])>0.6*npts)*100;

overdosing80=mean(rowSums(N[,p.true>target])>0.8*npts)*100;

}

out=list(selpercent=selpercent, npatients=nptsdose, ntox=ntoxdose, totaltox=sum(Y)/ntrial, totaln=sum(N)/ntrial,

percentstop=sum(dselect== 99)/ntrial*100, poorallocation=mean(N[, p.true==target]<npts/ndose)*100,

overdose60=overdosing60, overdose80=overdosing80, duration=mean(durationV),sdduration=sqrt(var(durationV)),simu.setup=data.frame(target=target, p.true=p.true, ncohort=ncohort, cohortsize = cohortsize,

startdose = startdose,p.saf = p.saf, p.tox = p.tox, cutoff.eli = cutoff.eli, extrasafe = extrasafe, offset = offset,

ntrial = ntrial, dose=1:ndose),flowchart=TRUE);

}

else {

out=list(selpercent=selpercent, npatients=nptsdose, ntox=ntoxdose, totaltox=sum(Y)/ntrial, totaln=sum(N)/ntrial,

percentstop=sum(dselect== 99)/ntrial*100, duration=mean(durationV),sdduration=sqrt(var(durationV)),simu.setup=data.frame(target=target, p.true=p.true, ncohort=ncohort, cohortsize = cohortsize,

startdose = startdose,p.saf = p.saf, p.tox = p.tox, cutoff.eli = cutoff.eli, extrasafe = extrasafe, offset = offset, ntrial = ntrial,

dose=1:ndose),flowchart=TRUE);

}

return(out);

}
