# Supplementary material for: Enhancing phase I dose-finding trials design through dynamic borrowing information and handling late-onset toxicity
Source: Front Pharmacol. 2023 Nov 22;14:1266322. doi: 10.3389/fphar.2023.1266322 (PMC10703472; doi:10.3389/fphar.2023.1266322)
Supplement: Supplementary file 2 [file DataSheet2.docx]

get.mem.post1 <- function(target = target,

half.width = 0.05,

num.h,

ndose,

y.h,

n.h,

y.c,

n.c,

a = 1,

b = 1,

type){

a.d <- matrix(rep(1,num.h + 1), nrow = num.h + 1, ncol = ndose, byrow = T)

b.d <- matrix(rep(1,num.h + 1), nrow = num.h + 1, ncol = ndose, byrow = T)

y.d <- rbind(y.c,y.h)

n.d <- rbind(n.c,n.h)

## weight for MEMs

calc.MEM.betabin <- function(y.d,

n.d,

a.d,

b.d,

num.h){

## number of models = 2^H, H = num.h

mod.mtx <- as.matrix(expand.grid(rep(list(c(0,1)), num.h)))

mod.mtx <- mod.mtx[order(rowSums(mod.mtx)),]

mod.mtx <- cbind(1, mod.mtx)

colnames(mod.mtx) <- c('c',paste0('h',seq(1,num.h)))

## prod.mtx = likelihood(pj|D,omega_k)

prod.mtx <- beta(y.d + a.d, n.d + b.d - y.d) / beta(a.d, b.d)

marg.mtx <- matrix(NA,nrow = 2^num.h, ncol = ndose, byrow = T)

## calc the weight at each dose

for (j in 1:ndose) {

## prior density of pj, p.vec = prob(pj|omega_k)

## 1 for exchangeable, 0 for non-exchangeable

p.vec <- apply( t(sapply(1:dim(mod.mtx)[1], function(x) prod.mtx[,j]^(1-mod.mtx[x,]))), 1, prod )

## given the omega_k, calc the integrated marginal likelihood

## exactly marg.vec = prob(D|omega_k)

marg.vec <- (beta(a.d[,j] + mod.mtx %*% y.d[,j] , b.d[,j] + mod.mtx %*% (n.d[,j] - y.d[,j])) / beta(a.d[,j],b.d[,j]) ) * p.vec

## prior0/1 equals to prob(omega_k), e.g. 0.5

prior1 <- rep(0.5, num.h) # 0.1

prior0 <- rep(0.5, num.h) # 0.9

if(num.h == 1){

mps <- matrix( rbind(prior0,prior1)[ paste0('prior',(mod.mtx[,1:num.h + 1])), 1], ncol=1)

}

if(num.h > 1){

mps <- sapply(1:num.h , function(x) rbind(prior0,prior1)[ paste0('prior',(mod.mtx[,1:num.h + 1])[,x]), x ])

}

## calc the weight, q.vec = wk = prob(omega_k|D)

q.vec <- marg.vec * ( rowProds(mps)/sum(rowProds(mps)) ) / sum(marg.vec * ( rowProds(mps)/sum(rowProds(mps)) ) )

marg.mtx[,j] <- q.vec

}

ret <- list(q = marg.mtx, mod.mtx = mod.mtx )

return(ret)

}

## get the keys for Keyboard design

getkeys <- function(target, half.width) {

c1 = target - half.width

c2 = target + half.width

delta = c2 - c1

lkey = NULL

rkey = NULL

i = 1

cutoff = c1 - (i * delta)

while (cutoff > 0) {

lkey = c(cutoff, lkey)

i = i + 1

cutoff = c1 - (i * delta)

}

lkey[lkey < 0] = 0

i = 1

cutoff = c2 + (i * delta)

while(cutoff < 1) {

rkey = c(rkey, cutoff)

i = i + 1

cutoff = c2 + (i * delta)

}

rkey[rkey > 1] = 1

keys = c(lkey, c1, c2, rkey)

return(keys)

}

## calc the posterior

if (type == 1){

posterior.prob <- function(y.d,

n.d,

a.d,

b.d,

a = 1,

b = 1,

mod.mtx=NULL,

mod.weight=NULL){

mod.mtx <- calc.MEM.betabin(y.d = y.d,

n.d = n.d,

a.d = b.d,

b.d = b.d,

num.h = num.h)$mod.mtx

mod.weight <- calc.MEM.betabin(y.d = y.d,

n.d = n.d,

a.d = b.d,

b.d = b.d,

num.h = num.h)$q

n.borrow <- mod.mtx %*% n.d

y.borrow <- mod.mtx %*% y.d

alpha <- a + y.borrow

beta <- b + n.borrow - y.borrow

keys <- getkeys(target = target, half.width = half.width)

mem.post <- matrix(NA,nrow = ndose, ncol = length(keys)-1, byrow = T)

rownames(mem.post) <- c(paste0('dose',seq(1,ndose)))

colnames(mem.post) <- c(paste0('key',seq(1,length(keys)-1)))

## sum(wjk * prob(pjk belongs to Im|omega_jk,Dj))

## for each dose, sum(wk * prob(pk belongs to Im|omega_k,D))

for (j in 1:ndose){

key.post <- matrix(NA,nrow = 2^num.h, ncol = length(keys)-1, byrow = T)

rownames(key.post) <- c(paste0('model',seq(1,2^num.h)))

colnames(key.post) <- c(paste0('key',seq(1,length(keys)-1)))

a.j <- alpha[,j]

b.j <- beta[,j]

for(u in 1:dim(mod.mtx)[1]){

for (m in 1:(length(keys)-1)){

key.post[u,m] <- as.numeric(pbeta( keys[m+1],a.j[u],b.j[u] ) - pbeta( keys[m],a.j[u],b.j[u]) )

}

}

for (p in 1:(length(keys)-1)){

mem.post[j,p] <- key.post[,p] %*% mod.weight[,j]

}

}

return(mem.post)

}

}

if (type == 2){

posterior.prob <- function(y.d,

n.d,

a.d,

b.d,

a = 1,

b = 1,

mod.mtx=NULL,

mod.weight=NULL){

mod.mtx <- calc.MEM.betabin(y.d = y.d,

n.d = n.d,

a.d = b.d,

b.d = b.d,

num.h = num.h)$mod.mtx

mod.weight <- calc.MEM.betabin(y.d = y.d,

n.d = n.d,

a.d = b.d,

b.d = b.d,

num.h = num.h)$q

n.borrow <- mod.mtx %*% n.d

y.borrow <- mod.mtx %*% y.d

alpha <- a + y.borrow

beta <- b + n.borrow - y.borrow

keys <- getkeys(target = target, half.width = half.width)

mem.post <- matrix(NA,nrow = ndose, ncol = length(keys)-1, byrow = T)

rownames(mem.post) <- c(paste0('dose',seq(1,ndose)))

colnames(mem.post) <- c(paste0('key',seq(1,length(keys)-1)))

## sum(wjk * prob(pjk belongs to Im|omega_jk,Dj))

## for each dose, sum(wk * prob(pk belongs toIm|omega_k,D))

for (j in 1:ndose){

key.post <- matrix(NA,nrow = 2^num.h, ncol = length(keys)-1, byrow = T)

rownames(key.post) <- c(paste0('model',seq(1,2^num.h)))

colnames(key.post) <- c(paste0('key',seq(1,length(keys)-1)))

a.j <- alpha[,j]

b.j <- beta[,j]

## calc the empirical mixed distribution

x1 <- new_BETA(a.j[1],b.j[1])

x2 <- new_BETA(a.j[2],b.j[2])

x3 <- new_BETA(a.j[3],b.j[3])

x4 <- new_BETA(a.j[4],b.j[4])

x5 <- new_BETA(a.j[5],b.j[5])

x6 <- new_BETA(a.j[6],b.j[6])

x7 <- new_BETA(a.j[7],b.j[7])

x8 <- new_BETA(a.j[8],b.j[8])

xnew <- new_MIXTURE(list(x1,x2,x3,x4,x5,x6,x7,x8),c(mod.weight[,j]))

## empirical probability density function

xx <- rfunc(xnew, 10000)

xxdf <- tidyr::gather(data.frame(xx), dimension, value)

epdf.den <- density(xxdf$value)

epdf <- matrix(NA,nrow = length(epdf.den$y), ncol = 2, byrow = T)

epdf[,1] <- epdf.den$x

epdf[,2] <- epdf.den$y

## find the strongest key

# order.max <- which(m$y==max(m$y),arr.ind=TRUE)

# xmax <- m$x[order.max]

## empirical cumulative distribution function

x <- sort(xxdf$value)

n <- length(x)

xaxis <- unique(x)

yaxis <- cumsum(tabulate(match(x, xaxis)))/n

ecdf <- matrix(NA,nrow = length(xaxis), ncol = 2, byrow = T)

ecdf[,1] <- xaxis

ecdf[,2] <- yaxis

## calc the density

keys.cdf <- matrix(NA,nrow = length(keys), ncol = 1, byrow = T)

for (p in 1:length(keys)){

xp <- abs(ecdf[,1]-keys[p])

order.min <- which(xp ==min(xp),arr.ind=TRUE)

keys.cdf[p,1] <- ecdf[,2][order.min]

}

for (m in 1:(length(keys)-1)){

mem.post[j,m] <- as.numeric(keys.cdf[m+1,1] - keys.cdf[m,1])

}

}

return(mem.post)

}

}

return(posterior.prob(y.d,

n.d,

a.d,

b.d,

a = 1,

b = 1,

mod.mtx=NULL,

mod.weight=NULL))

}

select.mtd.kb <- function(target, npts, ntox, cutoff.eli = 0.95,

extrasafe = FALSE, offset = 0.05) {

## isotonic transformation using the pool adjacent violator algorithm (PAVA)

pava <- function(x, wt = rep(1, length(x))) {

n <- length(x)

if (n <= 1) {

return(x)

}

if (any(is.na(x)) || any(is.na(wt))) {

stop("Missing values in 'x' or 'wt' not allowed")

}

lvlsets <- (1:n)

repeat {

viol <- (as.vector(diff(x)) < 0)

if (!(any(viol))) {

break

}

i <- min((1:(n - 1))[viol])

lvl1 <- lvlsets[i]

lvl2 <- lvlsets[i + 1]

ilvl <- (lvlsets == lvl1 | lvlsets == lvl2)

x[ilvl] <- sum(x[ilvl] * wt[ilvl])/sum(wt[ilvl])

lvlsets[ilvl] <- lvl1

}

x # ?? printing x ??

}

## determine whether the dose has been eliminated during the trial

y = ntox

n = npts

ndose = length(n)

elimi = rep(0, ndose)

for (i in 1:ndose) {

if (n[i] >= 3) {

if (1 - pbeta(target, y[i] + 1, n[i] - y[i] + 1) > cutoff.eli) {

elimi[i:ndose] = 1

break

}

}

}

if (extrasafe) {

if (n[1] >= 3) {

if (1 - pbeta(target, y[1] + 1, n[1] - y[1] + 1) > cutoff.eli - offset) {

elimi[1:ndose] = 1

}

}

}

## no dose should be selected (i.e., selectdose=99) if the first dose is already very toxic

## or all uneliminated doses are never used to treat patients

if (elimi[1] == 1 || sum(n[elimi == 0]) == 0) {

selectdose = 99

}

else {

adm.set = (n != 0) & (elimi == 0)

adm.index = which(adm.set == T)

y.adm = y[adm.set]

n.adm = n[adm.set]

## poster mean and variance of toxicity probabilities using beta(0.05, 0.05) as the prior

phat = (y.adm + 0.05)/(n.adm + 0.1)

phat.var = (y.adm + 0.05) * (n.adm - y.adm + 0.05)/((n.adm + 0.1)^2 * (n.adm + 0.1 + 1))

## perform the isotonic transformation using PAVA

phat = pava(phat, wt = 1/phat.var)

phat = phat + (1:length(phat)) * 1e-10 ## break ties by adding an increasingly small number

selectd = sort(abs(phat - target), index.return = T)$ix[1] ## select dose closest to the target as the MTD

selectdose = adm.index[selectd]

}

# old: if (verbose == TRUE) {remaining of function}:

trtd = (n != 0)

poverdose = pava(1 - pbeta(target, y[trtd] + 0.05, n[trtd] - y[trtd] + 0.05))

phat.all = pava((y[trtd] + 0.05)/(n[trtd] + 0.1),

wt = 1/((y[trtd] + 0.05) * (n[trtd] - y[trtd] + 0.05)/((n[trtd] + 0.1)^2 * (n[trtd] + 0.1 + 1))))

A1 = A2 = A3 = A4 = NULL

k=1

## output summary statistics

for (i in 1:ndose) {

if (n[i] > 0) {

A1 = append(A1, formatC(phat.all[k], digits = 2, format = "f"))

A2 = append(A2, formatC(qbeta(0.025, y[i] + 0.05, n[i] - y[i] + 0.05), digits = 2, format = "f"))

A3 = append(A3, formatC(qbeta(0.975, y[i] + 0.05, n[i] - y[i] + 0.05), digits = 2, format = "f"))

A4 = append(A4, formatC(poverdose[k], digits = 2, format = "f"))

k = k+1

}

else {

# no estimate output for doses never used to treat patients

A1 = append(A1, "----")

A2 = append(A2, "----")

A3 = append(A3, "----")

A4 = append(A4, "----")

}

}

p_est = data.frame(cbind('dose'=1:length(npts), 'phat'=A1, 'CI'=paste("(", A2,",", A3,")",sep="")))

out = list(target = target, MTD = selectdose, p_est = p_est, p_overdose = A4)

return(out)

}

get.boundary.kb <- function(target, ncohort, cohortsize, marginL=0.05, marginR=0.05,

cutoff.eli=0.95, n.earlystop=100, extrasafe=FALSE, offset=0.05) {

### simple error checking

if(target<0.05) {warning("Error: the target is too low! \n"); return();}

if(target>0.6) {warning("Error: the target is too high! \n"); return();}

if(offset>=0.5) {warning("Error: the offset is too large! \n"); return();}

if(n.earlystop<=6) {warning("Warning: the value of n.earlystop is too low to ensure good operating characteristics. Recommend n.earlystop = 9 to 18 \n"); return();}

## get cutoffs for keys

getkey <- function(a1, a2)

{

delta=a2-a1

lkey=NULL; rkey=NULL

i=0; cutoff=0.3

while (cutoff>0)

{

i=i+1

cutoff = a1-i*delta

lkey = c(cutoff, lkey)

}

lkey[lkey<0]=0

i=0; cutoff=0.3

while (cutoff<1)

{

i=i+1

cutoff = a2+i*delta

rkey = c(rkey, cutoff)

}

rkey[rkey>1]=1

key=c(lkey, a1, a2, rkey)

return(key)

}

## Identify the key with the highest posterior to cutoff.elicity probability

keys = getkey(target-marginL, target+marginR)

nkeys = length(keys)-1

targetp = NULL

npts = ncohort*cohortsize

a=1; b=1; # hyperparameters used in beta prior

decision_table = matrix(NA,nrow=npts+1,ncol=npts)

for (ntr in 1:npts)

{

elim=0

for (ntox in 0:ntr)

{

if (1-pbeta(target, ntox+a, ntr-ntox+b)>cutoff.eli) {elim=1; break;}

for (i in 1:nkeys)

{

comp = 1;

if (i==1 || i==nkeys) {

comp = (marginL+marginR)/(keys[i+1]-keys[i]) #compensation factor for incompleted keys

}

targetp[i] = (pbeta(keys[i+1], ntox+a, ntr-ntox+b) - pbeta(keys[i], ntox+a, ntr-ntox+b))*comp

}

highkey = max(which(targetp==max(targetp)))

targetkey = which(keys==(target-marginL))

if (highkey>targetkey) {decision_table[ntox+1,ntr] <- "D"}

if (highkey==targetkey) {decision_table[ntox+1,ntr] <- "S"}

if (highkey<targetkey) {decision_table[ntox+1,ntr] <- "E"}

}

if (elim==1) {

decision_table[(ntox+1):(ntr+1),ntr] <- rep("DU",ntr-ntox+1)

}

}

colnames(decision_table) <- 1:npts

rownames(decision_table) <- 0:npts

boundary = matrix(NA, nrow=4, ncol=npts)

boundary[1,] = 1:npts

for (i in 1:npts)

{

if (length(which(decision_table[,i]=="E"))) {

boundary[2,i] = max(which(decision_table[,i]=="E"))-1

}

else {

boundary[2,i] = -1

}

if (length(which(decision_table[,i]=="D"))) {

boundary[3,i] = min(which(decision_table[,i]=="D"))-1

}

else if (length(which(decision_table[,i]=="DU"))) {

boundary[3,i] = min(which(decision_table[,i]=="DU"))-1

}

if (length(which(decision_table[,i]=="DU"))) {

boundary[4,i] = min(which(decision_table[,i]=="DU"))-1

}

}

colnames(boundary) <- c(rep("", npts))

rownames(boundary) <- c("Number of patients treated",

"Escalate if # of DLT <=",

"de-escalate if # of DLT >=",

"Eliminate if # of DLT >=")

out = list( boundary_tab=boundary[, (1:floor(min(npts, n.earlystop)/cohortsize))*cohortsize],

full_boundary_tab=boundary)

## if extrasafe, add more info, like in BOIN function

if (extrasafe){

stopbd=NULL;

ntrt=NULL;

for ( n in 1:npts){

ntrt = c(ntrt, n);

if (n <3){ stopbd = c(stopbd, NA);}

else{

for (ntox in 1:n){

if (1-pbeta(target, ntox+1, n-ntox+1)>cutoff.eli-offset) {stopneed=1; break;}

}

if (stopneed==1) {stopbd=c(stopbd, ntox);} else (stopbd=c(stopbd,NA))

}

}

stopboundary = rbind(ntrt, stopbd)[, 1:min(npts, n.earlystop)]

rownames(stopboundary) = c("The number of patients treated at the lowest dose ", "Stop the trial if # of DLT >= ");

colnames(stopboundary) = rep("", min(npts, n.earlystop));

out = c(out,list(target=target, cutoff=cutoff.eli-offset, stop_boundary=stopboundary))

}

return(out)

}

get.oc.mem.kb <- function (target, p.true, ncohort, cohortsize, dist1=1, dist2=1, accrual = 2,

alpha=0.5, maxt = 3, n.earlystop = 100, startdose = 1, type = 1,

marginL = 0.05, marginR = 0.05, cutoff.eli = 0.95,

extrasafe = FALSE, offset = 0.05, ntrial = 1000) {

gen.tite<-function(dist=1, n, pi, alpha=0.5, Tobs=1)

{

############ subroutines ############

weib<-function(n, pi, pihalft)

{

## solve parameters for Weibull given pi=1-S(T) and phalft=1-S(T/2)

alpha = log(log(1-pi)/log(1-pihalft))/log(2);

lambda = -log(1-pi)/(Tobs^alpha);

t = (-log(runif(n))/lambda)^(1/alpha);

return(t);

}

llogit<-function(n, pi, pihalft)

{

## solve parameters for log-logistic given pi=1-S(T) and phalft=1-S(T/2)

alpha = log((1/(1-pi)-1)/(1/(1-pihalft)-1))/log(2);

lambda = (1/(1-pi)-1)/(Tobs^alpha);

t = ((1/runif(n)-1)/lambda)^(1/alpha);

return(t);

}

############ end of subroutines ############

tox = rep(0, n);

t.tox = rep(0, n);

#### uniform

if(dist==1) { # 50% event in (0, 1/2T)

tox = rbinom(n, 1, pi);

ntox.st = sum(tox);

t.tox[tox==0]=Tobs;

t.tox[tox==1]=runif(ntox.st, 0, Tobs);

}

#### Weibull

if(dist==2)

{

pihalft = alpha*pi; # alpha*100% event in (0, 1/2T)

t.tox = weib(n, pi, pihalft);

tox[t.tox<=Tobs]=1;

ntox.st = sum(tox);

t.tox[tox==0]=Tobs;

}

#### log-logistic

if(dist==3)

{

pihalft = alpha*pi; # alpha*100% event in (0, 1/2T)

t.tox = llogit(n, pi, pihalft);

tox[t.tox<=Tobs]=1;

ntox.st = sum(tox);

t.tox[tox==0]=Tobs;

}

return(list(tox=tox, t.tox=t.tox, ntox.st=ntox.st));

}

if (offset >= 0.5) {

warning("Error: the offset is too large! \n")

return()

}

if (n.earlystop <= 6) {

warning("Warning: the value of n.earlystop is too low to ensure good operating characteristics.",

"Recommend n.earlystop = 9 to 18 \n")

return()

}

set.seed(seed)

ndose = length(p.true)

npts = ncohort * cohortsize

Y = matrix(rep(0, ndose * ntrial), ncol = ndose)

N = matrix(rep(0, ndose * ntrial), ncol = ndose)

dselect = rep(0, ntrial)

durationV = rep(0, ntrial)

for (trial in 1:ntrial) {

y.current <- matrix(0,nrow = 1, ncol = ndose, byrow = T)

colnames(y.current) <- c(paste0('dose',seq(1,ndose)))

n.current <- matrix(0,nrow = 1, ncol = ndose, byrow = T)

colnames(n.current) <- c(paste0('dose',seq(1,ndose)))

earlystop = 0

j.c <- startdose ## start dose

elimi = rep(0, ndose)

t.enter=NULL

t.event=NULL

t.decision = 0

for (i in 1:ncohort) {

# generate time data for the new patient

for(j in 1:cohortsize)

{

if(j==1) { t.enter = c(t.enter, t.decision); }

else {

if(dist2==1){ t.enter = c(t.enter, t.enter[length(t.enter)] + runif(1, 0, 2/accrual))}

if(dist2==2){ t.enter = c(t.enter, t.enter[length(t.enter)] + rexp(1, rate=accrual))}

}

}

obscohort = gen.tite(dist1, cohortsize, p.true[j.c], alpha=alpha,T=maxt)

t.event = c(t.event, obscohort$t.tox)

t.decision = t.enter[length(t.enter)]

if(i==ncohort) { t.decision = t.decision + maxt; } else {

t.decision = max(t.enter+t.event)

}

y <- sum(obscohort$tox)

c <- cohortsize

y.current[1,j.c] <- y.current[1,j.c] + y

n.current[1,j.c] <- n.current[1,j.c] + c

## stop if npts at dose j.c > 9

if (n.current[1,j.c] >= n.earlystop) {

break

}

temp = get.boundary.kb(target, ncohort, cohortsize)$full_boundary_tab

b.e = temp[2, ]

b.d = temp[3, ]

b.elim = temp[4, ]

if (!is.na(b.elim[n.current[1,j.c]])) {

if (n.current[1,j.c] >= 3 && y.current[1,j.c] >= b.elim[n.current[1,j.c]]) {

elimi[j.c:ndose] = 1

if (j.c == 1) {

earlystop = 1

break

}

}

if (extrasafe) {

if (j.c == 1 && n.current[1,j.c] >= 3) {

if (1 - pbeta(target, y.current[1,j.c] + 1, n.current[1,j.c] - y.current[1,j.c] + 1) > cutoff.eli - offset) {

earlystop = 1

break

}

}

}

}

## dose escalation

if (type == 1){

pj.post <- get.mem.post1(target = target,

half.width = 0.05,

num.h,

ndose,

y.h,

n.h,

y.c = y.current,

n.c = n.current,

a = 1,

b = 1,

type = 1)

}

if (type == 2){

pj.post <- get.mem.post1(target = target,

half.width = 0.05,

num.h,

ndose,

y.h,

n.h,

y.c = y.current,

n.c = n.current,

a = 1,

b = 1,

type = 2)

}

## decision

target.key = 3

## find the strongest key

dose.post <- pj.post[j.c,]

strongest.key <- min(which(dose.post == max(dose.post)))

## judge whether Escalate or De-escalate

if (strongest.key == target.key){

j.next <- j.c

}else if (strongest.key < target.key){

if (j.c == ndose){

j.next <- j.c

}

else {

j.next <- j.c + 1

}

}else {

if (j.c == 1){

j.next <- j.c

}

else {

j.next <- j.c - 1

}

}

j.c <- j.next

}

## select MTD

Y[trial, ] = y.current[1,]

N[trial, ] = n.current[1,]

durationV[trial] = t.decision

## count

if (earlystop == 1) {

dselect[trial] = 99

}

else {

dselect[trial] = select.mtd.kb(target, n.current[1,], y.current[1,])$MTD

}

}

selpercent = rep(0, ndose)

nptsdose = apply(N, 2, mean)

ntoxdose = apply(Y, 2, mean)

for (i in 1:ndose) {

selpercent[i] = sum(dselect == i)/ntrial * 100

}

if (length(which(p.true == target)) > 0) {

nmtd=nptsdose[p.true==target]

pcs=selpercent[p.true==target]

poor_dosing=mean(N[, p.true == target] < npts/ndose) * 100

if (which(p.true==target)== ndose-1) {

overdosing60=mean(N[,p.true>target]>0.6*npts)*100

overdosing70=mean(N[,p.true>target]>0.7*npts)*100

overdosing80=mean(N[,p.true>target]>0.8*npts)*100

}

else {

overdosing60=mean(rowSums(N[,p.true>target])>0.6*npts) * 100

overdosing70=mean(rowSums(N[,p.true>target])>0.7*npts) * 100

overdosing80=mean(rowSums(N[,p.true>target])>0.8*npts) * 100

}

out = list(selpercent=selpercent, npatients=nptsdose, ntox=ntoxdose,

totaltox=sum(Y)/ntrial, totaln=sum(N)/ntrial, percentstop=sum(dselect == 99)/ntrial*100,

overdose60=overdosing60, overdose80=overdosing80, duration=mean(durationV),

poorallocation=mean(N[, p.true==target]<npts/ndose)*100,

simu.setup=data.frame(target=target, p.true=p.true, ncohort=ncohort,

cohortsize = cohortsize, startdose = startdose,

marginL = marginL, marginR = marginR,

cutoff.eli = cutoff.eli, extrasafe = extrasafe, offset = offset,

ntrial = ntrial, dose=1:ndose));

}

else {

out = list(selpercent=selpercent, npatients=nptsdose, ntox=ntoxdose,

totaltox=sum(Y)/ntrial, totaln=sum(N)/ntrial,

percentstop=sum(dselect == 99)/ntrial*100, duration=mean(durationV),

simu.setup=data.frame(target=target, p.true=p.true, ncohort=ncohort,

cohortsize = cohortsize, startdose = startdose,

marginL = marginL, marginR = marginR,

cutoff.eli = cutoff.eli, extrasafe = extrasafe, offset = offset,

ntrial = ntrial, dose=1:ndose));

}

return(out)

}

summary.kb <- function(object, ...) {

A1 = A2 = A3 = A4 = A5 = A6 = A7 = NULL

if (!is.null(object$percentstop)) {

cat("selection percentage at each dose level (%):\n")

cat(formatC(object$selpercent, digits=1, format="f"), sep=" ", "\n")

A1 = append(A1,formatC(object$selpercent, digits=1, format="f"))

cat("average number of patients treated at each dose level:\n")

cat(formatC(object$npatients, digits=1, format="f"), sep =" ", "\n")

A2 = append(A2,formatC(object$npatients, digits=1, format="f"))

cat("average number of toxicity observed at each dose level:\n")

cat(formatC(object$ntox, digits=1, format="f"), sep =" ", "\n")

cat("average number of toxicities:",

formatC(object$totaltox, digits=1, format="f"), "\n")

cat("average number of patients:",

formatC(object$totaln, digits=1, format="f"), "\n")

A3 = append(A3,formatC(object$totaln, digits=1, format="f"))

cat("percentage of early stopping due to toxicity:",

formatC(object$percentstop, digits=1, format="f"), "\b% \n")

A4 = append(A4,formatC(object$percentstop, digits=1, format="f"))

if (!is.null(object$duration)) {

cat("duration of the trial:",

formatC(object$duration, digits=1, format="f"), "\n")

A7 = append(A7,formatC(object$duration, digits=1, format="f"))

}

if (!is.null(object$overdose60)) {

cat("risk of poor allocation:",

formatC(object$poorallocation, digits=1, format="f"), "% \n")

A5 = append(A5,formatC(object$poorallocation, digits=1, format="f"))

cat("risk of overdosing (>60% of patients treated above the MTD):",

formatC(object$overdose60, digits=1, format="f"), "\b% \n")

A6 = append(A6,formatC(object$overdose60, digits=1, format="f"))

cat("risk of overdosing (>80% of patients treated above the MTD):",

formatC(object$overdose80, digits=1, format="f"), "\b% \n")

}

}

p_est = data.frame(rbind('selection'=A1, 'pts at MTD'=A2))

outp = list(p_est = p_est, Duration = A7, Stop = A4, Overdose = A6,

risk_of_poor_allocation = A5, Sample_size = A3)

return(outp)

}

# ncohort <- 8

# cohortsize <- 3

#

# ndose <- 4

# ## length(p.true) = totaldose

# target <- 0.3

#

# num.h <- 3

#

# p.true <- c(0.28,0.41,0.52,0.63)

#

# target = 0.28

# ncohort = 8

# cohortsize = 3

# num.h=3

#

# y.h <- matrix(c(0,0,0,0,

# 0,0,0,0,

# 0,0,0,0)

# ,nrow = 3, ncol = 4, byrow = T)

#

# n.h <- matrix(c(0,0,0,0,

# 0,0,0,0,

# 0,0,0,0)

# ,nrow = 3, ncol = 4, byrow = T)

#

# oc.mem.kb1 <- get.oc.mem.kb(target = target, p.true = p.true,

# ncohort = ncohort, cohortsize = cohortsize,

# n.earlystop = 100, type = 1, cutoff.eli = 0.90,

# extrasafe = FALSE, offset = 0.05, ntrial = 1000)
