# Supplementary material for: Enhancing phase I dose-finding trials design through dynamic borrowing information and handling late-onset toxicity
Source: Front Pharmacol. 2023 Nov 22;14:1266322. doi: 10.3389/fphar.2023.1266322 (PMC10703472; doi:10.3389/fphar.2023.1266322)

Supplementary Material

# Description for Supplementary Material

This supplementary material consists of the supplementary tables and figures mentioned in the manuscript. Table S1 and Table S2 provides decision tables of MEM-Keyboard with different historical data. And Table S3 and Table S4 provides decision tables of MEM-TITE-Keyboard with different historical data. All the decision tables are produced by approximated likelihood (2.5 and 2.15). For the sensitive analysis, Table S5, Table S6 and Table S7 show the impact of prior specification on the design. Figure S1 shows the similarity of the performance between TITE-Keyboard and TITE-Keyboard (E) designs, and MEM-TITE-Keyboard and MEM-TITE-Keyboard (E) designs.

# Supplementary Tables and Figures

## Table S1 Dose-escalation and de-escalation boundaries for MEM-Keyboard with a target DLT rate of 0.28 and cohort size of 3 up to 12 patients (historical DLT rate for current dose level is 3/8, 3/5, and 3/7)

| Number of patients treated | Escalate if number of DLT <= | | De-escalate if number of DLT >= | Eliminate if number of DLT >= |
| --- | --- | --- | --- | --- |
| 1 | -1 | 1 | | NA |
| 2 | 0 | 1 | | 2 |
| 3 | 0 | 1 | | 3 |
| 4 | 0 | 1 | | 3 |
| 5 | 0 | 2 | | 4 |
| 6 | 0 | 2 | | 4 |
| 7 | 1 | 2 | | 4 |
| 8 | 1 | 3 | | 5 |
| 9 | 1 | 3 | | 5 |
| 10 | 1 | 3 | | 6 |
| 11 | 2 | 4 | | 6 |
| 12 | 2 | 4 | | 6 |

Table S1**.** When the historical data is given as follows: 3/8, 3/5, and 3/7. Assuming a target DLT rate of 0.28 and cohort size of 3 up to 24 patients, Table S1 shows the decision tables for MEM-Keyboard. A conclusion can be drawn that when the historical data suggests a higher toxicity level for a certain dose, MEM-Keyboard is more prone to dose reduction or dose maintenance, with more stringent rules for dose escalation.

## Table S2. Dose-escalation and de-escalation boundaries for MEM-Keyboard with a target DLT rate of 0.28 and cohort size of 3 up to 12 patients (all historical DLT rate for current dose level is 0/0, namely no historical data available)

| Number of patients treated | Escalate if number of DLT <= | De-escalate if number of DLT >= | Eliminate if number of DLT >= |
| --- | --- | --- | --- |
| 1 | 0 | 1 | NA |
| 2 | 0 | 1 | 2 |
| 3 | 0 | 1 | 3 |
| 4 | 0 | 2 | 3 |
| 5 | 1 | 2 | 4 |
| 6 | 1 | 2 | 4 |
| 7 | 1 | 3 | 4 |
| 8 | 1 | 3 | 5 |
| 9 | 2 | 3 | 5 |
| 10 | 2 | 4 | 6 |
| 11 | 2 | 4 | 6 |
| 12 | 2 | 4 | 6 |

Table S2. When the historical data is given as follows: 0/0, 0/0, and 0/0. Assuming a target DLT rate of 0.28 and cohort size of 3 up to 24 patients, Table S2 shows the decision tables for MEM-Keyboard. The decision table for MEM-Keyboard remain consistent with that of Keyboard.

## Table S3. Dose-escalation and de-escalation boundaries for MEM-TITE-Keyboard with a target DLT rate of 0.28 and cohort size of 3 up to 12 patients (historical DLT rate for current dose level is 3/8, 3/5, and 3/7)

| Number of patients treated | Number of observed DLTs | Number of pending patients | Escalation | Stay | De-escalation |
| --- | --- | --- | --- | --- | --- |
| 3 | 0 | < 2 | Yes |  |  |
| 3 | 0 | > 1 |  | Suspend |  |
| 3 | 1, 2 | < 3 |  |  | Yes |
| 3 | 3 | 0 |  |  | Yes & Eliminate |
| 6 | 0 | < 5 | Yes |  |  |
| 6 | 0 | 5 ~ 6 | ESS> 1.21 | ESS in [1.02,1.21] | ESS< 1.02 |
| 6 | 1 | 0 | Yes |  |  |
| 6 | 1 | 1 | ESS> 5.66 | ESS<= 5.66 |  |
| 6 | 1 | 2 ~ 5 | ESS> 5.66 | ESS in [4.08,5.66] | ESS< 4.08 |
| 6 | 2, 3 | < 5 |  |  | Yes |
| 6 | > 3 | < 3 |  |  | Yes & Eliminate |
| 9 | 0 | < 8 | Yes |  |  |
| 9 | 0 | 8 ~ 9 | ESS> 1.21 | ESS in [1.02,1.21] | ESS< 1.02 |

**Table S3. (Continued)**

| Number of patients treated | Number of observed DLTs | Number of pending patients | Escalation | Stay | De-escalation |
| --- | --- | --- | --- | --- | --- |
| 9 | 1 | < 4 | Yes |  |  |
| 9 | 1 | 4 | ESS> 5.66 | ESS<= 5.66 |  |
| 9 | 1 | 5 ~ 8 | ESS> 5.66 | ESS in [4.08,5.66] | ESS< 4.08 |
| 9 | 2 | < 2 |  | Yes |  |
| 9 | 2 | 2 ~ 7 |  | ESS>= 7.13 | ESS< 7.13 |
| 9 | 3, 4 | < 7 |  |  | Yes |
| 9 | > 4 | < 5 |  |  | Yes & Eliminate |
| 12 | 0 | < 11 | Yes |  |  |
| 12 | 0 | 11 ~ 12 | ESS> 1.21 | ESS in [1.02,1.21] | ESS< 1.02 |
| 12 | 1 | < 7 | Yes |  |  |
| 12 | 1 | 7 | ESS> 5.66 | ESS<= 5.66 |  |
| 12 | 1 | 8 ~ 11 | ESS> 5.66 | ESS in [4.08,5.66] | ESS< 4.08 |
| 12 | 2 | < 2 | Yes |  |  |
| 12 | 2 | 2 ~ 4 | ESS> 10.1 | ESS<= 10.1 |  |

**Table S3. (Continued)**

| Number of patients treated | Number of observed DLTs | Number of pending patients | Escalation | Stay | De-escalation |
| --- | --- | --- | --- | --- | --- |
| 12 | 2 | 5 ~ 10 | ESS> 10.1 | ESS in [7.13,10.1] | ESS< 7.13 |
| 12 | 3 | < 2 |  | Yes |  |
| 12 | 3 | 2 ~ 9 |  | ESS>= 10.19 | ESS< 10.19 |
| 12 | 4, 5 | < 9 |  |  | Yes |
| 12 | > 5 | < 7 |  |  | Yes & Eliminate |

ESS *_j_* = No. of non-pending patients at dose *j* +$\frac{Total follow-up time for pending patients at dose j}{Length of assessment window}$

From Table S4, the same conclusion as that of Table S1 can be drawn that when the historical data suggests a higher toxicity level for a certain dose, MEM-TITE-Keyboard is more prone to dose reduction or dose maintenance, with more stringent rules for dose escalation.

## Table S4. Dose-escalation and de-escalation boundaries for MEM-TITE-Keyboard with a target DLT rate of 0.28 and cohort size of 3 up to 12 patients (all historical DLT rate for current dose level is 0/0, namely no historical data available)

| Number of patients treated | Number of observed DLTs | Number of pending patients | Ecalation | Stay | De-escalation |
| --- | --- | --- | --- | --- | --- |
| 3 | 0 | < 2 | Yes |  |  |
| 3 | 0 | > 1 |  | Suspend |  |
| 3 | 1, 2 | < 3 |  |  | Yes |
| 3 | 3 | 0 |  |  | Yes & Eliminate |
| 6 | 0 | < 7 | Yes |  |  |
| 6 | 1 | < 2 | Yes |  |  |
| 6 | 1 | 2 | ESS> 4.45 | ESS<= 4.45 |  |
| 6 | 1 | 3 ~ 5 | ESS> 4.45 | ESS in [3.05,4.45] | ESS< 3.05 |
| 6 | 2, 3 | < 5 |  |  | Yes |
| 6 | > 3 | < 3 |  |  | Yes & Eliminate |
| 9 | 0 | < 10 | Yes |  |  |
| 9 | 1 | < 5 | Yes |  |  |
| 9 | 1 | 5 | ESS> 4.45 | ESS<= 4.45 |  |
| 9 | 1 | 6 ~ 8 | ESS> 4.45 | ESS in [3.05,4.45] | ESS< 3.05 |
| 9 | 2 | 0 | Yes |  |  |

**Table S4. (Continued)**

| Number of patients treated | Number of observed DLTs | Number of pending patients | Ecalation | Stay | De-escalation |
| --- | --- | --- | --- | --- | --- |
| 9 | 2 | 1 ~ 2 | ESS> 8.9 | ESS<= 8.9 |  |
| 9 | 2 | 3 ~ 7 | ESS> 8.9 | ESS in [6.11,8.9] | ESS< 6.11 |
| 9 | 3, 4 | < 7 |  |  | Yes |
| 9 | > 4 | < 5 |  |  | Yes & Eliminate |
| 12 | 0 | < 13 | Yes |  |  |
| 12 | 1 | < 8 | Yes |  |  |
| 12 | 1 | 8 | ESS> 4.45 | ESS<= 4.45 |  |
| 12 | 1 | 9 ~ 11 | ESS> 4.45 | ESS in [3.05,4.45] | ESS< 3.05 |
| 12 | 2 | < 4 | Yes |  |  |
| 12 | 2 | 4 ~ 5 | ESS> 8.9 | ESS<= 8.9 |  |
| 12 | 2 | 6 ~ 10 | ESS> 8.9 | ESS in [6.11,8.9] | ESS< 6.11 |
| 12 | 3 | < 3 |  | Yes |  |
| 12 | 3 | 3 ~ 9 |  | ESS>= 9.16 | ESS< 9.16 |
| 12 | 4, 5 | < 9 |  |  | Yes |
| 12 | > 5 | < 7 |  |  | Yes & Eliminate |

ESS *_j_* = No. of non-pending patients at dose *j* +$\frac{Total follow-up time for pending patients at dose j}{Length of assessment window}$

From Table S4, the same conclusion as that of Table S2 can be drawn that the decision table for MEM-TITE-Keyboard remain consistent with that of TITE-Keyboard when there is no historical data available.

## Table S5. Simulation results with sample size of 24 and cohort size of 3 (π = 0.05)

| Methods |  | dose level | | | | Duration | Stop (%) | Overdose (%) | Risk of Poor allocation (%) | Sample size |
| --- | --- | --- | --- | --- | --- | --- | --- | --- | --- | --- |
|  |  | 1 | 2 | 3 | 4 |  |  |  |  |  |
| Scenario 1 | p.true | **0.28** | 0.41 | 0.52 | 0.63 |  |  |  |  |  |
| 3+3 | selection (%) | **37.2** | 13.4 | 2.2 | 0.2 | 9.2 | 19.4 | 0.4 | 56.2 | 7.3 |
|  | pts at MTD | **4.3** | 2.3 | 0.6 | 0.1 |  |  |  |  |  |
| Keyboard | selection (%) | **65** | 19.6 | 2.4 | 0.2 | 20.4 | 12.9 | 15 | 6.3 | 16.1 |
|  | pts at MTD | **10.6** | 4.4 | 1 | 0.1 |  |  |  |  |  |
| MEM-Keyboard | selection (%) | **66.9** | 18.2 | 1.5 | 0.1 | 19.5 | 13.3 | 11.8 | 6.3 | 15.4 |
|  | pts at MTD | **10.7** | 3.8 | 0.8 | 0.1 |  |  |  |  |  |
| TITE-Keyboard | selection (%) | **66** | 18.8 | 2.1 | 0.1 | 12.5 | 12.9 | 14 | 8.2 | 15.2 |
|  | pts at MTD | **10.3** | 4 | 0.9 | 0.1 |  |  |  |  |  |
| TITE-Keyboard (E) | selection (%) | **66.7** | 18.8 | 1.8 | 0.1 | 12.4 | 12.6 | 13.4 | 8.4 | 15.2 |
|  | pts at MTD | **10.3** | 3.9 | 0.9 | 0.1 |  |  |  |  |  |
| MEM-TITE-Keyboard | selection (%) | **67.7** | 17.8 | 1.6 | 0.1 | 11.6 | 12.8 | 10.4 | 6 | 14.8 |
|  | pts at MTD | **10.6** | 3.5 | 0.6 | 0.1 |  |  |  |  |  |
| MEM-TITE-Keyboard (E) | selection (%) | **67.8** | 17.7 | 1.6 | 0.1 | 11.6 | 12.8 | 10.4 | 6 | 14.8 |
|  | pts at MTD | **10.7** | 3.5 | 0.6 | 0.1 |  |  |  |  |  |

**Table S5. (Continued)**

| Methods |  | dose level | | | | Duration | Stop (%) | Overdose (%) | Risk of Poor allocation (%) | Sample size |
| --- | --- | --- | --- | --- | --- | --- | --- | --- | --- | --- |
|  |  | 1 | 2 | 3 | 4 |  |  |  |  |  |
| Scenario 2 | p.true | 0.14 | **0.28** | 0.41 | 0.52 |  |  |  |  |  |
| 3+3 | selection (%) | 38.9 | **31.5** | 11 | 1.7 | 12.7 | 5.5 | 0 | 64.1 | 9.9 |
|  | pts at MTD | 3.9 | **3.6** | 1.9 | 0.5 |  |  |  |  |  |
| Keyboard | selection (%) | 28 | **51.1** | 16.6 | 3.3 | 26.7 | 0.9 | 5.7 | 20.6 | 20.7 |
|  | pts at MTD | 8.8 | **7.8** | 3.4 | 0.7 |  |  |  |  |  |
| MEM-Keyboard | selection (%) | 27.6 | **54.3** | 14.9 | 2.5 | 26.6 | 0.7 | 5.7 | 19.2 | 20.6 |
|  | pts at MTD | 8.8 | **8.1** | 3.1 | 0.6 |  |  |  |  |  |
| TITE-Keyboard | selection (%) | 33.7 | **49** | 14.5 | 2 | 15.1 | 0.9 | 6.7 | 35.8 | 19 |
|  | pts at MTD | 8.5 | **7.1** | 2.8 | 0.6 |  |  |  |  |  |
| TITE-Keyboard (E) | selection (%) | 33.6 | **48.8** | 14.8 | 1.9 | 15.1 | 0.9 | 6.6 | 36.4 | 19 |
|  | pts at MTD | 8.6 | **7** | 2.8 | 0.6 |  |  |  |  |  |
| MEM-TITE-Keyboard | selection (%) | 32.7 | **51.3** | 13.8 | 1.5 | 14.6 | 0.8 | 4.6 | 30.9 | 18.9 |
|  | pts at MTD | 8.4 | **7.6** | 2.6 | 0.4 |  |  |  |  |  |
| MEM-TITE-Keyboard (E) | selection (%) | 33.4 | **50.6** | 13.6 | 1.6 | 14.6 | 0.9 | 4.7 | 32.9 | 18.9 |
|  | pts at MTD | 8.6 | **7.4** | 2.5 | 0.4 |  |  |  |  |  |

**Table S5. (Continued)**

| Methods |  | dose level | | | | Duration | Stop (%) | Overdose (%) | Risk of Poor allocation (%) | Sample size |
| --- | --- | --- | --- | --- | --- | --- | --- | --- | --- | --- |
|  |  | 1 | 2 | 3 | 4 |  |  |  |  |  |
| Scenario 3 | p.true | 0.11 | 0.14 | **0.28** | 0.41 |  |  |  |  |  |
| 3+3 | selection (%) | 14.4 | 35.2 | **28.1** | 11.4 | 15.8 | 3.2 | 0 | 67.3 | 12.2 |
|  | pts at MTD | 3.8 | 3.5 | **3.2** | 1.7 |  |  |  |  |  |
| Keyboard | selection (%) | 7.9 | 27.4 | **45.9** | 18.5 | 29.4 | 0.4 | 0 | 27.7 | 22.6 |
|  | pts at MTD | 6.1 | 7.6 | **6.2** | 2.8 |  |  |  |  |  |
| MEM-Keyboard | selection (%) | 5.9 | 25.6 | **50.7** | 17.3 | 29.1 | 0.4 | 0 | 22.9 | 22.4 |
|  | pts at MTD | 4.7 | 8.1 | **6.9** | 2.8 |  |  |  |  |  |
| TITE-Keyboard | selection (%) | 13.7 | 29.4 | **42** | 14.4 | 18.2 | 0.4 | 0 | 42.9 | 21 |
|  | pts at MTD | 6.3 | 6.7 | **5.7** | 2.3 |  |  |  |  |  |
| TITE-Keyboard (E) | selection (%) | 14.6 | 29.6 | **41.2** | 14.1 | 18 | 0.4 | 0 | 44.9 | 20.9 |
|  | pts at MTD | 6.5 | 6.7 | **5.5** | 2.2 |  |  |  |  |  |
| MEM-TITE-Keyboard | selection (%) | 11.9 | 29.4 | **44.6** | 13.8 | 18.1 | 0.4 | 0 | 39.3 | 21.3 |
|  | pts at MTD | 6.1 | 7 | **6.1** | 2 |  |  |  |  |  |
| MEM-TITE-Keyboard (E) | selection (%) | 12.4 | 30.1 | **44.4** | 12.6 | 17.9 | 0.4 | 0 | 41.1 | 21.1 |
|  | pts at MTD | 6.2 | 7 | **5.9** | 1.9 |  |  |  |  |  |

**Table S5. (Continued)**

| Methods |  | dose level | | | | Duration | Stop (%) | Overdose (%) | Risk of Poor allocation (%) | Sample size |
| --- | --- | --- | --- | --- | --- | --- | --- | --- | --- | --- |
|  |  | 1 | 2 | 3 | 4 |  |  |  |  |  |
| Scenario 4 | p.true | 0.03 | 0.11 | 0.14 | **0.28** |  |  |  |  |  |
| 3+3 | selection (%) | 10.7 | 14.9 | 33.9 | **39.5** | 17.9 | 0.4 | 0 | 68.9 | 13.6 |
|  | pts at MTD | 3.2 | 3.8 | 3.5 | **3.1** |  |  |  |  |  |
| Keyboard | selection (%) | 0.7 | 8.6 | 30.2 | **60.5** | 30.7 | 0 | 0 | 34.4 | 23.4 |
|  | pts at MTD | 4.7 | 5.9 | 6.4 | **6.4** |  |  |  |  |  |
| MEM-Keyboard | selection (%) | 0.5 | 6.6 | 29.1 | **63.9** | 30.2 | 0 | 0 | 22 | 23 |
|  | pts at MTD | 3.5 | 4.6 | 7.5 | **7.4** |  |  |  |  |  |
| TITE-Keyboard | selection (%) | 1.6 | 15.3 | 30.8 | **52.3** | 20.8 | 0 | 0 | 46.9 | 22.5 |
|  | pts at MTD | 4.7 | 6.1 | 6.1 | **5.5** |  |  |  |  |  |
| TITE-Keyboard (E) | selection (%) | 1.8 | 15.7 | 31.1 | **51.3** | 20.7 | 0 | 0 | 49 | 22.5 |
|  | pts at MTD | 4.8 | 6.2 | 6.1 | **5.4** |  |  |  |  |  |
| MEM-TITE-Keyboard | selection (%) | 1.2 | 12.4 | 31.5 | **54.8** | 20.9 | 0 | 0 | 40.6 | 22.8 |
|  | pts at MTD | 4.5 | 5.9 | 6.4 | **6** |  |  |  |  |  |
| MEM-TITE-Keyboard (E) | selection (%) | 1.3 | 14 | 31.4 | **53.3** | 20.8 | 0 | 0 | 43.5 | 22.6 |
|  | pts at MTD | 4.6 | 6 | 6.3 | **5.7** |  |  |  |  |  |

**Table S5. (Continued)**

| Methods |  | dose level | | | | Duration | Stop (%) | Overdose (%) | Risk of Poor allocation (%) | Sample size |
| --- | --- | --- | --- | --- | --- | --- | --- | --- | --- | --- |
|  |  | 1 | 2 | 3 | 4 |  |  |  |  |  |
| Scenario 5 | p.true | 0.14 | **0.28** | 0.41 | 0.52 |  |  |  |  |  |
| 3+3 | selection (%) | 38.9 | **31.5** | 11 | 1.7 | 12.7 | 5.5 | 0 | 64.1 | 9.9 |
|  | pts at MTD | 3.9 | **3.6** | 1.9 | 0.5 |  |  |  |  |  |
| Keyboard | selection (%) | 28 | **51.1** | 16.6 | 3.3 | 26.7 | 0.9 | 5.7 | 20.6 | 20.7 |
|  | pts at MTD | 8.8 | **7.8** | 3.4 | 0.7 |  |  |  |  |  |
| MEM-Keyboard | selection (%) | 25.3 | **56** | 15.9 | 2 | 26.5 | 0.8 | 6.9 | 14.4 | 20.6 |
|  | pts at MTD | 7.8 | **8.9** | 3.2 | 0.7 |  |  |  |  |  |
| TITE-Keyboard | selection (%) | 33.7 | **49** | 14.5 | 2 | 15.1 | 0.9 | 6.7 | 35.8 | 19 |
|  | pts at MTD | 8.5 | **7.1** | 2.8 | 0.6 |  |  |  |  |  |
| TITE-Keyboard (E) | selection (%) | 33.6 | **48.8** | 14.8 | 1.9 | 15.1 | 0.9 | 6.6 | 36.4 | 19 |
|  | pts at MTD | 8.6 | **7** | 2.8 | 0.6 |  |  |  |  |  |
| MEM-TITE-Keyboard | selection (%) | 32.2 | **51.4** | 14.1 | 1.5 | 14.6 | 0.8 | 4.6 | 30.8 | 19 |
|  | pts at MTD | 8.4 | **7.6** | 2.6 | 0.4 |  |  |  |  |  |
| MEM-TITE-Keyboard (E) | selection (%) | 33.8 | **50.3** | 13.5 | 1.6 | 14.5 | 0.8 | 4.6 | 32.2 | 18.9 |
|  | pts at MTD | 8.6 | **7.4** | 2.5 | 0.4 |  |  |  |  |  |

**Table S5. (Continued)**

| Methods |  | dose level | | | | Duration | Stop (%) | Overdose (%) | Risk of Poor allocation (%) | Sample size |
| --- | --- | --- | --- | --- | --- | --- | --- | --- | --- | --- |
|  |  | 1 | 2 | 3 | 4 |  |  |  |  |  |
| Scenario 6 | p.true | 0.14 | **0.28** | 0.41 | 0.52 |  |  |  |  |  |
| 3+3 | selection (%) | 38.9 | **31.5** | 11 | 1.7 | 12.7 | 5.5 | 0 | 64.1 | 9.9 |
|  | pts at MTD | 3.9 | **3.6** | 1.9 | 0.5 |  |  |  |  |  |
| Keyboard | selection (%) | 28 | **51.1** | 16.6 | 3.3 | 26.7 | 0.9 | 5.7 | 20.6 | 20.7 |
|  | pts at MTD | 8.8 | **7.8** | 3.4 | 0.7 |  |  |  |  |  |
| MEM-Keyboard | selection (%) | 27.6 | **54.3** | 14.9 | 2.5 | 26.6 | 0.7 | 5.7 | 19.2 | 20.6 |
|  | pts at MTD | 8.8 | **8.1** | 3.1 | 0.6 |  |  |  |  |  |
| TITE-Keyboard | selection (%) | 33.7 | **49** | 14.5 | 2 | 15.1 | 0.9 | 6.7 | 35.8 | 19 |
|  | pts at MTD | 8.5 | **7.1** | 2.8 | 0.6 |  |  |  |  |  |
| TITE-Keyboard (E) | selection (%) | 33.6 | **48.8** | 14.8 | 1.9 | 15.1 | 0.9 | 6.6 | 36.4 | 19 |
|  | pts at MTD | 8.6 | **7** | 2.8 | 0.6 |  |  |  |  |  |
| MEM-TITE-Keyboard | selection (%) | 32.4 | **51.3** | 13.9 | 1.6 | 14.6 | 0.8 | 4.7 | 31.7 | 18.9 |
|  | pts at MTD | 8.4 | **7.5** | 2.5 | 0.4 |  |  |  |  |  |
| MEM-TITE-Keyboard (E) | selection (%) | 34.2 | **50.4** | 12.9 | 1.4 | 14.5 | 1 | 4.4 | 33.6 | 18.8 |
|  | pts at MTD | 8.7 | **7.3** | 2.4 | 0.4 |  |  |  |  |  |

**Table S5. (Continued)**

| Methods |  | dose level | | | | Duration | Stop (%) | Overdose (%) | Risk of Poor allocation (%) | Sample size |
| --- | --- | --- | --- | --- | --- | --- | --- | --- | --- | --- |
|  |  | 1 | 2 | 3 | 4 |  |  |  |  |  |
| Scenario 7 | p.true | 0.14 | **0.28** | 0.41 | 0.52 |  |  |  |  |  |
| 3+3 | selection (%) | 38.9 | **31.5** | 11 | 1.7 | 12.7 | 5.5 | 0 | 64.1 | 9.9 |
|  | pts at MTD | 3.9 | **3.6** | 1.9 | 0.5 |  |  |  |  |  |
| Keyboard | selection (%) | 28 | **51.1** | 16.6 | 3.3 | 26.7 | 0.9 | 5.7 | 20.6 | 20.7 |
|  | pts at MTD | 8.8 | **7.8** | 3.4 | 0.7 |  |  |  |  |  |
| MEM-Keyboard | selection (%) | 27.6 | **54.3** | 14.9 | 2.5 | 26.6 | 0.7 | 5.7 | 19.2 | 20.6 |
|  | pts at MTD | 8.8 | **8.1** | 3.1 | 0.6 |  |  |  |  |  |
| TITE-Keyboard | selection (%) | 33.7 | **49** | 14.5 | 2 | 15.1 | 0.9 | 6.7 | 35.8 | 19 |
|  | pts at MTD | 8.5 | **7.1** | 2.8 | 0.6 |  |  |  |  |  |
| TITE-Keyboard (E) | selection (%) | 33.6 | **48.8** | 14.8 | 1.9 | 15.1 | 0.9 | 6.6 | 36.4 | 19 |
|  | pts at MTD | 8.6 | **7** | 2.8 | 0.6 |  |  |  |  |  |
| MEM-TITE-Keyboard | selection (%) | 33.7 | **50** | 13.7 | 1.6 | 14.6 | 0.9 | 4.8 | 33.3 | 18.8 |
|  | pts at MTD | 8.5 | **7.4** | 2.5 | 0.4 |  |  |  |  |  |
| MEM-TITE-Keyboard (E) | selection (%) | 34.3 | **49.8** | 13.3 | 1.6 | 14.6 | 1 | 4.5 | 35 | 18.8 |
|  | pts at MTD | 8.7 | **7.2** | 2.5 | 0.4 |  |  |  |  |  |

**Table S5. (Continued)**

| Methods |  | dose level | | | | Duration | Stop (%) | Overdose (%) | Risk of Poor allocation (%) | Sample size |
| --- | --- | --- | --- | --- | --- | --- | --- | --- | --- | --- |
|  |  | 1 | 2 | 3 | 4 |  |  |  |  |  |
| Scenario 8 | p.true | 0.11 | 0.14 | **0.28** | 0.41 |  |  |  |  |  |
| 3+3 | selection (%) | 14.4 | 35.2 | **28.1** | 11.4 | 15.8 | 3.1 | 0 | 67.3 | 12.2 |
|  | pts at MTD | 3.8 | 3.5 | **3.2** | 1.7 |  |  |  |  |  |
| Keyboard | selection (%) | 7.9 | 27.4 | **45.9** | 18.5 | 29.4 | 0.4 | 0 | 27.7 | 22.6 |
|  | pts at MTD | 6.1 | 7.6 | **6.2** | 2.8 |  |  |  |  |  |
| MEM-Keyboard | selection (%) | 7.6 | 30.3 | **45** | 16.7 | 29.2 | 0.4 | 0 | 30.7 | 22.4 |
|  | pts at MTD | 6.1 | 7.7 | **6.1** | 2.5 |  |  |  |  |  |
| TITE-Keyboard | selection (%) | 13.7 | 29.4 | **42** | 14.4 | 18.2 | 0.4 | 0 | 42.9 | 21 |
|  | pts at MTD | 6.3 | 6.7 | **5.7** | 2.3 |  |  |  |  |  |
| TITE-Keyboard (E) | selection (%) | 14.6 | 29.6 | **41.2** | 14.1 | 18 | 0.4 | 0 | 44.9 | 20.9 |
|  | pts at MTD | 6.5 | 6.7 | **5.5** | 2.2 |  |  |  |  |  |
| MEM-TITE-Keyboard | selection (%) | 13.2 | 31.8 | **41.9** | 12.8 | 17.3 | 0.3 | 0 | 46.4 | 20.8 |
|  | pts at MTD | 6.3 | 7.4 | **5.2** | 1.8 |  |  |  |  |  |
| MEM-TITE-Keyboard (E) | selection (%) | 13.9 | 32.4 | **41.3** | 12 | 17.2 | 0.3 | 0 | 46.9 | 20.7 |
|  | pts at MTD | 6.5 | 7.4 | **5.1** | 1.7 |  |  |  |  |  |

**Table S5. (Continued)**

| Methods |  | dose level | | | | Duration | Stop (%) | Overdose (%) | Risk of Poor allocation (%) | Sample size |
| --- | --- | --- | --- | --- | --- | --- | --- | --- | --- | --- |
|  |  | 1 | 2 | 3 | 4 |  |  |  |  |  |
| Scenario 9 | p.true | 0.11 | 0.14 | **0.28** | 0.41 |  |  |  |  |  |
| 3+3 | selection (%) | 14.4 | 35.2 | **28.1** | 11.4 | 15.8 | 3.1 | 0 | 67.3 | 12.2 |
|  | pts at MTD | 3.8 | 3.5 | **3.2** | 1.7 |  |  |  |  |  |
| Keyboard | selection (%) | 7.9 | 27.4 | **45.9** | 18.5 | 29.4 | 0.4 | 0 | 27.7 | 22.6 |
|  | pts at MTD | 6.1 | 7.6 | **6.2** | 2.8 |  |  |  |  |  |
| MEM-Keyboard | selection (%) | 5.9 | 22.7 | **51.6** | 19.5 | 28.8 | 0.3 | 0 | 18.1 | 22.2 |
|  | pts at MTD | 4.7 | 6 | **8.1** | 3.5 |  |  |  |  |  |
| TITE-Keyboard | selection (%) | 13.7 | 29.4 | **42** | 14.4 | 18.2 | 0.4 | 0 | 42.9 | 21 |
|  | pts at MTD | 6.3 | 6.7 | **5.7** | 2.3 |  |  |  |  |  |
| TITE-Keyboard (E) | selection (%) | 14.6 | 29.6 | **41.2** | 14.1 | 18 | 0.4 | 0 | 44.9 | 20.9 |
|  | pts at MTD | 6.5 | 6.7 | **5.5** | 2.2 |  |  |  |  |  |
| MEM-TITE-Keyboard | selection (%) | 11.7 | 28.8 | **43.8** | 15.4 | 18.6 | 0.4 | 0 | 38.4 | 21.5 |
|  | pts at MTD | 6.1 | 6.9 | **6.1** | 2.5 |  |  |  |  |  |
| MEM-TITE-Keyboard (E) | selection (%) | 12.2 | 29 | **44.1** | 14.3 | 18.4 | 0.3 | 0 | 40.2 | 21.3 |
|  | pts at MTD | 6.2 | 6.9 | **5.9** | 2.3 |  |  |  |  |  |

**Table S5. (Continued)**

| Methods |  | dose level | | | | Duration | Stop (%) | Overdose (%) | Risk of Poor allocation (%) | Sample size |
| --- | --- | --- | --- | --- | --- | --- | --- | --- | --- | --- |
|  |  | 1 | 2 | 3 | 4 |  |  |  |  |  |
| Scenario 10 | p.true | 0.11 | 0.14 | **0.28** | 0.41 |  |  |  |  |  |
| 3+3 | selection (%) | 14.4 | 35.2 | **28.1** | 11.4 | 15.8 | 3.1 | 0 | 67.3 | 12.2 |
|  | pts at MTD | 3.8 | 3.5 | **3.2** | 1.7 |  |  |  |  |  |
| Keyboard | selection (%) | 7.9 | 27.4 | **45.9** | 18.5 | 29.4 | 0.4 | 0 | 27.7 | 22.6 |
|  | pts at MTD | 6.1 | 7.6 | **6.2** | 2.8 |  |  |  |  |  |
| MEM-Keyboard | selection (%) | 6.6 | 30.5 | **47** | 15.6 | 29 | 0.3 | 0 | 29.7 | 22.3 |
|  | pts at MTD | 5.3 | 8.1 | **6.3** | 2.6 |  |  |  |  |  |
| TITE-Keyboard | selection (%) | 13.7 | 29.4 | **42** | 14.4 | 18.2 | 0.4 | 0 | 42.9 | 21 |
|  | pts at MTD | 6.3 | 6.7 | **5.7** | 2.3 |  |  |  |  |  |
| TITE-Keyboard (E) | selection (%) | 14.6 | 29.6 | **41.2** | 14.1 | 18 | 0.4 | 0 | 44.9 | 20.9 |
|  | pts at MTD | 6.5 | 6.7 | **5.5** | 2.2 |  |  |  |  |  |
| MEM-TITE-Keyboard | selection (%) | 13.6 | 32 | **41.6** | 12.5 | 17.2 | 0.3 | 0 | 46.7 | 20.7 |
|  | pts at MTD | 6.4 | 7.5 | **5.2** | 1.7 |  |  |  |  |  |
| MEM-TITE-Keyboard (E) | selection (%) | 14.3 | 32.2 | **40.9** | 12.4 | 17.2 | 0.2 | 0 | 47.1 | 20.7 |
|  | pts at MTD | 6.5 | 7.4 | **5.1** | 1.7 |  |  |  |  |  |

**Table S5. (Continued)**

| Methods |  | dose level | | | | Duration | Stop (%) | Overdose (%) | Risk of Poor allocation (%) | Sample size |
| --- | --- | --- | --- | --- | --- | --- | --- | --- | --- | --- |
|  |  | 1 | 2 | 3 | 4 |  |  |  |  |  |
| Scenario 11 | p.true | 0.11 | 0.14 | **0.28** | 0.41 |  |  |  |  |  |
| 3+3 | selection (%) | 14.4 | 35.2 | **28.1** | 11.4 | 15.8 | 3.1 | 0 | 67.3 | 12.2 |
|  | pts at MTD | 3.8 | 3.5 | **3.2** | 1.7 |  |  |  |  |  |
| Keyboard | selection (%) | 7.9 | 27.4 | **45.9** | 18.5 | 29.4 | 0.4 | 0 | 27.7 | 22.6 |
|  | pts at MTD | 6.1 | 7.6 | **6.2** | 2.8 |  |  |  |  |  |
| MEM-Keyboard | selection (%) | 7.6 | 30.3 | **45** | 16.7 | 29.2 | 0.4 | 0 | 30.7 | 22.4 |
|  | pts at MTD | 6.1 | 7.7 | **6.1** | 2.5 |  |  |  |  |  |
| TITE-Keyboard | selection (%) | 13.7 | 29.4 | **42** | 14.4 | 18.2 | 0.4 | 0 | 42.9 | 21 |
|  | pts at MTD | 6.3 | 6.7 | **5.7** | 2.3 |  |  |  |  |  |
| TITE-Keyboard (E) | selection (%) | 14.6 | 29.6 | **41.2** | 14.1 | 18 | 0.4 | 0 | 44.9 | 20.9 |
|  | pts at MTD | 6.5 | 6.7 | **5.5** | 2.2 |  |  |  |  |  |
| MEM-TITE-Keyboard | selection (%) | 14 | 31.7 | **40.7** | 13.4 | 17.4 | 0.3 | 0 | 47 | 20.8 |
|  | pts at MTD | 6.4 | 7.3 | **5.1** | 1.9 |  |  |  |  |  |
| MEM-TITE-Keyboard (E) | selection (%) | 14.6 | 31.3 | **41** | 12.9 | 17.4 | 0.3 | 0 | 47.8 | 20.7 |
|  | pts at MTD | 6.5 | 7.3 | **5.1** | 1.8 |  |  |  |  |  |

**Table S5. (Continued)**

| Methods |  | dose level | | | | Duration | Stop (%) | Overdose (%) | Risk of Poor allocation (%) | Sample size |
| --- | --- | --- | --- | --- | --- | --- | --- | --- | --- | --- |
|  |  | 1 | 2 | 3 | 4 |  |  |  |  |  |
| Scenario 12 | p.true | 0.11 | 0.14 | **0.28** | 0.41 |  |  |  |  |  |
| 3+3 | selection (%) | 14.4 | 35.2 | **28.1** | 11.4 | 15.8 | 3.1 | 0 | 67.3 | 12.2 |
|  | pts at MTD | 3.8 | 3.5 | **3.2** | 1.7 |  |  |  |  |  |
| Keyboard | selection (%) | 7.9 | 27.4 | **45.9** | 18.5 | 29.4 | 0.4 | 0 | 27.7 | 22.6 |
|  | pts at MTD | 6.1 | 7.6 | **6.2** | 2.8 |  |  |  |  |  |
| MEM-Keyboard | selection (%) | 6 | 22.5 | **50.8** | 20.4 | 28.8 | 0.3 | 0 | 21.1 | 22.3 |
|  | pts at MTD | 4.6 | 6 | **7.6** | 4 |  |  |  |  |  |
| TITE-Keyboard | selection (%) | 13.7 | 29.4 | **42** | 14.4 | 18.2 | 0.4 | 0 | 42.9 | 21 |
|  | pts at MTD | 6.3 | 6.7 | **5.7** | 2.3 |  |  |  |  |  |
| TITE-Keyboard (E) | selection (%) | 14.6 | 29.6 | **41.2** | 14.1 | 18 | 0.4 | 0 | 44.9 | 20.9 |
|  | pts at MTD | 6.5 | 6.7 | **5.5** | 2.2 |  |  |  |  |  |
| MEM-TITE-Keyboard | selection (%) | 11.3 | 28.5 | **44.4** | 15.6 | 18.7 | 0.3 | 0 | 38 | 21.6 |
|  | pts at MTD | 6 | 6.9 | **6.1** | 2.6 |  |  |  |  |  |
| MEM-TITE-Keyboard (E) | selection (%) | 12.3 | 29 | **44** | 14.3 | 18.4 | 0.3 | 0 | 40.3 | 21.3 |
|  | pts at MTD | 6.2 | 6.9 | **5.9** | 2.3 |  |  |  |  |  |

**Table S5. (Continued)**

| Methods |  | dose level | | | | Duration | Stop (%) | Overdose (%) | Risk of Poor allocation (%) | Sample size |
| --- | --- | --- | --- | --- | --- | --- | --- | --- | --- | --- |
|  |  | 1 | 2 | 3 | 4 |  |  |  |  |  |
| Scenario 13 | p.true | 0.11 | 0.14 | **0.28** | 0.41 |  |  |  |  |  |
| 3+3 | selection (%) | 14.4 | 35.2 | **28.1** | 11.4 | 15.8 | 3.1 | 0 | 67.3 | 12.2 |
|  | pts at MTD | 3.8 | 3.5 | **3.2** | 1.7 |  |  |  |  |  |
| Keyboard | selection (%) | 7.9 | 27.4 | **45.9** | 18.5 | 29.4 | 0.4 | 0 | 27.7 | 22.6 |
|  | pts at MTD | 6.1 | 7.6 | **6.2** | 2.8 |  |  |  |  |  |
| MEM-Keyboard | selection (%) | 5.9 | 22.7 | **51.6** | 19.5 | 28.8 | 0.3 | 0 | 18.1 | 22.2 |
|  | pts at MTD | 4.7 | 6 | **8.1** | 3.5 |  |  |  |  |  |
| TITE-Keyboard | selection (%) | 13.7 | 29.4 | **42** | 14.4 | 18.2 | 0.4 | 0 | 42.9 | 21 |
|  | pts at MTD | 6.3 | 6.7 | **5.7** | 2.3 |  |  |  |  |  |
| TITE-Keyboard (E) | selection (%) | 14.6 | 29.6 | **41.2** | 14.1 | 18 | 0.4 | 0 | 44.9 | 20.9 |
|  | pts at MTD | 6.5 | 6.7 | **5.5** | 2.2 |  |  |  |  |  |
| MEM-TITE-Keyboard | selection (%) | 10.5 | 27.6 | **45.4** | 16 | 18.9 | 0.4 | 0 | 36.3 | 21.7 |
|  | pts at MTD | 5.9 | 6.9 | **6.2** | 2.6 |  |  |  |  |  |
| MEM-TITE-Keyboard (E) | selection (%) | 11.5 | 28.9 | **44.5** | 14.8 | 18.6 | 0.4 | 0 | 38.9 | 21.5 |
|  | pts at MTD | 6 | 7 | **6.1** | 2.4 |  |  |  |  |  |

The MTD is in boldface. 3+3 is the conventional 3+3 design; Keyboard is the Keyboard design; MEM-Keyboard is the proposed design that incorporates the historical data; TITE-Keyboard and TITE-Keyboard (E) are the time-to-event keyboard designs that utilizes the approximated or exact likelihood function respectively; MEM-TITE-Keyboard and MEM-TITE-Keyboard (E) are the proposed designs. π denotes the different prior exchangeability probabilities.

## Table S6. Simulation results with sample size of 24 and cohort size of 3 (π = 0.2)

| Methods |  | dose level | | | | Duration | Stop (%) | Overdose (%) | Risk of Poor allocation (%) | Sample size |
| --- | --- | --- | --- | --- | --- | --- | --- | --- | --- | --- |
|  |  | 1 | 2 | 3 | 4 |  |  |  |  |  |
| Scenario 1 | p.true | **0.28** | 0.41 | 0.52 | 0.63 |  |  |  |  |  |
| 3+3 | selection (%) | **37.2** | 13.4 | 2.2 | 0.2 | 9.2 | 19.4 | 0.4 | 56.2 | 7.3 |
|  | pts at MTD | **4.3** | 2.3 | 0.6 | 0.1 |  |  |  |  |  |
| Keyboard | selection (%) | **65** | 19.6 | 2.4 | 0.2 | 20.4 | 12.9 | 15 | 6.3 | 16.1 |
|  | pts at MTD | **10.6** | 4.4 | 1 | 0.1 |  |  |  |  |  |
| MEM-Keyboard | selection (%) | **70** | 15.8 | 0.7 | 0 | 17.6 | 13.5 | 2.9 | 5.7 | 13.8 |
|  | pts at MTD | **10.9** | 2.7 | 0.1 | 0 |  |  |  |  |  |
| TITE-Keyboard | selection (%) | **66** | 18.8 | 2.1 | 0.1 | 12.5 | 12.9 | 14 | 8.2 | 15.2 |
|  | pts at MTD | **10.3** | 4 | 0.9 | 0.1 |  |  |  |  |  |
| TITE-Keyboard (E) | selection (%) | **66.7** | 18.8 | 1.8 | 0.1 | 12.4 | 12.6 | 13.4 | 8.4 | 15.2 |
|  | pts at MTD | **10.3** | 3.9 | 0.9 | 0.1 |  |  |  |  |  |
| MEM-TITE-Keyboard | selection (%) | **76** | 10.7 | 0.4 | 0 | 8.5 | 13 | 1.1 | 1.7 | 12.7 |
|  | pts at MTD | **11.5** | 1.2 | 0.1 | 0 |  |  |  |  |  |
| MEM-TITE-Keyboard (E) | selection (%) | **74.4** | 12.6 | 0.4 | 0 | 8.6 | 12.6 | 1.2 | 1.7 | 12.9 |
|  | pts at MTD | **11.5** | 1.3 | 0.1 | 0 |  |  |  |  |  |

**Table S6. (Continued)**

| Methods |  | dose level | | | | Duration | Stop (%) | Overdose (%) | Risk of Poor allocation (%) | Sample size |
| --- | --- | --- | --- | --- | --- | --- | --- | --- | --- | --- |
|  |  | 1 | 2 | 3 | 4 |  |  |  |  |  |
| Scenario 2 | p.true | 0.14 | **0.28** | 0.41 | 0.52 |  |  |  |  |  |
| 3+3 | selection (%) | 38.9 | **31.5** | 11 | 1.7 | 12.7 | 5.5 | 0 | 64.1 | 9.9 |
|  | pts at MTD | 3.9 | **3.6** | 1.9 | 0.5 |  |  |  |  |  |
| Keyboard | selection (%) | 28 | **51.1** | 16.6 | 3.3 | 26.7 | 0.9 | 5.7 | 20.6 | 20.7 |
|  | pts at MTD | 8.8 | **7.8** | 3.4 | 0.7 |  |  |  |  |  |
| MEM-Keyboard | selection (%) | 24.7 | **58.6** | 15 | 0.8 | 26.3 | 1 | 2.3 | 12.5 | 20.4 |
|  | pts at MTD | 8.3 | **9.3** | 2.6 | 0.1 |  |  |  |  |  |
| TITE-Keyboard | selection (%) | 33.7 | **49** | 14.5 | 2 | 15.1 | 0.9 | 6.7 | 35.8 | 19 |
|  | pts at MTD | 8.5 | **7.1** | 2.8 | 0.6 |  |  |  |  |  |
| TITE-Keyboard (E) | selection (%) | 33.6 | **48.8** | 14.8 | 1.9 | 15.1 | 0.9 | 6.6 | 36.4 | 19 |
|  | pts at MTD | 8.6 | **7** | 2.8 | 0.6 |  |  |  |  |  |
| MEM-TITE-Keyboard | selection (%) | 33.2 | **56.5** | 9 | 0.4 | 12.6 | 1 | 0 | 30 | 18.2 |
|  | pts at MTD | 9.4 | **7.7** | 1 | 0 |  |  |  |  |  |
| MEM-TITE-Keyboard (E) | selection (%) | 33.8 | **54.9** | 9.8 | 0.4 | 12.8 | 1.1 | 0 | 32.8 | 18.3 |
|  | pts at MTD | 9.7 | **7.4** | 1.1 | 0 |  |  |  |  |  |

**Table S6. (Continued)**

| Methods |  | dose level | | | | Duration | Stop (%) | Overdose (%) | Risk of Poor allocation (%) | Sample size |
| --- | --- | --- | --- | --- | --- | --- | --- | --- | --- | --- |
|  |  | 1 | 2 | 3 | 4 |  |  |  |  |  |
| Scenario 3 | p.true | 0.11 | 0.14 | **0.28** | 0.41 |  |  |  |  |  |
| 3+3 | selection (%) | 14.4 | 35.2 | **28.1** | 11.4 | 15.8 | 3.2 | 0 | 67.3 | 12.2 |
|  | pts at MTD | 3.8 | 3.5 | **3.2** | 1.7 |  |  |  |  |  |
| Keyboard | selection (%) | 7.9 | 27.4 | **45.9** | 18.5 | 29.4 | 0.4 | 0 | 27.7 | 22.6 |
|  | pts at MTD | 6.1 | 7.6 | **6.2** | 2.8 |  |  |  |  |  |
| MEM-Keyboard | selection (%) | 2.9 | 23.9 | **57.1** | 15.8 | 29.8 | 0.3 | 0 | 13 | 23 |
|  | pts at MTD | 3.4 | 8.1 | **8.9** | 2.5 |  |  |  |  |  |
| TITE-Keyboard | selection (%) | 13.7 | 29.4 | **42** | 14.4 | 18.2 | 0.4 | 0 | 42.9 | 21 |
|  | pts at MTD | 6.3 | 6.7 | **5.7** | 2.3 |  |  |  |  |  |
| TITE-Keyboard (E) | selection (%) | 14.6 | 29.6 | **41.2** | 14.1 | 18 | 0.4 | 0 | 44.9 | 20.9 |
|  | pts at MTD | 6.5 | 6.7 | **5.5** | 2.2 |  |  |  |  |  |
| MEM-TITE-Keyboard | selection (%) | 8.5 | 30.5 | **52.2** | 8.5 | 17.3 | 0.4 | 0 | 34.1 | 21.7 |
|  | pts at MTD | 5.8 | 8.4 | **6.8** | 0.8 |  |  |  |  |  |
| MEM-TITE-Keyboard (E) | selection (%) | 9 | 30.7 | **50** | 10 | 17.4 | 0.3 | 0 | 36.8 | 21.7 |
|  | pts at MTD | 5.7 | 8.6 | **6.5** | 0.9 |  |  |  |  |  |

**Table S6. (Continued)**

| Methods |  | dose level | | | | Duration | Stop (%) | Overdose (%) | Risk of Poor allocation (%) | Sample size |
| --- | --- | --- | --- | --- | --- | --- | --- | --- | --- | --- |
|  |  | 1 | 2 | 3 | 4 |  |  |  |  |  |
| Scenario 4 | p.true | 0.03 | 0.11 | 0.14 | **0.28** |  |  |  |  |  |
| 3+3 | selection (%) | 10.7 | 14.9 | 33.9 | **39.5** | 17.9 | 0.4 | 0 | 68.9 | 13.6 |
|  | pts at MTD | 3.2 | 3.8 | 3.5 | **3.1** |  |  |  |  |  |
| Keyboard | selection (%) | 0.7 | 8.6 | 30.2 | **60.5** | 30.7 | 0 | 0 | 34.4 | 23.4 |
|  | pts at MTD | 4.7 | 5.9 | 6.4 | **6.4** |  |  |  |  |  |
| MEM-Keyboard | selection (%) | 0.4 | 3.6 | 29.6 | **66.4** | 30.1 | 0 | 0 | 10.5 | 23.1 |
|  | pts at MTD | 3 | 3.5 | 7.3 | **9.2** |  |  |  |  |  |
| TITE-Keyboard | selection (%) | 1.6 | 15.3 | 30.8 | **52.3** | 20.8 | 0 | 0 | 46.9 | 22.5 |
|  | pts at MTD | 4.7 | 6.1 | 6.1 | **5.5** |  |  |  |  |  |
| TITE-Keyboard (E) | selection (%) | 1.8 | 15.7 | 31.1 | **51.3** | 20.7 | 0 | 0 | 49 | 22.5 |
|  | pts at MTD | 4.8 | 6.2 | 6.1 | **5.4** |  |  |  |  |  |
| MEM-TITE-Keyboard | selection (%) | 0.8 | 9.4 | 32.2 | **57.6** | 20.9 | 0 | 0 | 37.3 | 23.3 |
|  | pts at MTD | 4.4 | 5.6 | 7.3 | **6** |  |  |  |  |  |
| MEM-TITE-Keyboard (E) | selection (%) | 0.8 | 9.1 | 32.7 | **57.3** | 20.9 | 0 | 0 | 39.3 | 23.3 |
|  | pts at MTD | 4.4 | 5.6 | 7.6 | **5.8** |  |  |  |  |  |

**Table S6. (Continued)**

| Methods |  | dose level | | | | Duration | Stop (%) | Overdose (%) | Risk of Poor allocation (%) | Sample size |
| --- | --- | --- | --- | --- | --- | --- | --- | --- | --- | --- |
|  |  | 1 | 2 | 3 | 4 |  |  |  |  |  |
| Scenario 5 | p.true | 0.14 | **0.28** | 0.41 | 0.52 |  |  |  |  |  |
| 3+3 | selection (%) | 38.9 | **31.5** | 11 | 1.7 | 12.7 | 5.5 | 0 | 64.1 | 9.9 |
|  | pts at MTD | 3.9 | **3.6** | 1.9 | 0.5 |  |  |  |  |  |
| Keyboard | selection (%) | 28 | **51.1** | 16.6 | 3.3 | 26.7 | 0.9 | 5.7 | 20.6 | 20.7 |
|  | pts at MTD | 8.8 | **7.8** | 3.4 | 0.7 |  |  |  |  |  |
| MEM-Keyboard | selection (%) | 23.5 | **59.8** | 15.1 | 0.8 | 24 | 0.7 | 2.2 | 8.4 | 18.7 |
|  | pts at MTD | 5.4 | **10.6** | 2.6 | 0.1 |  |  |  |  |  |
| TITE-Keyboard | selection (%) | 33.7 | **49** | 14.5 | 2 | 15.1 | 0.9 | 6.7 | 35.8 | 19 |
|  | pts at MTD | 8.5 | **7.1** | 2.8 | 0.6 |  |  |  |  |  |
| TITE-Keyboard (E) | selection (%) | 33.6 | **48.8** | 14.8 | 1.9 | 15.1 | 0.9 | 6.6 | 36.4 | 19 |
|  | pts at MTD | 8.6 | **7** | 2.8 | 0.6 |  |  |  |  |  |
| MEM-TITE-Keyboard | selection (%) | 33.4 | **56.4** | 9 | 0.4 | 12.7 | 0.8 | 0 | 29.5 | 18.2 |
|  | pts at MTD | 9.4 | **7.8** | 1 | 0 |  |  |  |  |  |
| MEM-TITE-Keyboard (E) | selection (%) | 33.5 | **55.1** | 10 | 0.5 | 12.8 | 1 | 0 | 32.6 | 18.3 |
|  | pts at MTD | 9.7 | **7.5** | 1.1 | 0 |  |  |  |  |  |

**Table S6. (Continued)**

| Methods |  | dose level | | | | Duration | Stop (%) | Overdose (%) | Risk of Poor allocation (%) | Sample size |
| --- | --- | --- | --- | --- | --- | --- | --- | --- | --- | --- |
|  |  | 1 | 2 | 3 | 4 |  |  |  |  |  |
| Scenario 6 | p.true | 0.14 | **0.28** | 0.41 | 0.52 |  |  |  |  |  |
| 3+3 | selection (%) | 38.9 | **31.5** | 11 | 1.7 | 12.7 | 5.5 | 0 | 64.1 | 9.9 |
|  | pts at MTD | 3.9 | **3.6** | 1.9 | 0.5 |  |  |  |  |  |
| Keyboard | selection (%) | 28 | **51.1** | 16.6 | 3.3 | 26.7 | 0.9 | 5.7 | 20.6 | 20.7 |
|  | pts at MTD | 8.8 | **7.8** | 3.4 | 0.7 |  |  |  |  |  |
| MEM-Keyboard | selection (%) | 24.7 | **58.6** | 15 | 0.8 | 26.3 | 1 | 2.3 | 12.5 | 20.4 |
|  | pts at MTD | 8.3 | **9.3** | 2.6 | 0.1 |  |  |  |  |  |
| TITE-Keyboard | selection (%) | 33.7 | **49** | 14.5 | 2 | 15.1 | 0.9 | 6.7 | 35.8 | 19 |
|  | pts at MTD | 8.5 | **7.1** | 2.8 | 0.6 |  |  |  |  |  |
| TITE-Keyboard (E) | selection (%) | 33.6 | **48.8** | 14.8 | 1.9 | 15.1 | 0.9 | 6.6 | 36.4 | 19 |
|  | pts at MTD | 8.6 | **7** | 2.8 | 0.6 |  |  |  |  |  |
| MEM-TITE-Keyboard | selection (%) | 33.1 | **56.8** | 8.8 | 0.4 | 12.7 | 0.9 | 0 | 32.4 | 18.2 |
|  | pts at MTD | 9.7 | **7.5** | 0.9 | 0 |  |  |  |  |  |
| MEM-TITE-Keyboard (E) | selection (%) | 34.1 | **54.8** | 9.8 | 0.5 | 12.8 | 0.8 | 0 | 33.9 | 18.3 |
|  | pts at MTD | 9.8 | **7.3** | 1.1 | 0 |  |  |  |  |  |

**Table S6. (Continued)**

| Methods |  | dose level | | | | Duration | Stop (%) | Overdose (%) | Risk of Poor allocation (%) | Sample size |
| --- | --- | --- | --- | --- | --- | --- | --- | --- | --- | --- |
|  |  | 1 | 2 | 3 | 4 |  |  |  |  |  |
| Scenario 7 | p.true | 0.14 | **0.28** | 0.41 | 0.52 |  |  |  |  |  |
| 3+3 | selection (%) | 38.9 | **31.5** | 11 | 1.7 | 12.7 | 5.5 | 0 | 64.1 | 9.9 |
|  | pts at MTD | 3.9 | **3.6** | 1.9 | 0.5 |  |  |  |  |  |
| Keyboard | selection (%) | 28 | **51.1** | 16.6 | 3.3 | 26.7 | 0.9 | 5.7 | 20.6 | 20.7 |
|  | pts at MTD | 8.8 | **7.8** | 3.4 | 0.7 |  |  |  |  |  |
| MEM-Keyboard | selection (%) | 24.7 | **58.6** | 15 | 0.8 | 26.3 | 1 | 2.3 | 12.5 | 20.4 |
|  | pts at MTD | 8.3 | **9.3** | 2.6 | 0.1 |  |  |  |  |  |
| TITE-Keyboard | selection (%) | 33.7 | **49** | 14.5 | 2 | 15.1 | 0.9 | 6.7 | 35.8 | 19 |
|  | pts at MTD | 8.5 | **7.1** | 2.8 | 0.6 |  |  |  |  |  |
| TITE-Keyboard (E) | selection (%) | 33.6 | **48.8** | 14.8 | 1.9 | 15.1 | 0.9 | 6.6 | 36.4 | 19 |
|  | pts at MTD | 8.6 | **7** | 2.8 | 0.6 |  |  |  |  |  |
| MEM-TITE-Keyboard | selection (%) | 34.6 | **55.1** | 9 | 0.4 | 12.6 | 0.8 | 0 | 33.1 | 18.1 |
|  | pts at MTD | 9.7 | **7.4** | 1 | 0 |  |  |  |  |  |
| MEM-TITE-Keyboard (E) | selection (%) | 35.7 | **53** | 10.1 | 0.4 | 12.4 | 0.9 | 0.1 | 32.8 | 18 |
|  | pts at MTD | 9.5 | **7.4** | 1.1 | 0 |  |  |  |  |  |

**Table S6. (Continued)**

| Methods |  | dose level | | | | Duration | Stop (%) | Overdose (%) | Risk of Poor allocation (%) | Sample size |
| --- | --- | --- | --- | --- | --- | --- | --- | --- | --- | --- |
|  |  | 1 | 2 | 3 | 4 |  |  |  |  |  |
| Scenario 8 | p.true | 0.11 | 0.14 | **0.28** | 0.41 |  |  |  |  |  |
| 3+3 | selection (%) | 14.4 | 35.2 | **28.1** | 11.4 | 15.8 | 3.1 | 0 | 67.3 | 12.2 |
|  | pts at MTD | 3.8 | 3.5 | **3.2** | 1.7 |  |  |  |  |  |
| Keyboard | selection (%) | 7.9 | 27.4 | **45.9** | 18.5 | 29.4 | 0.4 | 0 | 27.7 | 22.6 |
|  | pts at MTD | 6.1 | 7.6 | **6.2** | 2.8 |  |  |  |  |  |
| MEM-Keyboard | selection (%) | 4.9 | 36.5 | **49.2** | 8.9 | 28 | 0.4 | 0 | 39.5 | 21.5 |
|  | pts at MTD | 5.3 | 9 | **6.4** | 0.9 |  |  |  |  |  |
| TITE-Keyboard | selection (%) | 13.7 | 29.4 | **42** | 14.4 | 18.2 | 0.4 | 0 | 42.9 | 21 |
|  | pts at MTD | 6.3 | 6.7 | **5.7** | 2.3 |  |  |  |  |  |
| TITE-Keyboard (E) | selection (%) | 14.6 | 29.6 | **41.2** | 14.1 | 18 | 0.4 | 0 | 44.9 | 20.9 |
|  | pts at MTD | 6.5 | 6.7 | **5.5** | 2.2 |  |  |  |  |  |
| MEM-TITE-Keyboard | selection (%) | 13.3 | 49.1 | **32.8** | 4.4 | 13.8 | 0.4 | 0 | 66.7 | 19.5 |
|  | pts at MTD | 7.6 | 9.1 | **2.5** | 0.3 |  |  |  |  |  |
| MEM-TITE-Keyboard (E) | selection (%) | 14.7 | 45.1 | **35.4** | 4.5 | 13.9 | 0.4 | 0 | 65.6 | 19.6 |
|  | pts at MTD | 8 | 8.8 | **2.6** | 0.3 |  |  |  |  |  |

**Table S6. (Continued)**

| Methods |  | dose level | | | | Duration | Stop (%) | Overdose (%) | Risk of Poor allocation (%) | Sample size |
| --- | --- | --- | --- | --- | --- | --- | --- | --- | --- | --- |
|  |  | 1 | 2 | 3 | 4 |  |  |  |  |  |
| Scenario 9 | p.true | 0.11 | 0.14 | **0.28** | 0.41 |  |  |  |  |  |
| 3+3 | selection (%) | 14.4 | 35.2 | **28.1** | 11.4 | 15.8 | 3.1 | 0 | 67.3 | 12.2 |
|  | pts at MTD | 3.8 | 3.5 | **3.2** | 1.7 |  |  |  |  |  |
| Keyboard | selection (%) | 7.9 | 27.4 | **45.9** | 18.5 | 29.4 | 0.4 | 0 | 27.7 | 22.6 |
|  | pts at MTD | 6.1 | 7.6 | **6.2** | 2.8 |  |  |  |  |  |
| MEM-Keyboard | selection (%) | 4.7 | 20 | **55.2** | 19.9 | 29.7 | 0.2 | 0 | 11.6 | 23 |
|  | pts at MTD | 3.3 | 5 | **8.9** | 5.9 |  |  |  |  |  |
| TITE-Keyboard | selection (%) | 13.7 | 29.4 | **42** | 14.4 | 18.2 | 0.4 | 0 | 42.9 | 21 |
|  | pts at MTD | 6.3 | 6.7 | **5.7** | 2.3 |  |  |  |  |  |
| TITE-Keyboard (E) | selection (%) | 14.6 | 29.6 | **41.2** | 14.1 | 18 | 0.4 | 0 | 44.9 | 20.9 |
|  | pts at MTD | 6.5 | 6.7 | **5.5** | 2.2 |  |  |  |  |  |
| MEM-TITE-Keyboard | selection (%) | 7.3 | 26.8 | **47.3** | 18.2 | 19.5 | 0.4 | 0 | 24.4 | 22.5 |
|  | pts at MTD | 5.7 | 7.1 | **7.1** | 2.8 |  |  |  |  |  |
| MEM-TITE-Keyboard (E) | selection (%) | 8.2 | 27 | **47.6** | 16.8 | 19.2 | 0.4 | 0 | 25.4 | 22.3 |
|  | pts at MTD | 5.7 | 7 | **7** | 2.6 |  |  |  |  |  |

**Table S6. (Continued)**

| Methods |  | dose level | | | | Duration | Stop (%) | Overdose (%) | Risk of Poor allocation (%) | Sample size |
| --- | --- | --- | --- | --- | --- | --- | --- | --- | --- | --- |
|  |  | 1 | 2 | 3 | 4 |  |  |  |  |  |
| Scenario 10 | p.true | 0.11 | 0.14 | **0.28** | 0.41 |  |  |  |  |  |
| 3+3 | selection (%) | 14.4 | 35.2 | **28.1** | 11.4 | 15.8 | 3.1 | 0 | 67.3 | 12.2 |
|  | pts at MTD | 3.8 | 3.5 | **3.2** | 1.7 |  |  |  |  |  |
| Keyboard | selection (%) | 7.9 | 27.4 | **45.9** | 18.5 | 29.4 | 0.4 | 0 | 27.7 | 22.6 |
|  | pts at MTD | 6.1 | 7.6 | **6.2** | 2.8 |  |  |  |  |  |
| MEM-Keyboard | selection (%) | 3.7 | 36.6 | **50.1** | 9.4 | 26.5 | 0.2 | 0 | 38.9 | 20.4 |
|  | pts at MTD | 3.6 | 9.3 | **6.5** | 1 |  |  |  |  |  |
| TITE-Keyboard | selection (%) | 13.7 | 29.4 | **42** | 14.4 | 18.2 | 0.4 | 0 | 42.9 | 21 |
|  | pts at MTD | 6.3 | 6.7 | **5.7** | 2.3 |  |  |  |  |  |
| TITE-Keyboard (E) | selection (%) | 14.6 | 29.6 | **41.2** | 14.1 | 18 | 0.4 | 0 | 44.9 | 20.9 |
|  | pts at MTD | 6.5 | 6.7 | **5.5** | 2.2 |  |  |  |  |  |
| MEM-TITE-Keyboard | selection (%) | 13.4 | 49.4 | **32.6** | 4.2 | 13.7 | 0.4 | 0 | 67.2 | 19.5 |
|  | pts at MTD | 7.6 | 9.1 | **2.5** | 0.2 |  |  |  |  |  |
| MEM-TITE-Keyboard (E) | selection (%) | 14.2 | 45.7 | **35.1** | 4.6 | 13.9 | 0.4 | 0 | 65.8 | 19.7 |
|  | pts at MTD | 8 | 8.8 | **2.6** | 0.3 |  |  |  |  |  |

**Table S6. (Continued)**

| Methods |  | dose level | | | | Duration | Stop (%) | Overdose (%) | Risk of Poor allocation (%) | Sample size |
| --- | --- | --- | --- | --- | --- | --- | --- | --- | --- | --- |
|  |  | 1 | 2 | 3 | 4 |  |  |  |  |  |
| Scenario 11 | p.true | 0.11 | 0.14 | **0.28** | 0.41 |  |  |  |  |  |
| 3+3 | selection (%) | 14.4 | 35.2 | **28.1** | 11.4 | 15.8 | 3.1 | 0 | 67.3 | 12.2 |
|  | pts at MTD | 3.8 | 3.5 | **3.2** | 1.7 |  |  |  |  |  |
| Keyboard | selection (%) | 7.9 | 27.4 | **45.9** | 18.5 | 29.4 | 0.4 | 0 | 27.7 | 22.6 |
|  | pts at MTD | 6.1 | 7.6 | **6.2** | 2.8 |  |  |  |  |  |
| MEM-Keyboard | selection (%) | 4.9 | 36.5 | **49.2** | 8.9 | 28 | 0.4 | 0 | 39.5 | 21.5 |
|  | pts at MTD | 5.3 | 9 | **6.4** | 0.9 |  |  |  |  |  |
| TITE-Keyboard | selection (%) | 13.7 | 29.4 | **42** | 14.4 | 18.2 | 0.4 | 0 | 42.9 | 21 |
|  | pts at MTD | 6.3 | 6.7 | **5.7** | 2.3 |  |  |  |  |  |
| TITE-Keyboard (E) | selection (%) | 14.6 | 29.6 | **41.2** | 14.1 | 18 | 0.4 | 0 | 44.9 | 20.9 |
|  | pts at MTD | 6.5 | 6.7 | **5.5** | 2.2 |  |  |  |  |  |
| MEM-TITE-Keyboard | selection (%) | 14.3 | 49.2 | **31.5** | 4.5 | 13.7 | 0.5 | 0 | 68.3 | 19.5 |
|  | pts at MTD | 8.1 | 8.7 | **2.4** | 0.2 |  |  |  |  |  |
| MEM-TITE-Keyboard (E) | selection (%) | 15.2 | 45.1 | **34.7** | 4.7 | 14 | 0.4 | 0 | 65.2 | 19.7 |
|  | pts at MTD | 8.2 | 8.6 | **2.7** | 0.3 |  |  |  |  |  |

**Table S6. (Continued)**

| Methods |  | dose level | | | | Duration | Stop (%) | Overdose (%) | Risk of Poor allocation (%) | Sample size |
| --- | --- | --- | --- | --- | --- | --- | --- | --- | --- | --- |
|  |  | 1 | 2 | 3 | 4 |  |  |  |  |  |
| Scenario 12 | p.true | 0.11 | 0.14 | **0.28** | 0.41 |  |  |  |  |  |
| 3+3 | selection (%) | 14.4 | 35.2 | **28.1** | 11.4 | 15.8 | 3.1 | 0 | 67.3 | 12.2 |
|  | pts at MTD | 3.8 | 3.5 | **3.2** | 1.7 |  |  |  |  |  |
| Keyboard | selection (%) | 7.9 | 27.4 | **45.9** | 18.5 | 29.4 | 0.4 | 0 | 27.7 | 22.6 |
|  | pts at MTD | 6.1 | 7.6 | **6.2** | 2.8 |  |  |  |  |  |
| MEM-Keyboard | selection (%) | 4.1 | 20.1 | **50.9** | 24.7 | 29.1 | 0.3 | 0 | 32.4 | 22.6 |
|  | pts at MTD | 3.2 | 4.8 | **7.2** | 7.4 |  |  |  |  |  |
| TITE-Keyboard | selection (%) | 13.7 | 29.4 | **42** | 14.4 | 18.2 | 0.4 | 0 | 42.9 | 21 |
|  | pts at MTD | 6.3 | 6.7 | **5.7** | 2.3 |  |  |  |  |  |
| TITE-Keyboard (E) | selection (%) | 14.6 | 29.6 | **41.2** | 14.1 | 18 | 0.4 | 0 | 44.9 | 20.9 |
|  | pts at MTD | 6.5 | 6.7 | **5.5** | 2.2 |  |  |  |  |  |
| MEM-TITE-Keyboard | selection (%) | 7.2 | 26.9 | **47.2** | 18.3 | 19.5 | 0.4 | 0 | 24.3 | 22.6 |
|  | pts at MTD | 5.7 | 7 | **7** | 2.8 |  |  |  |  |  |
| MEM-TITE-Keyboard (E) | selection (%) | 8.3 | 26.8 | **47.7** | 16.9 | 19.2 | 0.4 | 0 | 25.5 | 22.4 |
|  | pts at MTD | 5.7 | 7 | **7** | 2.6 |  |  |  |  |  |

**Table S6. (Continued)**

| Methods |  | dose level | | | | Duration | Stop (%) | Overdose (%) | Risk of Poor allocation (%) | Sample size |
| --- | --- | --- | --- | --- | --- | --- | --- | --- | --- | --- |
|  |  | 1 | 2 | 3 | 4 |  |  |  |  |  |
| Scenario 13 | p.true | 0.11 | 0.14 | **0.28** | 0.41 |  |  |  |  |  |
| 3+3 | selection (%) | 14.4 | 35.2 | **28.1** | 11.4 | 15.8 | 3.1 | 0 | 67.3 | 12.2 |
|  | pts at MTD | 3.8 | 3.5 | **3.2** | 1.7 |  |  |  |  |  |
| Keyboard | selection (%) | 7.9 | 27.4 | **45.9** | 18.5 | 29.4 | 0.4 | 0 | 27.7 | 22.6 |
|  | pts at MTD | 6.1 | 7.6 | **6.2** | 2.8 |  |  |  |  |  |
| MEM-Keyboard | selection (%) | 4.3 | 19.8 | **55.9** | 19.7 | 29.8 | 0.3 | 0 | 11 | 23.1 |
|  | pts at MTD | 3.2 | 5 | **9** | 5.9 |  |  |  |  |  |
| TITE-Keyboard | selection (%) | 13.7 | 29.4 | **42** | 14.4 | 18.2 | 0.4 | 0 | 42.9 | 21 |
|  | pts at MTD | 6.3 | 6.7 | **5.7** | 2.3 |  |  |  |  |  |
| TITE-Keyboard (E) | selection (%) | 14.6 | 29.6 | **41.2** | 14.1 | 18 | 0.4 | 0 | 44.9 | 20.9 |
|  | pts at MTD | 6.5 | 6.7 | **5.5** | 2.2 |  |  |  |  |  |
| MEM-TITE-Keyboard | selection (%) | 5.6 | 27.1 | **49** | 17.9 | 19.7 | 0.4 | 0 | 21.6 | 22.8 |
|  | pts at MTD | 5.7 | 7.2 | **7.3** | 2.6 |  |  |  |  |  |
| MEM-TITE-Keyboard (E) | selection (%) | 6.3 | 27.1 | **48.8** | 17.4 | 19.6 | 0.4 | 0 | 22.4 | 22.7 |
|  | pts at MTD | 5.6 | 7.2 | **7.3** | 2.5 |  |  |  |  |  |

The MTD is in boldface. 3+3 is the conventional 3+3 design; Keyboard is the Keyboard design; MEM-Keyboard is the proposed design that incorporates the historical data; TITE-Keyboard and TITE-Keyboard (E) are the time-to-event keyboard designs that utilizes the approximated or exact likelihood function respectively; MEM-TITE-Keyboard and MEM-TITE-Keyboard (E) are the proposed designs. π denotes the different prior exchangeability probabilities.

## Table S7. Simulation results with sample size of 24 and cohort size of 3 (π = 0. 5)

| Methods |  | dose level | | | | Duration | Stop (%) | Overdose (%) | Risk of Poor allocation (%) | Sample size |
| --- | --- | --- | --- | --- | --- | --- | --- | --- | --- | --- |
|  |  | 1 | 2 | 3 | 4 |  |  |  |  |  |
| Scenario 1 | p.true | **0.28** | 0.41 | 0.52 | 0.63 |  |  |  |  |  |
| 3+3 | selection (%) | **37.2** | 13.4 | 2.2 | 0.2 | 9.2 | 19.4 | 0.4 | 56.2 | 7.3 |
|  | pts at MTD | **4.3** | 2.3 | 0.6 | 0.1 |  |  |  |  |  |
| Keyboard | selection (%) | **65** | 19.6 | 2.4 | 0.2 | 20.4 | 12.9 | 15 | 6.3 | 16.1 |
|  | pts at MTD | **10.6** | 4.4 | 1 | 0.1 |  |  |  |  |  |
| MEM-Keyboard | selection (%) | **79.3** | 7.2 | 0 | 0 | 15.4 | 13.4 | 0 | 2 | 12 |
|  | pts at MTD | **11.4** | 0.6 | 0 | 0 |  |  |  |  |  |
| TITE-Keyboard | selection (%) | **66** | 18.8 | 2.1 | 0.1 | 12.5 | 12.9 | 14 | 8.2 | 15.2 |
|  | pts at MTD | **10.3** | 4 | 0.9 | 0.1 |  |  |  |  |  |
| TITE-Keyboard (E) | selection (%) | **66.7** | 18.8 | 1.8 | 0.1 | 12.4 | 12.6 | 13.4 | 8.4 | 15.2 |
|  | pts at MTD | **10.3** | 3.9 | 0.9 | 0.1 |  |  |  |  |  |
| MEM-TITE-Keyboard | selection (%) | **83.7** | 3.8 | 0 | 0 | 7.6 | 12.5 | 0 | 1.8 | 11.7 |
|  | pts at MTD | **11.5** | 0.2 | 0 | 0 |  |  |  |  |  |
| MEM-TITE-Keyboard (E) | selection (%) | **82.4** | 5.1 | 0 | 0 | 7.6 | 12.6 | 0 | 1.7 | 11.8 |
|  | pts at MTD | **11.5** | 0.3 | 0 | 0 |  |  |  |  |  |

**Table S7. (Continued)**

| Methods |  | dose level | | | | Duration | Stop (%) | Overdose (%) | Risk of Poor allocation (%) | Sample size |
| --- | --- | --- | --- | --- | --- | --- | --- | --- | --- | --- |
|  |  | 1 | 2 | 3 | 4 |  |  |  |  |  |
| Scenario 2 | p.true | 0.14 | **0.28** | 0.41 | 0.52 |  |  |  |  |  |
| 3+3 | selection (%) | 38.9 | **31.5** | 11 | 1.7 | 12.7 | 5.5 | 0 | 64.1 | 9.9 |
|  | pts at MTD | 3.9 | **3.6** | 1.9 | 0.5 |  |  |  |  |  |
| Keyboard | selection (%) | 28 | **51.1** | 16.6 | 3.3 | 26.7 | 0.9 | 5.7 | 20.6 | 20.7 |
|  | pts at MTD | 8.8 | **7.8** | 3.4 | 0.7 |  |  |  |  |  |
| MEM-Keyboard | selection (%) | 23.6 | **68.2** | 7.4 | 0 | 24.6 | 0.8 | 0 | 4.2 | 19 |
|  | pts at MTD | 8.1 | **10.3** | 0.6 | 0 |  |  |  |  |  |
| TITE-Keyboard | selection (%) | 33.7 | **49** | 14.5 | 2 | 15.1 | 0.9 | 6.7 | 35.8 | 19 |
|  | pts at MTD | 8.5 | **7.1** | 2.8 | 0.6 |  |  |  |  |  |
| TITE-Keyboard (E) | selection (%) | 33.6 | **48.8** | 14.8 | 1.9 | 15.1 | 0.9 | 6.6 | 36.4 | 19 |
|  | pts at MTD | 8.6 | **7** | 2.8 | 0.6 |  |  |  |  |  |
| MEM-TITE-Keyboard | selection (%) | 32.9 | **63.8** | 2.4 | 0 | 12 | 0.9 | 0 | 35.7 | 17.8 |
|  | pts at MTD | 10.7 | **6.9** | 0.1 | 0 |  |  |  |  |  |
| MEM-TITE-Keyboard (E) | selection (%) | 34.5 | **61.2** | 3.3 | 0 | 12 | 1 | 0 | 38.5 | 17.7 |
|  | pts at MTD | 10.8 | **6.7** | 0.2 | 0 |  |  |  |  |  |

**Table S7. (Continued)**

| Methods |  | dose level | | | | Duration | Stop (%) | Overdose (%) | Risk of Poor allocation (%) | Sample size |
| --- | --- | --- | --- | --- | --- | --- | --- | --- | --- | --- |
|  |  | 1 | 2 | 3 | 4 |  |  |  |  |  |
| Scenario 3 | p.true | 0.11 | 0.14 | **0.28** | 0.41 |  |  |  |  |  |
| 3+3 | selection (%) | 14.4 | 35.2 | **28.1** | 11.4 | 15.8 | 3.2 | 0 | 67.3 | 12.2 |
|  | pts at MTD | 3.8 | 3.5 | **3.2** | 1.7 |  |  |  |  |  |
| Keyboard | selection (%) | 7.9 | 27.4 | **45.9** | 18.5 | 29.4 | 0.4 | 0 | 27.7 | 22.6 |
|  | pts at MTD | 6.1 | 7.6 | **6.2** | 2.8 |  |  |  |  |  |
| MEM-Keyboard | selection (%) | 3.2 | 22.7 | **66.2** | 7.7 | 28.6 | 0.1 | 0 | 4.3 | 22.1 |
|  | pts at MTD | 3 | 8.2 | **10.3** | 0.6 |  |  |  |  |  |
| TITE-Keyboard | selection (%) | 13.7 | 29.4 | **42** | 14.4 | 18.2 | 0.4 | 0 | 42.9 | 21 |
|  | pts at MTD | 6.3 | 6.7 | **5.7** | 2.3 |  |  |  |  |  |
| TITE-Keyboard (E) | selection (%) | 14.6 | 29.6 | **41.2** | 14.1 | 18 | 0.4 | 0 | 44.9 | 20.9 |
|  | pts at MTD | 6.5 | 6.7 | **5.5** | 2.2 |  |  |  |  |  |
| MEM-TITE-Keyboard | selection (%) | 4.8 | 31.4 | **61** | 2.5 | 17.6 | 0.4 | 0 | 37.4 | 22.1 |
|  | pts at MTD | 5.6 | 9.9 | **6.5** | 0.1 |  |  |  |  |  |
| MEM-TITE-Keyboard (E) | selection (%) | 6.1 | 31.5 | **58.8** | 3.3 | 17.3 | 0.3 | 0 | 40.7 | 21.9 |
|  | pts at MTD | 5.6 | 9.9 | **6.2** | 0.2 |  |  |  |  |  |

**Table S7. (Continued)**

| Methods |  | dose level | | | | Duration | Stop (%) | Overdose (%) | Risk of Poor allocation (%) | Sample size |
| --- | --- | --- | --- | --- | --- | --- | --- | --- | --- | --- |
|  |  | 1 | 2 | 3 | 4 |  |  |  |  |  |
| Scenario 4 | p.true | 0.03 | 0.11 | 0.14 | **0.28** |  |  |  |  |  |
| 3+3 | selection (%) | 10.7 | 14.9 | 33.9 | **39.5** | 17.9 | 0.4 | 0 | 68.9 | 13.6 |
|  | pts at MTD | 3.2 | 3.8 | 3.5 | **3.1** |  |  |  |  |  |
| Keyboard | selection (%) | 0.7 | 8.6 | 30.2 | **60.5** | 30.7 | 0 | 0 | 34.4 | 23.4 |
|  | pts at MTD | 4.7 | 5.9 | 6.4 | **6.4** |  |  |  |  |  |
| MEM-Keyboard | selection (%) | 0.5 | 3.5 | 28.6 | **67.5** | 30.1 | 0 | 0 | 4 | 23.1 |
|  | pts at MTD | 3 | 3 | 7.2 | **9.9** |  |  |  |  |  |
| TITE-Keyboard | selection (%) | 1.6 | 15.3 | 30.8 | **52.3** | 20.8 | 0 | 0 | 46.9 | 22.5 |
|  | pts at MTD | 4.7 | 6.1 | 6.1 | **5.5** |  |  |  |  |  |
| TITE-Keyboard (E) | selection (%) | 1.8 | 15.7 | 31.1 | **51.3** | 20.7 | 0 | 0 | 49 | 22.5 |
|  | pts at MTD | 4.8 | 6.2 | 6.1 | **5.4** |  |  |  |  |  |
| MEM-TITE-Keyboard | selection (%) | 0.5 | 6.2 | 34.6 | **58.7** | 21.3 | 0 | 0 | 42.8 | 23.7 |
|  | pts at MTD | 4.4 | 5.5 | 8.4 | **5.4** |  |  |  |  |  |
| MEM-TITE-Keyboard (E) | selection (%) | 0.4 | 6.8 | 35.3 | **57.5** | 21.2 | 0 | 0 | 44.8 | 23.6 |
|  | pts at MTD | 4.4 | 5.5 | 8.5 | **5.2** |  |  |  |  |  |

**Table S7. (Continued)**

| Methods |  | dose level | | | | Duration | Stop (%) | Overdose (%) | Risk of Poor allocation (%) | Sample size |
| --- | --- | --- | --- | --- | --- | --- | --- | --- | --- | --- |
|  |  | 1 | 2 | 3 | 4 |  |  |  |  |  |
| Scenario 5 | p.true | 0.14 | **0.28** | 0.41 | 0.52 |  |  |  |  |  |
| 3+3 | selection (%) | 38.9 | **31.5** | 11 | 1.7 | 12.7 | 5.5 | 0 | 64.1 | 9.9 |
|  | pts at MTD | 3.9 | **3.6** | 1.9 | 0.5 |  |  |  |  |  |
| Keyboard | selection (%) | 28 | **51.1** | 16.6 | 3.3 | 26.7 | 0.9 | 5.7 | 20.6 | 20.7 |
|  | pts at MTD | 8.8 | **7.8** | 3.4 | 0.7 |  |  |  |  |  |
| MEM-Keyboard | selection (%) | 22.9 | **69.5** | 7 | 0 | 22 | 0.7 | 0 | 2.6 | 17.1 |
|  | pts at MTD | 5.2 | **11.3** | 0.6 | 0 |  |  |  |  |  |
| TITE-Keyboard | selection (%) | 33.7 | **49** | 14.5 | 2 | 15.1 | 0.9 | 6.7 | 35.8 | 19 |
|  | pts at MTD | 8.5 | **7.1** | 2.8 | 0.6 |  |  |  |  |  |
| TITE-Keyboard (E) | selection (%) | 33.6 | **48.8** | 14.8 | 1.9 | 15.1 | 0.9 | 6.6 | 36.4 | 19 |
|  | pts at MTD | 8.6 | **7** | 2.8 | 0.6 |  |  |  |  |  |
| MEM-TITE-Keyboard | selection (%) | 32.6 | **64.1** | 2.3 | 0 | 12.1 | 1 | 0 | 34 | 17.9 |
|  | pts at MTD | 10.7 | **7** | 0.1 | 0 |  |  |  |  |  |
| MEM-TITE-Keyboard (E) | selection (%) | 34 | **61.8** | 3.2 | 0 | 12 | 0.9 | 0 | 37.8 | 17.7 |
|  | pts at MTD | 10.8 | **6.8** | 0.2 | 0 |  |  |  |  |  |

**Table S7. (Continued)**

| Methods |  | dose level | | | | Duration | Stop (%) | Overdose (%) | Risk of Poor allocation (%) | Sample size |
| --- | --- | --- | --- | --- | --- | --- | --- | --- | --- | --- |
|  |  | 1 | 2 | 3 | 4 |  |  |  |  |  |
| Scenario 6 | p.true | 0.14 | **0.28** | 0.41 | 0.52 |  |  |  |  |  |
| 3+3 | selection (%) | 38.9 | **31.5** | 11 | 1.7 | 12.7 | 5.5 | 0 | 64.1 | 9.9 |
|  | pts at MTD | 3.9 | **3.6** | 1.9 | 0.5 |  |  |  |  |  |
| Keyboard | selection (%) | 28 | **51.1** | 16.6 | 3.3 | 26.7 | 0.9 | 5.7 | 20.6 | 20.7 |
|  | pts at MTD | 8.8 | **7.8** | 3.4 | 0.7 |  |  |  |  |  |
| MEM-Keyboard | selection (%) | 23.6 | **68.2** | 7.4 | 0 | 24.6 | 0.8 | 0 | 4.2 | 19 |
|  | pts at MTD | 8.1 | **10.3** | 0.6 | 0 |  |  |  |  |  |
| TITE-Keyboard | selection (%) | 33.7 | **49** | 14.5 | 2 | 15.1 | 0.9 | 6.7 | 35.8 | 19 |
|  | pts at MTD | 8.5 | **7.1** | 2.8 | 0.6 |  |  |  |  |  |
| TITE-Keyboard (E) | selection (%) | 33.6 | **48.8** | 14.8 | 1.9 | 15.1 | 0.9 | 6.6 | 36.4 | 19 |
|  | pts at MTD | 8.6 | **7** | 2.8 | 0.6 |  |  |  |  |  |
| MEM-TITE-Keyboard | selection (%) | 34 | **62.5** | 2.4 | 0 | 11.9 | 1 | 0 | 38.7 | 17.6 |
|  | pts at MTD | 10.8 | **6.7** | 0.1 | 0 |  |  |  |  |  |
| MEM-TITE-Keyboard (E) | selection (%) | 35.5 | **60.2** | 3.3 | 0 | 11.7 | 1 | 0 | 40.9 | 17.4 |
|  | pts at MTD | 10.9 | **6.3** | 0.2 | 0 |  |  |  |  |  |

**Table S7. (Continued)**

| Methods |  | dose level | | | | Duration | Stop (%) | Overdose (%) | Risk of Poor allocation (%) | Sample size |
| --- | --- | --- | --- | --- | --- | --- | --- | --- | --- | --- |
|  |  | 1 | 2 | 3 | 4 |  |  |  |  |  |
| Scenario 7 | p.true | 0.14 | **0.28** | 0.41 | 0.52 |  |  |  |  |  |
| 3+3 | selection (%) | 38.9 | **31.5** | 11 | 1.7 | 12.7 | 5.5 | 0 | 64.1 | 9.9 |
|  | pts at MTD | 3.9 | **3.6** | 1.9 | 0.5 |  |  |  |  |  |
| Keyboard | selection (%) | 28 | **51.1** | 16.6 | 3.3 | 26.7 | 0.9 | 5.7 | 20.6 | 20.7 |
|  | pts at MTD | 8.8 | **7.8** | 3.4 | 0.7 |  |  |  |  |  |
| MEM-Keyboard | selection (%) | 23.6 | **68.2** | 7.4 | 0 | 24.6 | 0.8 | 0 | 4.2 | 19 |
|  | pts at MTD | 8.1 | **10.3** | 0.6 | 0 |  |  |  |  |  |
| TITE-Keyboard | selection (%) | 33.7 | **49** | 14.5 | 2 | 15.1 | 0.9 | 6.7 | 35.8 | 19 |
|  | pts at MTD | 8.5 | **7.1** | 2.8 | 0.6 |  |  |  |  |  |
| TITE-Keyboard (E) | selection (%) | 33.6 | **48.8** | 14.8 | 1.9 | 15.1 | 0.9 | 6.6 | 36.4 | 19 |
|  | pts at MTD | 8.6 | **7** | 2.8 | 0.6 |  |  |  |  |  |
| MEM-TITE-Keyboard | selection (%) | 37.1 | **59.8** | 2.2 | 0 | 11.1 | 0.9 | 0 | 44.1 | 17 |
|  | pts at MTD | 11.1 | **5.8** | 0.1 | 0 |  |  |  |  |  |
| MEM-TITE-Keyboard (E) | selection (%) | 40.6 | **55.4** | 3.2 | 0 | 10.2 | 0.8 | 0 | 50.6 | 16.5 |
|  | pts at MTD | 11.4 | **4.9** | 0.2 | 0 |  |  |  |  |  |

**Table S7. (Continued)**

| Methods |  | dose level | | | | Duration | Stop (%) | Overdose (%) | Risk of Poor allocation (%) | Sample size |
| --- | --- | --- | --- | --- | --- | --- | --- | --- | --- | --- |
|  |  | 1 | 2 | 3 | 4 |  |  |  |  |  |
| Scenario 8 | p.true | 0.11 | 0.14 | **0.28** | 0.41 |  |  |  |  |  |
| 3+3 | selection (%) | 14.4 | 35.2 | **28.1** | 11.4 | 15.8 | 3.1 | 0 | 67.3 | 12.2 |
|  | pts at MTD | 3.8 | 3.5 | **3.2** | 1.7 |  |  |  |  |  |
| Keyboard | selection (%) | 7.9 | 27.4 | **45.9** | 18.5 | 29.4 | 0.4 | 0 | 27.7 | 22.6 |
|  | pts at MTD | 6.1 | 7.6 | **6.2** | 2.8 |  |  |  |  |  |
| MEM-Keyboard | selection (%) | 3.8 | 62.3 | **33.6** | 0 | 24.6 | 0.3 | 0 | 75 | 18.8 |
|  | pts at MTD | 5.2 | 11.5 | **2.1** | 0 |  |  |  |  |  |
| TITE-Keyboard | selection (%) | 13.7 | 29.4 | **42** | 14.4 | 18.2 | 0.4 | 0 | 42.9 | 21 |
|  | pts at MTD | 6.3 | 6.7 | **5.7** | 2.3 |  |  |  |  |  |
| TITE-Keyboard (E) | selection (%) | 14.6 | 29.6 | **41.2** | 14.1 | 18 | 0.4 | 0 | 44.9 | 20.9 |
|  | pts at MTD | 6.5 | 6.7 | **5.5** | 2.2 |  |  |  |  |  |
| MEM-TITE-Keyboard | selection (%) | 14.3 | 71.9 | **13.3** | 0 | 12.2 | 0.4 | 0 | 100 | 18.3 |
|  | pts at MTD | 9.2 | 8.5 | **0.5** | 0 |  |  |  |  |  |
| MEM-TITE-Keyboard (E) | selection (%) | 14.9 | 67.5 | **17.3** | 0 | 12.2 | 0.3 | 0 | 100 | 18.4 |
|  | pts at MTD | 9.3 | 8.4 | **0.7** | 0 |  |  |  |  |  |

**Table S7. (Continued)**

| Methods |  | dose level | | | | Duration | Stop (%) | Overdose (%) | Risk of Poor allocation (%) | Sample size |
| --- | --- | --- | --- | --- | --- | --- | --- | --- | --- | --- |
|  |  | 1 | 2 | 3 | 4 |  |  |  |  |  |
| Scenario 9 | p.true | 0.11 | 0.14 | **0.28** | 0.41 |  |  |  |  |  |
| 3+3 | selection (%) | 14.4 | 35.2 | **28.1** | 11.4 | 15.8 | 3.1 | 0 | 67.3 | 12.2 |
|  | pts at MTD | 3.8 | 3.5 | **3.2** | 1.7 |  |  |  |  |  |
| Keyboard | selection (%) | 7.9 | 27.4 | **45.9** | 18.5 | 29.4 | 0.4 | 0 | 27.7 | 22.6 |
|  | pts at MTD | 6.1 | 7.6 | **6.2** | 2.8 |  |  |  |  |  |
| MEM-Keyboard | selection (%) | 5.4 | 17.7 | **56.1** | 20.4 | 29.5 | 0.3 | 0 | 7.8 | 23 |
|  | pts at MTD | 3.1 | 3.4 | **9.4** | 7 |  |  |  |  |  |
| TITE-Keyboard | selection (%) | 13.7 | 29.4 | **42** | 14.4 | 18.2 | 0.4 | 0 | 42.9 | 21 |
|  | pts at MTD | 6.3 | 6.7 | **5.7** | 2.3 |  |  |  |  |  |
| TITE-Keyboard (E) | selection (%) | 14.6 | 29.6 | **41.2** | 14.1 | 18 | 0.4 | 0 | 44.9 | 20.9 |
|  | pts at MTD | 6.5 | 6.7 | **5.5** | 2.2 |  |  |  |  |  |
| MEM-TITE-Keyboard | selection (%) | 5 | 25.3 | **51** | 18.4 | 20.1 | 0.3 | 0 | 15.3 | 23.1 |
|  | pts at MTD | 5.6 | 7.1 | **7.8** | 2.5 |  |  |  |  |  |
| MEM-TITE-Keyboard (E) | selection (%) | 5.2 | 25.9 | **51.6** | 17 | 19.9 | 0.3 | 0 | 16.7 | 22.9 |
|  | pts at MTD | 5.6 | 7.1 | **7.8** | 2.4 |  |  |  |  |  |

**Table S7. (Continued)**

| Methods |  | dose level | | | | Duration | Stop (%) | Overdose (%) | Risk of Poor allocation (%) | Sample size |
| --- | --- | --- | --- | --- | --- | --- | --- | --- | --- | --- |
|  |  | 1 | 2 | 3 | 4 |  |  |  |  |  |
| Scenario 10 | p.true | 0.11 | 0.14 | **0.28** | 0.41 |  |  |  |  |  |
| 3+3 | selection (%) | 14.4 | 35.2 | **28.1** | 11.4 | 15.8 | 3.1 | 0 | 67.3 | 12.2 |
|  | pts at MTD | 3.8 | 3.5 | **3.2** | 1.7 |  |  |  |  |  |
| Keyboard | selection (%) | 7.9 | 27.4 | **45.9** | 18.5 | 29.4 | 0.4 | 0 | 27.7 | 22.6 |
|  | pts at MTD | 6.1 | 7.6 | **6.2** | 2.8 |  |  |  |  |  |
| MEM-Keyboard | selection (%) | 3.6 | 62.5 | **33.5** | 0 | 22.7 | 0.3 | 0 | 74.8 | 17.4 |
|  | pts at MTD | 3.5 | 11.7 | **2.1** | 0 |  |  |  |  |  |
| TITE-Keyboard | selection (%) | 13.7 | 29.4 | **42** | 14.4 | 18.2 | 0.4 | 0 | 42.9 | 21 |
|  | pts at MTD | 6.3 | 6.7 | **5.7** | 2.3 |  |  |  |  |  |
| TITE-Keyboard (E) | selection (%) | 14.6 | 29.6 | **41.2** | 14.1 | 18 | 0.4 | 0 | 44.9 | 20.9 |
|  | pts at MTD | 6.5 | 6.7 | **5.5** | 2.2 |  |  |  |  |  |
| MEM-TITE-Keyboard | selection (%) | 13.4 | 74.1 | **12.1** | 0 | 12.2 | 0.4 | 0 | 100 | 18.3 |
|  | pts at MTD | 9.2 | 8.6 | **0.5** | 0 |  |  |  |  |  |
| MEM-TITE-Keyboard (E) | selection (%) | 14.9 | 68.2 | **16.5** | 0 | 12.2 | 0.4 | 0 | 100 | 18.3 |
|  | pts at MTD | 9.3 | 8.3 | **0.6** | 0 |  |  |  |  |  |

**Table S7. (Continued)**

| Methods |  | dose level | | | | Duration | Stop (%) | Overdose (%) | Risk of Poor allocation (%) | Sample size |
| --- | --- | --- | --- | --- | --- | --- | --- | --- | --- | --- |
|  |  | 1 | 2 | 3 | 4 |  |  |  |  |  |
| Scenario 11 | p.true | 0.11 | 0.14 | **0.28** | 0.41 |  |  |  |  |  |
| 3+3 | selection (%) | 14.4 | 35.2 | **28.1** | 11.4 | 15.8 | 3.1 | 0 | 67.3 | 12.2 |
|  | pts at MTD | 3.8 | 3.5 | **3.2** | 1.7 |  |  |  |  |  |
| Keyboard | selection (%) | 7.9 | 27.4 | **45.9** | 18.5 | 29.4 | 0.4 | 0 | 27.7 | 22.6 |
|  | pts at MTD | 6.1 | 7.6 | **6.2** | 2.8 |  |  |  |  |  |
| MEM-Keyboard | selection (%) | 3.7 | 62.2 | **32.9** | 0.9 | 24.6 | 0.3 | 0 | 75 | 18.8 |
|  | pts at MTD | 5.2 | 11.5 | **2.1** | 0 |  |  |  |  |  |
| TITE-Keyboard | selection (%) | 13.7 | 29.4 | **42** | 14.4 | 18.2 | 0.4 | 0 | 42.9 | 21 |
|  | pts at MTD | 6.3 | 6.7 | **5.7** | 2.3 |  |  |  |  |  |
| TITE-Keyboard (E) | selection (%) | 14.6 | 29.6 | **41.2** | 14.1 | 18 | 0.4 | 0 | 44.9 | 20.9 |
|  | pts at MTD | 6.5 | 6.7 | **5.5** | 2.2 |  |  |  |  |  |
| MEM-TITE-Keyboard | selection (%) | 15 | 72.9 | **11.7** | 0 | 12 | 0.4 | 0 | 100 | 18.1 |
|  | pts at MTD | 9.4 | 8.2 | **0.4** | 0 |  |  |  |  |  |
| MEM-TITE-Keyboard (E) | selection (%) | 16.6 | 66.1 | **16.9** | 0 | 12 | 0.4 | 0 | 100 | 18.2 |
|  | pts at MTD | 9.6 | 8 | **0.6** | 0 |  |  |  |  |  |

**Table S7. (Continued)**

| Methods |  | dose level | | | | Duration | Stop (%) | Overdose (%) | Risk of Poor allocation (%) | Sample size |
| --- | --- | --- | --- | --- | --- | --- | --- | --- | --- | --- |
|  |  | 1 | 2 | 3 | 4 |  |  |  |  |  |
| Scenario 12 | p.true | 0.11 | 0.14 | **0.28** | 0.41 |  |  |  |  |  |
| 3+3 | selection (%) | 14.4 | 35.2 | **28.1** | 11.4 | 15.8 | 3.1 | 0 | 67.3 | 12.2 |
|  | pts at MTD | 3.8 | 3.5 | **3.2** | 1.7 |  |  |  |  |  |
| Keyboard | selection (%) | 7.9 | 27.4 | **45.9** | 18.5 | 29.4 | 0.4 | 0 | 27.7 | 22.6 |
|  | pts at MTD | 6.1 | 7.6 | **6.2** | 2.8 |  |  |  |  |  |
| MEM-Keyboard | selection (%) | 5.5 | 17.7 | **51.2** | 25.4 | 28.9 | 0.1 | 0 | 29 | 22.5 |
|  | pts at MTD | 3 | 3.4 | **7.6** | 8.6 |  |  |  |  |  |
| TITE-Keyboard | selection (%) | 13.7 | 29.4 | **42** | 14.4 | 18.2 | 0.4 | 0 | 42.9 | 21 |
|  | pts at MTD | 6.3 | 6.7 | **5.7** | 2.3 |  |  |  |  |  |
| TITE-Keyboard (E) | selection (%) | 14.6 | 29.6 | **41.2** | 14.1 | 18 | 0.4 | 0 | 44.9 | 20.9 |
|  | pts at MTD | 6.5 | 6.7 | **5.5** | 2.2 |  |  |  |  |  |
| MEM-TITE-Keyboard | selection (%) | 5 | 25.4 | **50.6** | 18.7 | 20.1 | 0.3 | 0 | 15.7 | 23.1 |
|  | pts at MTD | 5.6 | 7.1 | **7.7** | 2.6 |  |  |  |  |  |
| MEM-TITE-Keyboard (E) | selection (%) | 5.1 | 25.6 | **51.9** | 17 | 19.9 | 0.3 | 0 | 16.6 | 22.9 |
|  | pts at MTD | 5.6 | 7.1 | **7.8** | 2.4 |  |  |  |  |  |

**Table S7. (Continued)**

| Methods |  | dose level | | | | Duration | Stop (%) | Overdose (%) | Risk of Poor allocation (%) | Sample size |
| --- | --- | --- | --- | --- | --- | --- | --- | --- | --- | --- |
|  |  | 1 | 2 | 3 | 4 |  |  |  |  |  |
| Scenario 13 | p.true | 0.11 | 0.14 | **0.28** | 0.41 |  |  |  |  |  |
| 3+3 | selection (%) | 14.4 | 35.2 | **28.1** | 11.4 | 15.8 | 3.1 | 0 | 67.3 | 12.2 |
|  | pts at MTD | 3.8 | 3.5 | **3.2** | 1.7 |  |  |  |  |  |
| Keyboard | selection (%) | 7.9 | 27.4 | **45.9** | 18.5 | 29.4 | 0.4 | 0 | 27.7 | 22.6 |
|  | pts at MTD | 6.1 | 7.6 | **6.2** | 2.8 |  |  |  |  |  |
| MEM-Keyboard | selection (%) | 5.5 | 17.5 | **56.5** | 20.3 | 29.6 | 0.2 | 0 | 7.7 | 23 |
|  | pts at MTD | 3 | 3.5 | **9.5** | 7.1 |  |  |  |  |  |
| TITE-Keyboard | selection (%) | 13.7 | 29.4 | **42** | 14.4 | 18.2 | 0.4 | 0 | 42.9 | 21 |
|  | pts at MTD | 6.3 | 6.7 | **5.7** | 2.3 |  |  |  |  |  |
| TITE-Keyboard (E) | selection (%) | 14.6 | 29.6 | **41.2** | 14.1 | 18 | 0.4 | 0 | 44.9 | 20.9 |
|  | pts at MTD | 6.5 | 6.7 | **5.5** | 2.2 |  |  |  |  |  |
| MEM-TITE-Keyboard | selection (%) | 5 | 25.5 | **51.4** | 17.8 | 20 | 0.3 | 0 | 15.4 | 23 |
|  | pts at MTD | 5.6 | 7.1 | **7.9** | 2.4 |  |  |  |  |  |
| MEM-TITE-Keyboard (E) | selection (%) | 5.1 | 25.3 | **52.3** | 16.9 | 19.7 | 0.4 | 0 | 15.4 | 22.9 |
|  | pts at MTD | 5.6 | 7 | **8.1** | 2.2 |  |  |  |  |  |

The MTD is in boldface. 3+3 is the conventional 3+3 design; Keyboard is the Keyboard design; MEM-Keyboard is the proposed design that incorporates the historical data; TITE-Keyboard and TITE-Keyboard (E) are the time-to-event keyboard designs that utilizes the approximated or exact likelihood function respectively; MEM-TITE-Keyboard and MEM-TITE-Keyboard (E) are the proposed designs. π denotes the different prior exchangeability probabilities.

## Figure S1. Simulation results with sample size of 24 and cohort size of 3. TITE-Keyboard and TITE-Keyboard (E) are the time-to-event keyboard designs that utilizes the approximated or exact likelihood function respectively; MEM-TITE-Keyboard and MEM-TITE-Keyboard (E) are the proposed designs that utilizes the approximated or exact likelihood function respectively.


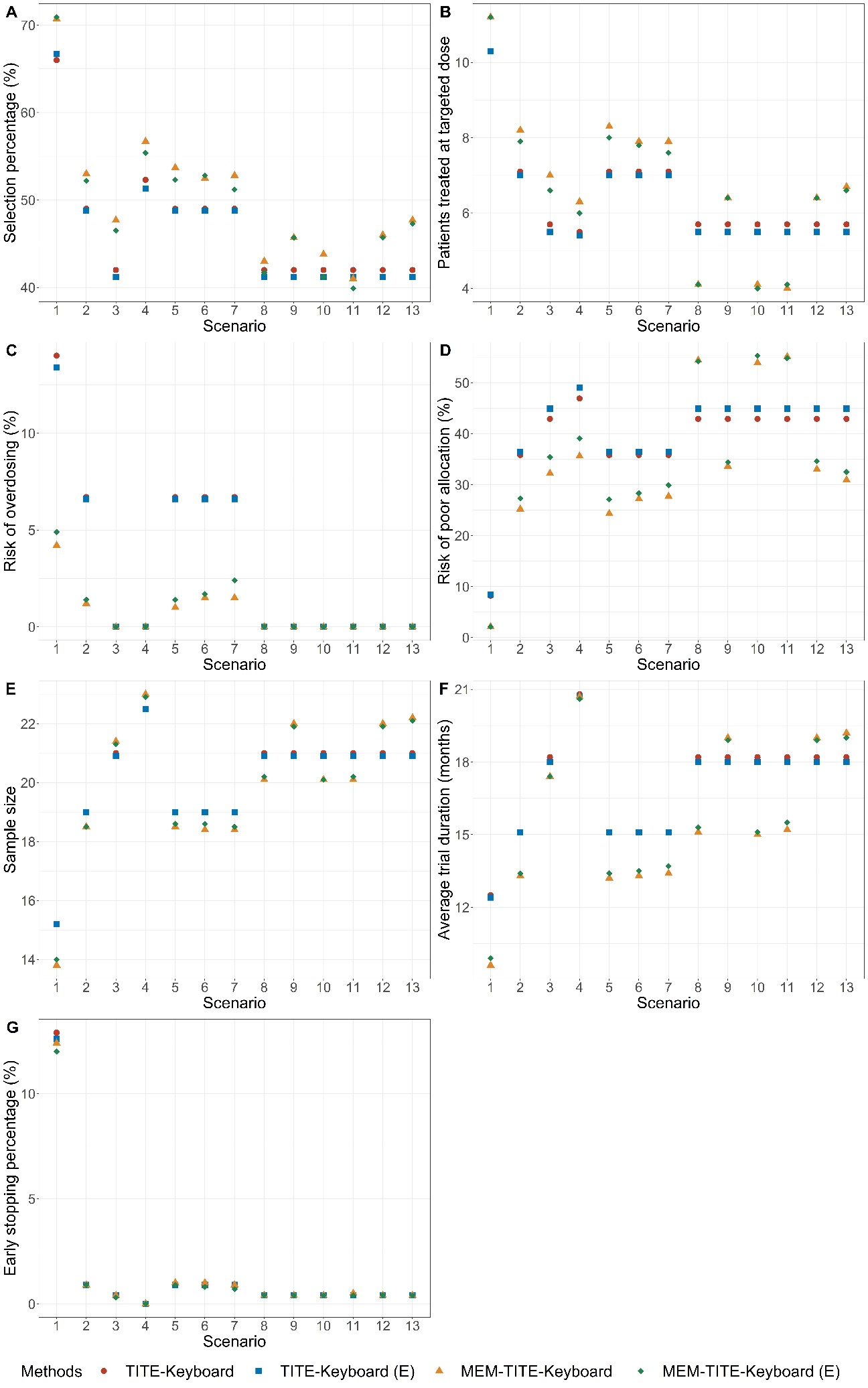

Supplement: Supplementary file 3 [file DataSheet1.docx]
